# Safety and activity of WX-0593 (Iruplinalkib) in patients with *ALK*- or *ROS1*-rearranged advanced non-small cell lung cancer: a phase 1 dose-escalation and dose-expansion trial

**DOI:** 10.1038/s41392-021-00841-8

**Published:** 2022-01-28

**Authors:** Yuankai Shi, Jian Fang, Xuezhi Hao, Shucai Zhang, Yunpeng Liu, Lin Wang, Jianhua Chen, Yi Hu, Xiaosheng Hang, Juan Li, Chunling Liu, Yiping Zhang, Zhehai Wang, Yanping Hu, Kangsheng Gu, Jian’an Huang, Liangming Zhang, Jinlu Shan, Weiwei Ouyang, Yanqiu Zhao, Wu Zhuang, Yan Yu, Jun Zhao, Helong Zhang, Pei Lu, Weidong Li, Meimei Si, Mingjing Ge, Huaize Geng

**Affiliations:** 1 National Cancer Center/National Clinical Research Center for Cancer/Cancer Hospital, Chinese Academy of Medical Sciences & Peking Union Medical College, Beijing Key Laboratory of Clinical Study on Anticancer Molecular Targeted Drugs, 100021 Beijing, P.R. China; 2 Beijing Cancer Hospital, 100142 Beijing, P.R. China; 3 Beijing Chest Hospital, Capital Medical University, 101149 Beijing, P.R. China; 4 The First Hospital of China Medical University, 110001 Shenyang, P.R. China; 5 Hunan Cancer Hospital, 410000 Changsha, P.R. China; 6 Chinese PLA General Hospital, 100853 Beijing, P.R. China; 7 Affiliated Hospital of Jiangnan University, 214062 Wuxi, P.R. China; 8 Sichuan Cancer Hospital, 610041 Chengdu, P.R. China; 9 Affiliated Tumour Hospital of Xinjiang Medical University, 830000 Urumqi, P.R. China; 10 Zhejiang Cancer Hospital, 310022 Hangzhou, P.R. China; 11 Shandong Cancer Hospital and Institute, Shandong First Medical University and Shandong Academy of Medical Sciences, 250117 Jinan, P.R. China; 12 Hubei Cancer Hospital, 430079 Wuhan, P.R. China; 13 The First Affiliated Hospital of Anhui Medical University, 230022 Hefei, P.R. China; 14 The First Affiliated Hospital of Soochow University, 215006 Suzhou, P.R. China; 15 Yantai Yuhuagding Hospital, Yantai, 264000 P.R. China; 16 Army Medical Center of People’s Liberation Army, 400042 Chongqing, P.R. China; 17 Guizhou Cancer Hospital, 520100 Guiyang, P.R. China; 18 Henan Cancer Hospital, 450008 Zhengzhou, P.R. China; 19 Fujian Province Cancer Hospital, 350014 Fuzhou, P.R. China; 20 Harbin Medical University Cancer Hospital, 150081 Harbin, P.R. China; 21 Tangdu Hospital of the Fourth Military Medical University, 710038 Xi’an, P.R. China; 22 Liuzhou People’s Hospital, 545006 Liuzhou, P.R. China; 23 Cancer Center of Guangzhou Medical University, 510095 Guangzhou, P.R. China; 24 Qilu Pharmaceutical Co., Ltd, 250101 Jinan, P.R. China

**Keywords:** Lung cancer, Lung cancer

## Abstract

WX-0593 (Iruplinalkib) is a novel, highly selective oral ALK and ROS1 tyrosine kinase inhibitor (TKI). In this study, the safety, antitumor activity, and pharmacokinetics of WX-0593 were evaluated in advanced non-small cell lung cancer (NSCLC) patients with *ALK* or *ROS1* rearrangement. In the dose-escalation phase and dose-expansion phase, patients were treated with WX-0593 until disease progression, unacceptable toxicity, or subject withdrawal. In the dose-escalation phase, the primary endpoints were maximum tolerated dose (MTD), dose-limiting toxicity (DLT), and safety assessed by investigators. In the dose-expansion phase, the primary endpoint was objective response rate (ORR) assessed by investigators. Between September 25, 2017 and October 15, 2018, a total of 153 patients received WX-0593 treatment. Two dose-limiting toxicities (DLTs) including one grade 3 QT interval prolonged and one grade 2 chronic heart failure were reported at the dose of 300 mg in one patient. MTD was not reached. Overall, 140 of the 152 (92%) patients experienced treatment-related adverse events (TRAEs) and 35 of the 152 (23%) patients had TRAEs ≥grade 3. The overall ORR was 59.3% (32 of 54) for the dose-escalation phase and 56.6% (56 of 99) for the dose-expansion phase. For patients who were *ALK*-rearranged and ALK TKI naive, the ORR were 81.0% (17 of 21) in the dose-escalation phase and 76.3% (29 of 38) in the dose-expansion phase, and for patients who previously received crizotinib as the only ALK TKI, the ORR were 38.1% (8 of 21) and 45.7% (21 of 46) for the two phases, respectively. For patients who were *ROS1*-rearranged, the ORR were 30.0% (3 of 10) in the dose-escalation phase and 44.4% (4 of 9) in the dose-expansion phase. WX-0593 showed favorable safety and promising antitumor activity in advanced NSCLC patients with *ALK* or *ROS1* rearrangement.

## Introduction

Gene fusions that lead to overexpression of anaplastic lymphoma kinase (ALK) protein, originally identified in anaplastic large cell lymphoma, occurred in 3–7% of unselected non-small cell lung cancer (NSCLC) patients.^1,2^ Chromosomal rearrangements involving ROS proto-oncogene, receptor tyrosine kinase 1 (ROS1), another therapeutically tractable oncogenic driver, have been found in 1–2% of patients with NSCLC.^3,4^ Both *ALK* and *ROS1* rearrangements can led to expression of oncogenic fusion proteins with constitutive kinase activity, with each defining a specific molecular subset of NSCLC which displays high sensitivity to *ALK* or *ROS1* inhibition.

Crizotinib, a tyrosine kinase inhibitor (TKI) targeting *ALK*, *ROS1* as well as *MET*, was the first drug approved by the Food and Drug Administration (FDA) for patients with advanced *ALK*-rearranged or *ROS1*-rearranged NSCLC. For NSCLC patients with *ALK* rearrangement receiving prior chemotherapy, crizotinib showed clinically meaningful efficacy, and the objective response rates (ORRs) were around 60% and median progression-free survival (PFS) were 8–10 months across studies.^5–7^ In the first-line setting, a randomized phase III trial demonstrated the superiority of crizotinib over platinum-double chemotherapy (ORR: 74% vs. 45%, PFS: 10.9 vs. 7.0 months).^7^ As for *ROS1*-rearranged NSCLC, critizotinib showed marked efficacy, yielding an ORR of 72% and median PFS of 19.2 months.^8^ Despite the remarkable antitumor activity of crizotinib, almost all patients become inevitably resistant to crizotinib within 1–2 years of treatment, and central nervous system (CNS) metastases during treatment were a frequent manifestation of acquired resistance to crizotinib, probably due to poor drug penetration through the blood–brain barrier.^9,10^

Several second-generation (alectinib, ceritinib, brigatinib, and ensartinib) and third-generation (lorlatinib) ALK TKIs, generally more potent than crizotinib, have been developed to treat patients with resistance to crizotinib. In the first-line setting, alectinib and brigatinib have exhibited advantages than crizotinib^11–13^ and were approved by FDA as the standard first-line treatment for NSCLC patients with *ALK* rearrangement. Based on a phase 2 trial,^14^ lorlatinib was approved by FDA for patients with *ALK*-positive NSCLC who failed two previous ALK TKIs. A recent study demonstrated the superiority of lorlatinib over crizotinib as the first-line treatment in patients with advanced ALK-positive NSCLC.^15^ Also, lorlatinib showed favorable efficacy in patients with advanced *ROS1*-rearranged NSCLC, including crizotinib-pretreated patients and those with CNS metastases.^16^

WX-0593 (Iruplinalkib) (Qilu Pharmaceutical Co., Ltd., Jinan, China) is a novel, highly selective oral TKI that targets *ALK* and *ROS1*. The molecular structure of WX-0593 is shown in Supplementary Fig. S1. In-vitro kinase assays, WX-0593 inhibited the kinase activity of both wild-type *ALK* and *ALK*-resistant mutants (including L1196M and C1156Y), and epidermal growth factor receptor (*EGFR*) L858R/T790M mutation, which was comparable to brigatinib (data unpublished). In preclinical xenograft studies of NSCLC that harbored *ALK* rearrangements, WX-0593 demonstrated potent antitumor activity against both crizotinib-sensitive and crizotinib-resistant tumors. In cell-line models, WX-0593 had superior inhibitory activity against the *ALK*- resistant mutants (including *ALK*-G1202R mutants), *ROS1* wild-type and mutants resistant to crizotinib (except for *ROS1*-G2032R and *ROS1*-L1951R mutants) at therapeutic concentration when administered 180 mg, once daily (Supplementary Material). These preclinical results indicated that WX-0593 might be a potentially potent therapeutic agent for patients with *ALK*- or *ROS1*-rearranged NSCLC.

Here we report the results of the first-in-human (FIH) phase 1 study of WX-0593, which was conducted to explore the maximum tolerated dose (MTD), safety, antitumor activity, and pharmacokinetics (PK) of WX-0593 in advanced NSCLC patients with *ALK* or *ROS1* rearrangement.

## Results

### Patient baseline characteristics

**Fig. 1:**
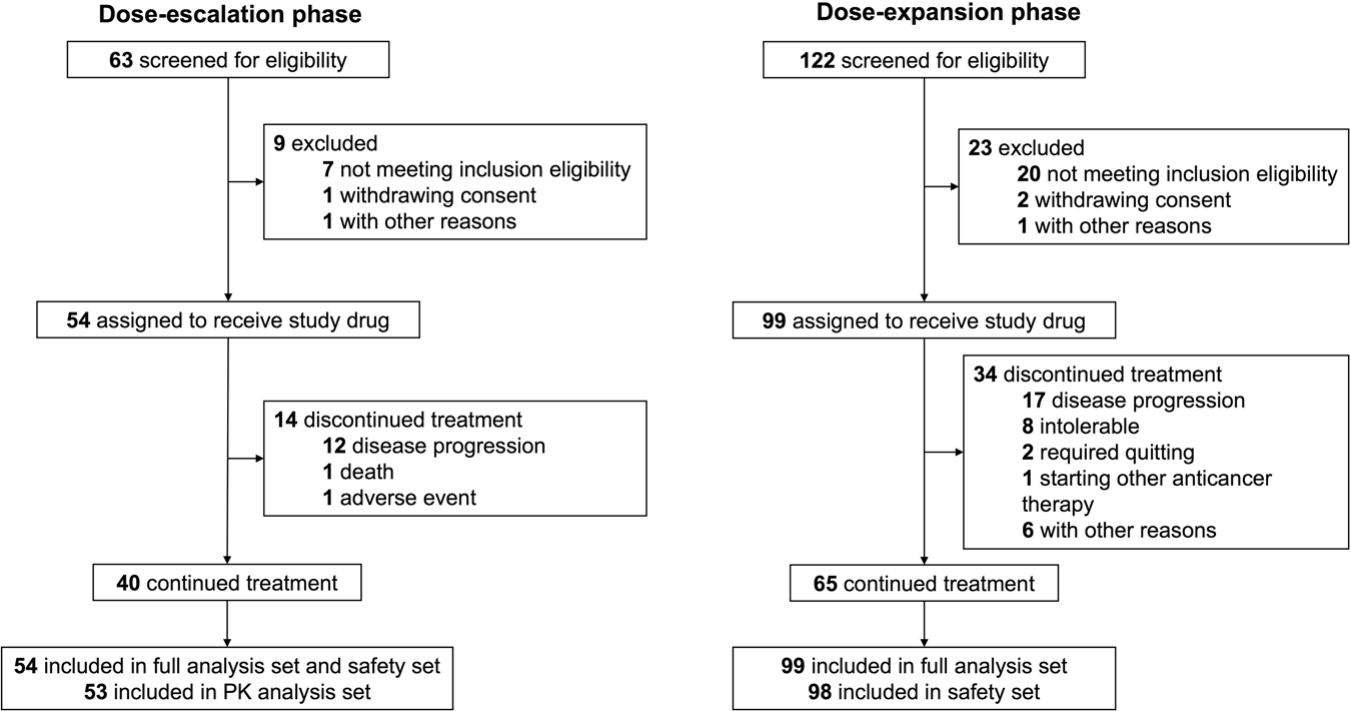
CONSORT flow chart. CONSORT indicates Consolidated Standards of Reporting Trials

Between September 25, 2017 and October 15, 2018, a total of 153 patients received WX-0593 treatment, with 54 patients in the dose-escalation phase and 99 patients in the dose-expansion phase. The data cut-off date was December 26, 2018 for the dose-escalation phase and September 10, 2019 for the dose-expansion phase. The median follow-up was 160 (95% confidence interval [CI] 106–175) days and 203 (95% CI 194–219) days for the dose-escalation and dose-expansion phases, respectively. As of the data cut-off date, 40 of the 54 (74.0%) patients and 65 of the 99 (65.7%) patients in the dose-escalation and dose-expansion phases, respectively, remained on treatment (Fig. 1). Of all the 153 patients in full analysis set (FAS), 140 (91.5%) patients were with adenocarcinoma while 4 (2.6%) patients were with squamous cell carcinoma. A total of 150 patients were tested for *ALK* rearrangement status and 80 patients were tested for *ROS1* rearrangement status. Overall, 137 of the 153 (89.5%) patients were *ALK* rearrangement positive and 19 of the 153 (12.4%) patients were *ROS1* rearrangement positive. There were three patients with both *ALK* and *ROS1* rearrangement positive (two in the dose-escalation phase and one in the dose-expansion phase). A total of 52.9% (81 of 153) of patients had baseline CNS metastasis. Overall, 82.3% (126 of 153) of patients received >1 line of prior anticancer therapy. Of note, for prior ALK inhibitor treatment, 46.4% (71 of 153) of patients received only crizotinib and 2.0% (3 of 153) received other ALK TKIs only. Nine (5.9%) patients received both crizotinib and other second-generation ALK TKIs. The patient characteristics are presented in Table 1.

**Table 1:** Patient baseline characteristics

Characteristics	Dose-escalation cohort (*n* = 54)	Dose-expansion cohort (*n* = 99)	Total (*n* = 153)
Median age (range), years	51.6 (28–69)	50.2 (24–70)	NA
≤60, *n* (%)	45 (83.3)	80 (80.8)	125 (81.7)
>60, *n* (%)	9 (16.7)	19 (19.2)	28 (18.3)
Sex, *n* (%)			
Male	21 (38.9)	49 (49.5)	70 (45.8)
Female	33 (61.1)	50 (50.5)	83 (54.2)
Tumor histology, *n* (%)			
Squamous cell carcinoma	2 (3.7)	2 (2.0)	4 (2.6)
Adenocarcinoma	49 (90.7)	91 (91.9)	140 (91.5)
Other^a^	3 (5.6)	6 (6.1)	9 (5.9)
ECOG PS, *n* (%)			
0	20 (37.0)	30 (30.3)	50 (30.7)
1	34 (63.0)	69 (69.7)	103 (67.3)
*ALK* rearrangement status			
Positive	46 (85.2)^b^	91 (91.9)^c^	137 (89.5)
Negative	8 (14.8)	5 (5.1)	13 (8.5)
Untested	0 (0)	3(3.0)	3 (2.0)
*ROS1* rearrangement status			
Positive	10 (18.5)^b^	9 (9.1)^c^	19 (12.4)
Negative	23 (42.6)	38 (38.4)	61 (39.9)
Untested	21 (38.9)	52 (52.5)	73 (47.7)
CNS measurable or non-measurable metastasis			
Yes	22 (40.7)	58 (58.6)	81 (52.9)
No	32 (59.3)	41 (41.4)	72 (47.1)
≥1 line of prior anti-cancer therapy	43 (79.6)	83 (83.8)	126 (82.3)
Prior ALK TKIs			
ALK TKI naive	28 (51.9)	42 (42.4)	70 (45.8)
Crizotinib only	21 (38.9)	50 (50.5)	71 (46.4)
Other ALK TKI only	1 (1.9)	2 (2.0)	3 (2.0)
Both crizotinib^d^ and other second-generation ALK TKI	4 (7.4)	5 (5.1)	9 (5.9)

*ECOG* Eastern Cooperative Oncology Group, *PS* performance status, *ALK* anaplastic lymphoma kinase, *ROS1* c-ros oncogene 1 receptor kinase, *CNS* central nervous system, *TKI* tyrosine kinase inhibitor, *NA* not available

^a^Others include large cell carcinoma, adenosquamous carcinoma and other undefined pathological subtypes

^b^Two patients with both ALK and ROS1 rearrangement positive status in dose-escalation phase

^c^One patient with both ALK and ROS1 rearrangement positive status in dose-expansion phase

^d^One of the four patients received crizotinib generics

### Safety

Patients in the dose-escalation phase were treated with WX-0593 at dose levels of 30–300 mg. Two dose-limiting toxicities (DLTs) including grade 3 QT interval prolonged and grade 2 chronic heart failure were reported at the dose of 300 mg in 1 patient. All the DLTs were resolved after WX-0593 treatment halted and systematic treatment was applied. MTD could not be identified as only 3 patients were enrolled in the 300 mg dose group and the dose-escalation phase was ceased due to DLTs occurring in 1 of the 3 patients. The doses recommended for the dose-expansion phase were 120 and 180 mg.

Of the 153 patients, a total of 152 patients entered in safety set (SS) with one patient excluded from SS due to safety recording hiatus. Of the 152 patients, at least one treatment-emergent adverse event (TEAE) occurred in 148 (97%) patients. Most of the TEAEs were grade 1–2 and 53 (35%) patients had adverse events (AEs) >grade 3. Serious adverse events (SAEs) occurred in 25 (16%) patients (Supplementary Table S2). The most common TEAEs (≥10%) were vomiting (34%), nausea (32%), hypercholesterolemia (31%), aspartate aminotransferase (AST) elevated (31%), alanine aminotransferase (ALT) elevated (30%), hypertriglyceridemia (24%), hypertension (22%), creatine phosphokinase (CPK) elevated (20%), diarrhea (14%), hyperlipidemia (14%), rash (13%), dizziness (11%), γ-glutamyl transpeptidase (γ-GGT) elevated (11%), and upper respiratory infection (11%) (Table 2).

**Table 2:** Treatment-emergent adverse events reported in ≥10% of patients by dose and severity

Treatment-emergent adverse event	30 mg (*n* = 3)		60 mg (*n* = 3)		90 mg (*n* = 8)		120 mg (*n* = 61)		180 mg (*n* = 61)		240 mg (*n* = 13)		300 mg (*n* = 3)		Total (*n* = 152)		
	Grade 1–2	≥Grade 3	Grade 1–2	≥Grade 3	Grade 1–2	≥Grade 3	Grade 1–2	≥Grade 3	Grade 1–2	≥Grade 3	Grade 1–2	≥Grade 3	Grade 1–2	≥Grade 3	All grade	Grade 1–2	≥Grade 3
Vomiting	1 (33%)	0	1 (33%)	0	2 (25%)	0	11 (18%)	0	18 (30%)	0	7 (54%)	0	2 (67%)	0	52 (34%)	52 (34%)	0
Nausea	0	0	2 (67%)	0	4 (50%)	0	12 (20%)	0	21 (34%)	0	8 (62%)	0	1 (33%)	0	48 (32%)	48 (32%)	0
Hypercholesterolemia	0	0	0	0	2 (25%)	0	16 (26%)	3 (5%)	12 (20%)	2 (3%)	10 (77%)	0	2 (67%)	0	47 (31%)	42 (28%)	5 (3%)
AST elevated	0	0	0	0	3 (38%)	0	15 (26%)	2 (3%)	18 (30%)	3 (5%)	5 (38%)	1 (1%)	0	0	47 (31%)	41 (27%)	6 (4%)
ALT elevated	0	0	0	0	3 (38%)	0	15 (26%)	3 (5%)	17 (28%)	2 (3%)	4 (31%)	1 (1%)	0	0	45 (30%)	39 (26%)	6 (4%)
Hypertriglyceridemia	0	0	0	0	2 (25%)	0	14 (23%)	3 (5%)	9 (15%)	2 (3%)	6 (46%)	0	1 (33%)	0	37 (24%)	32 (21%)	5 (3%)
Hypertension	0	0	1 (33%)	0	0	2 (1%)	8 (13%)	1 (2%)	9 (15%)	3 (5%)	5 (38%)	3 (2%)	1 (33%)	0	33 (22%)	24 (16%)	9 (6%)
CPK elevated	0	0	1 (33%)	0	2 (25%)	0	8 (13%)	0	18 (30%)	0	1 (8%)	0	1 (33%)	0	31 (20%)	31 (20%)	0
Diarrhea	0	0	0	0	0	0	3 (5%)	0	10 (16%)	1 (2%)	6 (46%)	0	2 (67%)	0	22 (14%)	21 (14%)	1 (1%)
Hyperlipidemia	1 (33%)	0	0	1 (33%)	3 (38%)	0	5 (8%)	0	11 (18%)	0	0	0	1 (33%)	0	22 (14%)	21 (14%)	1 (1%)
Rash	0	0	0	0	1 (13%)	0	5 (8%)	0	10 (16%)	1 (2%)	3 (23%)	0	0	0	20 (13%)	19 (13%)	1 (1%)
Dizziness	0	0	0	0	1 (13%)	0	5 (8%)	0	10 (16%)	0	2 (15%)	0	1 (33%)	0	16 (11%)	16 (11%)	0
γ-GGT elevated	0	0	0	0	1 (13%)	0	3 (5%)	3 (5%)	8 (13%)	1 (2%)	0	1 (1%)	0	0	17 (11%)	12 (8%)	5 (3%)
Upper respiratory infection	0	0	0	0	1 (13%)	0	8 (13%)	0	7 (11%)	1 (2%)	0	0	0	0	17 (11%)	16 (11%)	1 (1%)
Headache	0	0	0	0	2 (25%)	0	7 (11%)	0	3 (5%)	0	2 (15%)	0	0	0	14 (9%)	14 (9%)	0

*AST* aspartate transaminase, *ALT* alanine aminotransferase, *CPK* creatine phosphokinase, *γ-GGT* γ-glutamyl transpeptidase

Treatment-related adverse events (TRAEs), assessed by the investigators, occurred in 140 of the 152 (92%) patients. The most common TRAEs (≥10%) were AST elevated (30%), ALT elevated (29%), nausea (28%), hypercholesterolemia (27%), vomiting (24%), hypertriglyceridemia (21%), hypertension (17%), CPK elevated (16%), diarrhea (15%), rash (11%), γ-GGT elevated (10%), and hyperlipidemia (10%). The majority were grade 1–2. Grade 3–5 TRAEs occurred in 35 of the 152 (23%) patients, with the most common being hypertension (5%), ALT elevated (4%), AST elevated (3%), hypertriglyceridemia (3%), and γ-GGT elevated (3%). All other TRAEs >grade 3 were reported in one patient each (Supplementary Table S3). Treatment-related SAEs occurred in 10 of the 152 (7%) patients (Supplementary Table S2). Detailed TRAEs across different dosing cohorts are summarized in Supplementary Table S3.

Dose reduction and dose interruption attributable to drug-related AEs occurred in 4 (3%) and 30 (20%) patients, respectively. WX-0593 was permanently discontinued in 8 of 152 (5%) patients due to TRAEs, including QT interval prolonged (*n* = 1, 300 mg), interstitial pneumonia (*n* = 2, 120 mg), respiratory failure (*n* = 1, 120 mg), AST elevated (*n* = 1, 120 mg), infectious pneumonia (*n* = 1, 180 mg), rash (*n* = 1, 180 mg), and acute and transient psychotic disorders (*n* = 1, 180 mg). Seven patients died (*n* = 1, disease progression; *n* = 1, anoxia; *n* = 1, intracranial hemorrhage; *n* = 2, infectious pneumonia; *n* = 2, respiratory failure; *n* = 1, intra-abdominal hemorrhage) while on study. Two of the deaths (one due to infectious pneumonia and one due to respiratory failure, all within 48 h of WX-0593 treatment) occurred among patients who received the regimen of once-daily 120 mg without a 7-day lead-in at the dose of 60 mg in the dose-expansion phase, which were considered as possibly related to WX-0593.

### Efficacy

#### Dose-escalation phase (*n* = 54)

The overall ORR was 59.3% (32 of 54, 95% CI 45.0–72.4%; all achieved partial response [PR]). The ORRs in 30, 60, 90, 120, 180, 240, and 300 mg dosing cohorts were 66.7, 66.7, 37.5, 72.7, 69.2, 53.8, and 33.3%, respectively. The overall disease control rate (DCR) was 87.0% (47 of 54). For *ALK*-rearranged patients, the overall ORR was 63.0% (29 of 46, 95% CI 47.5–76.8%) and DCR was 89.1% (41 of 46, 95% CI 76.4–96.4%) (Supplementary Table S4). For *ROS1*-rearranged patients, the overall ORR was 30.0% (3 of 10, 95% CI 6.7–65.2%) and DCR was 80.0% (8 of 10, 95% CI 44.4–97.5%).

**Fig. 2:**
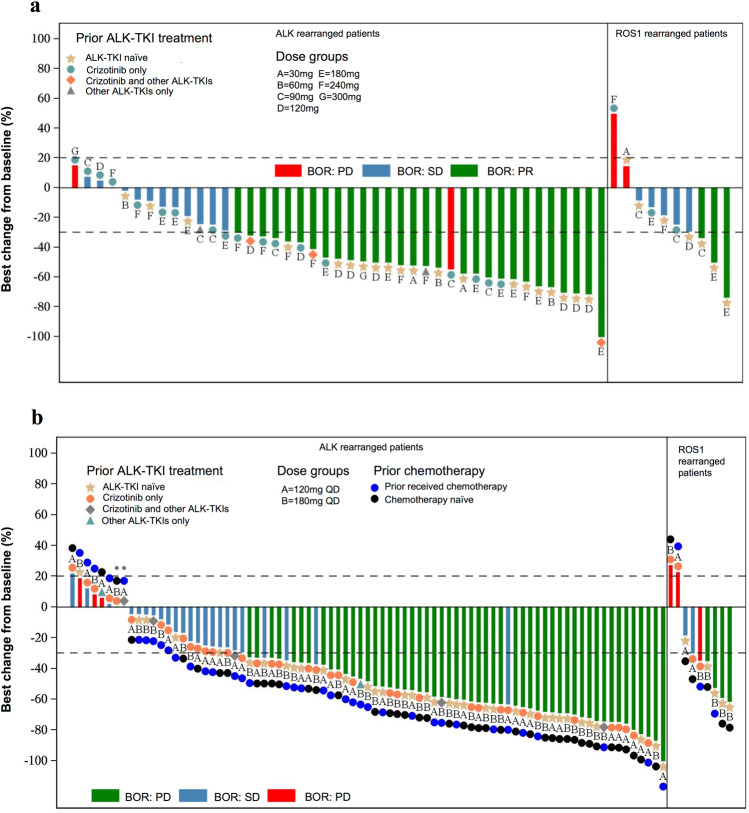
Best percentage change from baseline in target lesion size for patients in the **a** dose-escalation phase (*n* = 51) and **b** dose-expansion phase (*n* = 89). BOR best overall response, PR partial response, SD stable disease, PD disease progression, ALK anaplastic lymphoma kinase, ROS1 ROS proto-oncogene, receptor tyrosine kinase 1. *The percentage of best change from baseline was 0%. Note: Among 54 patients in the full analysis of dose-escalation phase, three patients did not have any efficacy assessment results. Two patients in the dose-escalation phase presented with both *ALK* and *ROS1* rearrangement positive status, thus duplicately displayed in the waterfall plot. Therefore, a total of 53 bars for 51 patients are displayed in the waterfall plot **a**. Among 99 patients in the full analysis set of dose-expansion phase, 5 patients did not have any efficacy assessment results, 2 patients did not have measurable target lesion at baseline, and 3 patients did not have any efficacy evaluation results due to adverse events. One patient in the dose-expansion phase presented with both *ALK* and *ROS1* rearrangement positive status, thus duplicately displayed in the waterfall plot. Therefore, a total of 90 bars for 89 patients are displayed in the waterfall plot **b**

Of note, for all patients who were ALK TKI naive, the ORR was 71.4% (20 of 28, 95% CI 51.3–86.8%) and for patients who received only crizotinib before, the ORR was 38.1% (8 of 21, 95% CI 18.1–61.6%) (Supplementary Table S4). Tumor shrinkage was observed in most patients (Fig. 2a).

#### Dose-expansion phase (*n* = 99)

The overall ORR for dose-expansion phase was 56.6% (56 of 99, 95% CI 46.2–66.5%; all achieving PR). DCR was 83.8% (83 of 99, 95% CI 75.1–90.5%). The ORRs in the 120 and 180 mg groups were 50% (25 of 50, 95% CI 35.5–64.5%) and 63.3% (31 of 49, 48.3–76.6%), respectively (Table 3). Tumor shrinkage was observed in most patients (Fig. 2b).

**Table 3:** Summary of efficacy by dose and *ALK* and *ROS1* rearrangement status in the dose-expansion phase

	120 mg	180 mg	Total
*All patients (n)*	50	49	99
ORR (95% CI)	50.0% (35.5%, 64.5%)	63.3% (48.3%, 76.6%)	56.6% (46.2%, 66.5%)
DCR (95% CI)	82.0% (68.6%, 91.4%)	85.7% (72.8%, 94.1%)	83.8% (75.1%, 90.5%)
DOR (mo)			
Median (95% CI)	NR (5.52, NR)	NR (5.49, NR)	NR (5.65, NR)
TTP (mo)			
Median (95% CI)	NR (6.87, NR)	NR (NR, NR)	NR (6.93, NR)
PFS (mo)			
Number of events (%)	15 (30.0%)	10 (20.4%)	25 (25.2%)
Median (95% CI)	NR (6.83, NR)	NR (NR, NR)	NR (6.93, NR)
*ALK-positive patients (n)*	47	44	91
ORR (95% CI)	53.2% (38.1%, 67.9%)	63.6% (47.8%, 77.6%)	58.2% (47.4%, 68.5%)
DCR (95% CI)	83.0% (69.2%, 92.4%)	88.6% (75.4%, 96.2%)	85.7% (76.8%, 92.2%)
DOR (mo)			
Median (95% CI)	NR (5.52, NR)	NR (5.49, NR)	NR (5.65, NR)
TTP (mo)			
Median (95% CI)	NR (6.87, NR)	NR (NR, NR)	NR (6.93, NR)
PFS (mo)			
Number of events (%)	12 (25.5%)	8 (18.2%)	20 (22.0%)
Median (95% CI)	NR (6.87, NR)	NR (NR, NR)	NR (6.93, NR)
*ROS1-positive patients (n)*	3	6	9
ORR (95% CI)	0 (0%, 70.8%)	66.7% (22.3%, 95.7%)	44.4% (13.7%, 78.8%)
DCR (95% CI)	66.7% (9.4%, 99.2%)	66.7% (22.3%, 95.7%)	66.7% (29.9%, 92.5%)
DOR (mo)			
Median (95% CI)	NR (NR, NR)	NR (NR, NR)	NR (NR, NR)
TTP (mo)	3 (100%)	2 (33.3%)	5 (55.6%)
Median (95% CI)	2.76 (1.35, 2.76)	NR (1.35, NR)	2.76 (1.35, NR)
PFS (mo)			
Number of events (%)	3 (100%)	2 (33.3%)	5 (55.6%)
Median (95% CI)	2.76 (1.35, 2.76)	NR (1.35, NR)	2.76 (1.35, NR)

*ORR* objective response rate, *CI* confidence interval, *DCR* disease control rate, *DOR* duration of response, *TTP* time to progression, *PFS* progression-free survival, *NR* not reached

At the data cut-off date of September 10, 2019, among the 99 patients in FAS, 56 (56.6%) patients achieved PR, 27 (27.3%) patients attained stable disease (SD) and 8 (8.1%) patients experienced progressive disease. The overall median duration of response (DOR) was not reached (NR) (95% CI 5.52 months to NR), and the median PFS was also not reached (95% CI 6.93 months to NR) with 25 of the 99 (25.2%) patients having progression event (Supplementary Fig. S3).

For *ALK*-rearranged patients, 53 of the 91 (58.2%, 95% CI 47.4–68.5%) patients achieved objective response, with all these patients achieving PR. Twenty-five patients achieved SD and the DCR was 85.7% (78 of 91, 95% CI 76.8 to 92.2%). PFS was not reached (95% CI 6.93 months to NR). For *ALK*-rearranged patients who were ALK TKI naive, the ORR was 76.3% (29 of 38, 95% CI 59.8–88.6%), of whom the ORR for chemotherapy-naive patients was 85.0% (17 of 20, 95% CI 62.1–96.8%) and 66.7% (12 of 18, 95% CI 41.0–86.7%) for those who received chemotherapy. The median PFS for this subgroup was 6.9 months (95% CI 4.1 to NR). For *ROS1*-rearranged patients, the ORR was 44.4% (4 of 9, 95% CI 13.7–78.8%) and the DCR was 66.7% (6 of 9, 95% CI 29.9–92.5%). For *ROS1*-rearranged patients who were crizotinib naive, 80.0% (4 of 5, 95% CI 28.4–99.5%) achieved objective response (Supplementary Table S5).

For the analysis about the efficacy of different prior ALK TKI treatment, ORR differed in subgroups. For patients who were ALK TKI naive, ORR was 76.2% (32 of 42, 95% CI 60.5–87.9%) and DCR was 92.9% (39 of 42, 95% CI 80.5–98.5%). Among those who received crizotinib as the only ALK TKI treatment, the ORR was 42.0% (21 of 50, 95% CI 28.2–56.8%) and the DCR was 76.0% (38 of 50, 95% CI 61.8–86.9%). For patients receiving both crizotinib and other ALK TKIs, the ORR was 40.0% (2 of 5, 95% CI 5.3–85.3%) and DCR was 100% (5 of 5, 95% CI 47.8–100.0%). Median PFS was 6.83 months for patients previously only receiving crizotinib while PFS for other two subgroups were both not reached (Supplementary Table S6).

For patients with baseline measurable or non-measurable brain metastasis, the overall intracranial ORR was 22.4% (13 of 58, 95% CI 12.5–35.3%), with 6 patients achieving complete remission (CR) and 7 patients receiving PR, and the median PFS was not reached (95% CI 6.90 months to NR). For patients with baseline measurable brain metastasis, 7 of 15 (46.7%, 95% CI 21.3–73.4%) patients had an intracranial objective response. Of these patients, those who received crizotinib before yielded an intracranial ORR of 50.0% (5 of 10, 95% CI 18.7–81.3%) and those who were ALK TKI naive achieved an intracranial ORR of 50.0% (2 of 4, 95% CI 6.8–93.2%). Regarding the influence of CNS radiotherapy history on efficacy for patients with baseline measurable CNS metastasis, the intracranial ORR were 28.6% (2 of 7, 95% CI 3.7–71.0%) and 62.5% (5/8, 95% CI 24.5–91.5%) for patients who received radiotherapy before and those who were radiotherapy naive, respectively (Supplementary Table S6).

Notably, for overall cohort or most subgroups, the efficacy seemed to be superior in the 180 mg group than that in the 120 mg group (Supplementary Table S6). Based on the efficacy results and comparable safety between the 120 mg group and the 180 mg group, once-daily 180 mg in continuous 21-day cycles with a 7 day lead-in at the dose of 60 mg was identified as the recommended phase 2 dose.

### Pharmacokinetics

PK data were derived from 53 patients in the dose-escalation phase. After once-daily dose of WX-0593 ranging from 30 to 300 mg, AUC_0–24_ of WX-0593 increased in an approximately dose-proportion manner, while *C*_max_ and AUC_0–∞_ did not have linear relation with dose groups.

The mean concentration–time profiles of WX-0593 over single dose at d1 and over multiple-dose at d21 by different dose groups are shown in Supplementary Fig. S2. The accumulation of WX-0593 exposure remained similar over different dose groups (e.g., AUC_0–24_: 1.16-fold to 3.09-fold). Detailed data on PK of WX-0593 are shown in Table 4.

**Table 4:** Summary of pharmacokinetic parameters of WX-0593 after single-dosing and multiple-dosing phase among different dose groups^a^

Single-dosing groups (*N* = 50)	AUC_0-t_ (ng·h/mL)	AUC_0–∞_ (ng·h/mL)	*C*_max_ (ng/mL)	*T*_max_ (h)	*T*_1/2z_ (h)	MRT_0-∞_ (h)
30 mg (*n* = 3)	1309.51 (498.25)	1707.99 (480.68)	104.77 (40.97)	2.322 (1.51)	15.46 (1.66)	20.67 (3.10)
60 mg (*n* = 3)	3645.57 (1530.96)	4060.20 (1666.54)	305.00 (153.41)	1.328 (0.582)	25.70 (3.33)	28.30 (4.91)
90 mg (*n* = 8)	5431.61 (2795.11)	6212.99 (3084.50)	474.40 (382.92)	1.685 (1.79)	21.87 (3.02)	23.98 (3.42)
120 mg (*n* = 11)	7776.79 (3298.75)	8117.50 (3626.34)	570.91 (324.25)	1.808 (1.31)	22.25 (4.42)	25.64 (5.09)
180 mg (*n* = 12)	6618.29 (4058.44)	7324.71 (4419.88)	401.17 (171.86)	2.288 (1.48)	23.87 (4.27)	27.88 (5.34)
240 mg (*n* = 10)	15507.22 (11498.49)	16896.82 (12379.63)	1000.70 (605.19)	2.497 (0.70)	22.90 (4.29)	25.04 (4.16)
300 mg (*n* = 3)	14949.85 (5229.20)	17094.41 (5554.90)	904.67 (255.82)	2.711 (1.15)	27.52 (5.75)	32.41 (6.82)
Multiple-dosing groups (*N* = 53)	AUC_0–24_ (ng·h/mL)	*C*_ssmax_ (ng/mL)	*T*_max_ (h)	*C*_ssmin_ (ng/mL)	Racc_AUC (%)	Racc_C_max_ (%)
30 mg (*n* = 3)	1147.89 (485.52)	95.13 (54.02)	4.333 (1.15)	25.00 (8.04)	116.256 (32.91)	89.141 (35.00)
60 mg (*n* = 3)	4964.51 (1896.51)	419.00 (128.24)	4.011 (0.98)	117.87 (59.10)	203.993 (32.03)	150.689 (39.79)
90 mg (*n* = 8)	4393.33 (1283.44)	324.38 (109.58)	2.871 (1.36)	103.65 (29.27)	149.978 (88.53)	117.082 (97.12)
120 mg (*n* = 11)	6124.49 (1748.87)	479.20 (151.70)	2.788 (1.75)	139.24 (34.22)	130.809 (36.42)	111.519 (44.39)
180 mg (*n* = 13)	7209.63 (2929.61)	529.38 (203.22)	2.791 (18.01)	164.10 (77.76)	174.265 (35.49)	138.238 (26.54)
240 mg (*n* = 12)	12712.31 (4099.43)	960.50 (234.47)	3.275 (1.61)	236.00 (56.80)	160.210 (77.95)	127.749 (58.74)
300 mg (*n* = 2)	23375.85 (10126.09)	1685.00 (657.61)	1.242 (1.05)	623.50 (402.34)	309.507 (151.00)	223.005 (88.95)

*AUC*_*0–24*_ area under the plasma concentration–time curve from time 0 to last time of quantifiable concentration, *AUC*_*0–∞*_ area under the plasma concentration–time curve from time 0 extrapolated to infinite time, *C*_*max*_ peak concentration, *T*_*max*_ time to reach maximum concentration, *T*_*1/2z*_ elimination half-life calculated by Rcsenblueth methods, *MRT*_*0–∞*_ mean residence time from time 0 extrapolated to infinite time, *AUC*_*0–24*_ area under the plasma concentration–time curve over a 24-h dosing interval, *C*_*ssmax*_ peak concentration at stable status, *C*_*ssmin*_ trough concentration at stable status, *Raac_AUC* accumulation ratio for AUC (calculated as AUC_0–24_ [stable status]/AUC_0–24_ [D1 of single dose]); *Raac_C*_*max*_ accumulation ratio for *C*_max_ (calculated as *C*_max_ [stable status]/*C*_max_ [D1 of single dose])

^a^Data are displayed as mean (standard deviation)

## Discussion

To the best of our knowledge, this was the first reported FIH study evaluating an ALK and ROS1 TKI in Chinese patients with *ALK*- or *ROS1*-rearranged NSCLC. This phase I study assessed the safety, efficacy, and PK of WX-0593 in Chinese patients with *ALK*-rearranged or *ROS1*-rearranged advanced NSCLC. WX-0593 showed a manageable safety profile with predominantly grade 1 or 2 AEs. Evidence of promising antitumor activity was observed in both *ALK*- and *ROS1*-rearranged patients. Based on the safety, PK, and efficacy results between the 120 mg group and the 180 mg group, once-daily 180 mg in continuous 21-day cycles with a 7 day lead-in at the dose of 60 mg was identified as the recommended phase 2 dose.

In the dose-escalation phase, one patient in the 300 mg dosing cohort experienced grade 3 QT prolongation and grade 2 chronic heart failure during the first cycle of WX-0593 treatment, which were defined as DLTs. Considering that the AEs might be more serious with dose escalation and that the promising activity had already been observed at the dose levels of 120 mg and 180 mg, the decision was made not to enroll 3 additional patients at the 300 mg dose. Therefore, MTD could not be identified. WX-0593 was generally well tolerated, and most AEs were grade 1–2. Only 23% (35/152) of patients experienced TRAEs of grade ≥3.

Overall, the safety of WX-0593 in the current study was similar but somewhat not identical to that observed in other ALK TKIs. Consistent with other ALK TKIs, WX-0593 led to liver-function abnormalities, most commonly elevated ALT or AST level, which was manageable and could be resolved through dose interruption or reduction.^6,17^ However, the frequency of grade ≥3 ALT or AST elevation related to the study drug was higher in ceritinib than that reported for WX-0593.^18,19^ Diarrhea occurred in 48, 41, and 75% of patients treated with crizotinib, brigatinib, and ceritinib, respectively,^6,18,20^ whereas only 14% (22/152) of patients who received WX-0593 experienced diarrhea. Other gastrointestinal disorders, such as nausea and vomiting, mostly grade 1–2, were less frequently observed in this study than that reported with crizotinib.^6^ Similar to lorlatinib but distinct to other ALK TKIs, hypercholesterolemia and hypertriglyceridemia were also commonly seen with WX-0593, but the incidence of both were lower compared with those reported for lorlatinib.^21^ It remains unclear by which mechanism WX-0593 led to dysregulation of lipid metabolism. However, these AEs could be manageable with medication, such as statin or fibrate treatment. Additionally, rash, the most common TRAE for ensartinib, occurred in 56% of patients treated with ensartinib,^22,23^ whereas it was not common with WX-0593 (only 11%). Furthermore, dose reduction due to TEAEs occurred in only 3% (4/152) of patients in this study, compared with 16% for alectinib, 62% for ceritinib, and 14% for brigatinib.^13,20,24^ Taken together, WX-0593 had a good safety profile in patients with *ALK*- or *ROS1*-rearranged NSCLC.

Notably, 2 deaths (1 due to infectious pneumonia and 1 due to respiratory failure, all within 48 h of WX-0593 treatment) occurred among patients who received the regimen of once-daily 120 mg without a 7-day lead-in at the dose of 60 mg in the dose-expansion phase, which were considered as possibly related to WX-0593. In the previous phase 1/2 trial of brigatinib, a subset of patients experienced early-onset pulmonary events that occurred within 7 days (usually within 24–48 h) of treatment, whereas none of patients who received once-daily 180 mg with a 7-day lead-in at the dose of 90 mg had such event.^20^ Therefore, referring to the study design of brigatinib, the amended protocol stipulated that a 7-day lead-in at the dose of 60 mg was administered in the dose-expansion phase of our study. Consistent with the findings of the above trial of brigatinib, no early-onset pulmonary events re-occurred among patients receiving the dose with a 7-day lead-in at the dose of 60 mg in our study, which indicated a 7-day lead-in at a lower dose might have potential to reduce the incidence of early-onset pulmonary events.

WX-0593 demonstrated promising efficacy in advanced NSCLC patients with *ALK* rearrangement. Overall, 82.3% of patients enrolled in this study had received prior one or more lines of anticancer therapy. For *ALK*-rearranged patients, 29 of the 46 (63.0%) and 53 of the 91 (58.2%) patients in the dose-escalation and dose-expansion phases, respectively, achieved objective response, which was comparable with those observed in other second-generation ALK inhibitors, such as ceritinib (58%) and ensartinib (60%).^18,23^ In the FIH phase I study of crizotinib, which involved 82 patients with *ALK*-rearranged advanced NSCLC who never received ALK inhibitor, the ORR was 57%, and PFS at 6 months was estimated at 72%.^6^ By contrast, the ORRs for patients with *ALK*-rearranged NSCLC who never received other ALK inhibitors previously were 81.0% (17/21) in the dose-escalation phase and 76.3% (29/38) in the dose-expansion phase of this study, which were numerically higher compared to the above crizotinib study.

Also, WX-0593 demonstrated clinical activity for patients who had progressed on crizotinib. In NSCLC patients with *ALK* rearrangement who were treated with prior crizotinib, several second-generation ALK TKIs showed encouraging efficacy, with ORRs of 44–56%, and median PFS of 6.9–13.2 months.^20,22,24,25^ In the dose expansion of this study, the confirmed ORR of 45.7% and median PFS of 6.9 months with WX-0593 for *ALK*-rearranged patients treated with crizotinib as the only ALK TKI were comparable to that of other *ALK* inhibitors, indicating the meaningful clinical activity of WX-0593 in the post-crizotinib setting.

The kinase domains of *ALK* and *ROS1* exhibit substantial homology,^26^ thus several ALK inhibitors also have demonstrated efficacy in patients with *ROS1* rearrangements. In a phase I study involving 50 NSCLC patients with *ROS1* rearrangement treated with crizotinib, the ORR was 72% and median PFS was 19.2 months.^8^ Second-generation ALK TKI, such as ceritinib, has also shown tumor responses in *ROS1*-rearranged patients.^27^ Lorlatinib yielded an ORR of 62% in ALK TKI-naive patients with ROS1 rearrangement and 35% in patients who received crizotinib as the only TKI.^16^ Despite this, crizotinib and entrectinib are the only two ROS1 TKIs approved by FDA for *ROS1*-rearranged NSCLC to date. In this study, WX-0593 was found to have potent antitumor activity for advanced NSCLC patients with *ROS1* rearrangement. The ORRs for crizotinib-naive patients were 42.9 and 80.0%, and median PFS were 3.65 months and not reached, respectively, for the dose-escalation and dose-expansion phases. The small number of patients (seven in dose-escalation phase and five in expansion phase) might explain the large difference between ORRs observed in the two phases. A further prospective phase 2 study (NCT04641754) with larger sample size is underway to investigate WX-0593 in this patient population.

The CNS progression has been reported to occur frequently during treatment with crizotinib. Several second- and third-generation ALK TKIs showed intracranial antitumor activity.^20,21,24,28^ Consistent with these findings, WX-0593 also displayed an evidence of promising intracranial efficacy, demonstrating an intracranial ORR of 46.7% (7/15) for patients with baseline measurable CNS metastasis in the dose-expansion phase. Significantly, both ALK TKI-naive patients and those previously given crizotinib as the only TKI yielded intracranial response rates of 50%, suggesting the potential clinical benefit of WX-0593 for patients with CNS metastases, regardless of prior crizotinib exposure. Moreover, it is worth noting that previous CNS radiotherapy may influence the result. This study revealed that the intracranial response with WX-0593 in patients with history of CNS radiotherapy was numerically inferior than that in those without prior CNS radiotherapy, which was in correspondence with a previous report of brigatinib.^20^ Although caution must be paid when interpreting these data due to the small number of patients, it indicated that WX-0593 might penetrate the blood–brain barrier to efficaciously control brain lesions.

To date, four ALK TKIs including crizotinib, ceritinib, alectinib and ensartinib have been on the market in mainland China. Despite this, the profiles of safety and efficacy for these agents are different, and the relatively high price may hinder them from clinical application. Alternative next-generation ALK inhibitors, which have potent therapeutic effects against ALK rearrangement genes and/or the common resistant mutations, are still needed. Given the favorable safety profile and promising efficacy, WX-0593 may potentially provide a new therapeutic option for Chinese advanced NSCLC patients with *ALK* or *ROS1* rearrangement, thus contributing to reducing the treatment costs and increasing drug accessibility.

Several limitations of this study need to be acknowledged. This study is limited by the short follow-up period and a lack of efficacy assessment by the Independent Review Committee. Additionally, since this study was not a head-to-head comparative trial, caution must be paid when comparing with other ALK inhibitor trials. Another limitation is that the molecular analysis was not conducted to further explore the association between specific targets with clinical efficacy in this study. More research is needed to explore which genetic alterations could predict response or resistance to WX-0593. Furthermore, considering that the number of NSCLC patients with *ROS1* rearrangements or brain metastases enrolled was relatively small, study findings need to be interpreted cautiously. Further prospective studies with larger sample sizes are warranted to investigate WX-0593 in this patient population.

## Conclusions

In conclusion, WX-0593 showed a favorable safety profile and promising antitumor activity in advanced NSCLC patients with *ALK* or *ROS1* rearrangement. The recommended phase 2 dose was determined to be once-daily 180 mg in continuous 21-day cycles with a 7-day lead-in at the dose of 60 mg. Based on the findings of this study, a pivotal phase 2 study (NCT04641754) is ongoing to further inform the therapeutic potential of WX-0593 in advanced NSCLC patients with *ALK* or *ROS1* rearrangements. Besides, a phase 3 randomized study (NCT04632758) of WX-0593 compared with crizotinib for advanced *ALK*-rearranged NSCLC in the first-line setting is ongoing.

## Methods

### Study design

This FIH, single arm, multicenter, phase 1 study was conducted in advanced NSCLC patients with *ALK* or *ROS1* rearrangement, composed of the dose-escalation and dose-expansion phases. The dose-escalation and dose-expansion phases were conducted at 4 and 38 centers in mainland China, respectively (Supplementary Table S1), and were done in compliance with the Declaration of Helsinki and International Council for Harmonisation guidelines for Good Clinical Practice. The study protocol and amendments were approved by the independent ethics committee at every participating site. Written informed consent was provided by all patients before being screened for enrolment.

### Study population

Patients with pathologically confirmed diagnosis of advanced NSCLC harboring *ALK* or *ROS1* rearrangement, who progressed on standard treatment (e.g., resistant to *ALK* inhibitors or chemotherapy), or could not accept chemotherapy, or were intolerant with chemotherapy, were eligible for this study. *ALK* and *ROS1* rearrangements were established by immunohistochemistry, reverse transcription-polymerase chain reaction (PCR), amplification refractory mutation system-PCR, fluorescence in situ hybridization, or next-generation sequencing. Patients must have at least one measurable lesion as defined by Response Evaluation Criteria in Solid Tumors (RECIST) version 1.1. Other key eligibility criteria included an age of 18–70 years, an Eastern Cooperative Oncology Group performance status of 0 or 1, a life expectancy of at least 12 weeks, and adequate organ function, including adequate function of bone marrow (absolute neutrophil count ≥1.5 × 10^9^/L, platelets ≥100 × 10^9^/L, hemoglobin ≥90 g/L) and liver function (total bilirubin ≤1.5× upper limit of normal [ULN], ALT and AST ≤2.5× ULN [≤5.0× ULN for liver metastasis patients]). Patients with asymptomatic CNS metastases or symptomatic brain metastasis who had remained stable for >4 weeks after treatment were allowed to enroll.

Key exclusion criteria included leptomeningeal disease and clinically significant cardiovascular and cerebrovascular disease within 3 months before the first dosing of WX-0593. Patients who received other investigational drugs within 1 month or prior anticancer therapy within 2 weeks (*t*_1/2_ ≤ 3 days) or within 4 weeks (*t*_1/2_ > 3 days) were not permitted to enroll. Patients receiving prior crizotinib were permitted to receive WX-0593 treatment after 1 week from the last dose of crizotinib. The full list of eligibility and exclusion criteria is provided in the study protocol of Supplementary Materials.

### Procedures

In the dose-escalation phase, a standard 3 + 3 design was used to determine the MTD.^29^ Patients were enrolled into 7 dosing cohorts of WX-0593: 30, 60, 90, 120, 180, 240, and 300 mg. The starting dose for WX-0593 was 30 mg, which was selected on the basis of preclinical toxicology studies (data unpublished), according to the International Conference on Harmonisation Guidance for Industry S9 Nonclinical Evaluation for Anticancer Pharmaceuticals.^30^

Three patients were assigned to each dose level. In the dosing cohort of 30 mg, treatment included a single WX-0593 dose, followed by 7-day PK evaluation period, and subsequent daily oral dosing for continuous 21 days. In other dosing cohorts, WX-0593 was administered orally as a single dose in the 4-day PK run-in period, followed by dosing once per day in continuous 21-day cycles. The DLT observation period was the first cycle of WX-0593 treatment (the single-dose period and 21 days of multiple-dose period).

Considering that safety and efficacy in the dosing cohorts of 120 and 180 mg were found to be numerically superior than that in other dosing cohorts in dose-escalation phase, the two dose levels of 120 and 180 mg were selected for further evaluation in the dose-expansion phase. According to the protocol (version 2.4 and 2.5), patients initially received a continuous WX-0593 dose of 120 or 180 mg orally once daily and continued to be treated with the same dosing after 7-day observation. Due to the observation of pulmonary events occurring within 48 h of WX-0593 treatment, the amended protocol (version 3.0) stipulated that the WX-0593 dose of 120 or 180 mg was administered orally once per day in continuous 21-day cycles with a 7-day lead-in at the dose of 60 mg. Patients continued treatment until documented disease progression, unacceptable toxicity, withdrawal of consent, the start of a new anticancer treatment, death, loss of follow-up, or end of the study (whichever occurred first). Patients were permitted to continue treatment after progression if they were still experiencing clinical benefit, at the discretion of the investigator.

For grade ≥3 AEs (also including grade ≥2 cardiac dysfunction and renal dysfunction, etc.), dose interruption could occur. If the AE lessened to grade ≤1 (except for hyperlipidemia and transient abnormal liver enzymes, etc.) after supportive care within ≤14 days, WX-0593 treatment could be resumed at the same dose level or next-lowest dose level according to the investigator’s judgment. Patients who had any grade ≥3 AEs again after treatment resumption were discontinued from treatment with WX-0593 permanently.

Patients performed imaging evaluation at baseline, with computed tomography or magnetic resonance imaging of the chest, abdomen, pelvis, and brain. In the dose-escalation phase, the first efficacy evaluation was done after 3-week continuous administration of WX-0593 and every 6 weeks thereafter. In the dose-expansion phase, tumor response was assessed every 6 weeks until 48 weeks and every 12 weeks thereafter until documented disease progression. If responses (CR or PR) were observed, repeat assessments were required to confirm the responses 4 weeks after the initial evaluation. Tumor responses were assessed by local investigators according to RECIST 1.1. AEs were assessed in all patients throughout this study and graded per the National Cancer Institute Common Terminology Criteria for Adverse Events version 4.03.

In the dose-escalation phase, blood samples were collected at protocol-specified time points after single and multiple dose administration of WX-0593 in all dosing cohorts for assessment of PK parameters.

### Study endpoints

In the dose-escalation phase, the primary endpoints were MTD, DLT, and safety assessed by investigators and the subsequent recommended dose of WX-0593. Safety parameters included TEAEs, SAEs, and TRAEs. The key secondary endpoints were PK parameters and preliminary antitumor activity of WX-0593.

In the dose-expansion phase, the primary endpoint was ORR assessed by investigators. Other key secondary endpoints included PFS, DCR, time to progression (TTP), DOR, intracranial response, and safety. Additionally, overall survival (OS) was assessed as a prespecified exploratory endpoint.

ORR was defined as the proportion of patients who had best overall response, including CR or PR. PFS was calculated from the date of the first dose of WX-0593 to the date of documented disease progression or death (whichever occurred first). DCR was defined as the proportion of patients who had best overall response, including CR, PR, or SD, with a duration of at least 12 weeks. TTP was calculated from the date when the first dose of WX-0593 was given to first documented disease progression. DOR was calculated from the date when first documented CR or PR (subsequently confirmed) was observed to the date of first documented disease progression or death (whichever occurred first). OS was calculated from the date when the first dose of WX-0593 was administered to the date of death from any cause.

### Statistical analysis

There was no formal hypothesis testing in both dose-escalation and dose-expansion phases of this study. For the dose-escalation cohort, it was planned that 39–60 patients would be enrolled on the basis of PK evaluation. For the dose-expansion phase, two dosing cohorts were selected for further analysis, and a total of 60–140 patients were planned to be enrolled with at least 30 and up to 70 patients in each dosing cohort.

Safety analysis was performed in the SS, which was comprised of all patients treated with one dose of WX-0593 and with safety data recorded. Efficacy analysis was performed in the FAS, which was defined as all patients treated with at least one dose of WX-0593. The PK analysis set included all patients treated with at least one dose of WX-0593 and with at least one evaluable PK sample. Additionally, we did subgroup analyses of efficacy in patients by the status of gene (*ALK* or *ROS1* rearrangement), previous ALK TKI treatment (ALK TKI naive, prior crizotinib as the only ALK TKI, prior crizotinib and other ALK TKIs, or other), and brain metastases at baseline (yes or no).

Descriptive statistics were carried out to summarize patients’ baseline characteristics and safety. The response rates with exact binomial 95% CIs were calculated using the Clopper–Pearson method. All time-to-event data (PFS, TTP, DOR, and OS) and their corresponding 95% CIs were estimated by Kaplan–Meier methods. PK parameter analyses were done with Phoenix WinNonlin version 8.0. All other statistical analyses were conducted using SAS version 9.4.

This study was registered with ClinicalTrials.gov, number NCT03389815.

## Supplementary information

Supplementary material

Protocol

## Data Availability

The data that support the findings of this study can be accessed from the corresponding author upon reasonable request.
