# Supplementary material for: Safety and activity of WX-0593 (Iruplinalkib) in patients with ALK- or ROS1-rearranged advanced non-small cell lung cancer: a phase 1 dose-escalation and dose-expansion trial
Source: Signal Transduct Target Ther. 2022 Jan 28;7:25. doi: 10.1038/s41392-021-00841-8 (PMC8795197; doi:10.1038/s41392-021-00841-8)
Supplement: Supplementary file 1 — Supplementary material [file 41392_2021_841_MOESM1_ESM.docx]

**Supplementary Material**

**Safety and activity of WX-0593 in *ALK*-rearranged or *ROS1*-rearranged advanced non-small cell lung cancer: a phase 1 dose escalation and expansion trial**

Yuankai Shi, Jian Fang, Xuezhi Hao , Shucai Zhang, Yunpeng Liu, Lin Wang, Jianhua Chen, Yi Hu, Xiaosheng Hang, Juan Li, Chunling Liu, Yiping Zhang, Zhehai Wang, Yanping Hu, Kangsheng Gu, Jian'an Huang, Liangming Zhang, Jinlu Shan, MD, Weiwei Ouyang, Yanqiu Zhao, Wu Zhuang, Yan Yu, Jun Zhao, Helong Zhang, Pei Lu, Weidong Li, Meimei Si, Mingjing Ge, Huaize Geng

Table of Contents

[Supplementary Preclinical data 2](#_Toc80639844)

[Table S1. Patient enrollment and participating sites 7](#_Toc80639845)

[Table S2. Overall Summary of Adverse Events by Dose in safety set (n=152) 9](#_Toc80639846)

[Table S3. Treatment related adverse events reported in ≥ 10% of patients by dose and severity 10](#_Toc80639847)

[Table S4. Summary of Efficacy by dose in escalation study phase 11](#_Toc80639848)

[Table S5. Summary of objective response rate by subgroup in dose escalation phase (n=54) 12](#_Toc80639849)

[Table S6. Summary of objective response rate by subgroup in dose expansion phase (n=99) 14](#_Toc80639850)

[Figure S1. The molecular structure of WX-0593 16](#_Toc80639851)

[Figure S2. (a) Mean plasma WX-0593 concentration-time curve of different dose groups for single-dosing in dose escalation phase (PK set). (b) Mean plasma WX-0593 concentration-time curve of different dose groups on D21 for multiple-dosing in dose escalation phase (PK set) 17](#_Toc80639852)

[Figure S3. (a) Duration of response of patients in dose-expansion phase (n=56); (b) Progression-free survival of patients in dose-expansion phase (n=99) 18](#_Toc80639853)

# Supplementary Preclinical data

**1. The preclinical data of WX-0593 against *ALK***

**1.1 Results**

WX-0593 has superior inhibitory activity against the ALK mutants resistant to other ALK inhibitors at therapeutic concentration when administrated 180 mg, once daily (the free Cavg,ss is 127 nM). Also, WX-0593 has inhibitory activity on ALK-G1202R mutants which is gatekeeper mutation resistant to all other ALKi except lorlatinib.

**Figure 1.** The inhibitory activity of ALKis on growth of Ba/F3 transfected with ALK wild type and different ALK resistant mutants kinase

**Table 1.** The GI_50_ of ALKis on growth of Ba/F3 transfected with ALK wild type and different ALK resistant mutants kinase

| ALK Mutants | IC_50_ (nM) | | | | | | |
| --- | --- | --- | --- | --- | --- | --- | --- |
|  | WX-0593 | Crizotinib | Ensartinib | Ceritinib | Alectinib | Brigatinib | Lorlatinib |
| ALK-WT | 21 | 64 | 14 | 13 | 43 | 25 | 4 |
| ALK-L1196M | 36 | 478 | 98 | 33 | 205 | 31 | 54 |
| ALK-F1174L | 36 | 82 | 29 | 21 | 32 | 31 | 4 |
| ALK-C1156Y | 9 | 353 | 17 | 87 | 10 | 39 | 4 |
| ALK-G1202R | 96 | 1241 | 409 | 502 | 1000 | 340 | 26 |
| ALK-G1202del | 930 | 573 | 932 | 925 | 1000 | 1000 | 215 |
| ALK-F1174C | 36 | 303 | 62 | 95 | 41 | 97 | 11 |
| ALK-G1269A | 36 | 553 | 212 | 16 | 95 | 31 | 35 |
| ALK-I1171S | 32 | 441 | 39 | 97 | 229 | 93 | 33 |
| ALK-T1151Tins | 12 | 473 | 42 | 96 | 26 | 35 | 7 |
| ALK-F1174V | 22.3 | 195.4 | 54.5 | 53.7 | 28.7 | 47.8 | 16.1 |
| ALK-F1174C | 29.3 | 195.5 | 62.2 | 79.5 | 27.2 | 51.6 | 15.9 |
| ALK-V1180L | 8.4 | 127.6 | 16.6 | 15.6 | 469 | 10.4 | 5.2 |
| ALK-L1152R | 4.6 | 448.6 | 71.4 | 115.7 | 13.4 | 10.4 | 17.4 |
| ALK-L1152P | 1.5 | 227.8 | 25.7 | 71.5 | 4.6 | 1.5 | 4.6 |
| ALK-I1171S | 21.4 | 326.8 | 41.5 | 45.8 | 151.2 | 40 | 52.8 |
| ALK-I1171T | 11.3 | 213.2 | 28 | 27.8 | 49.6 | 18.9 | 19 |
| ALK-I1171N | 17.9 | 204.7 | 18.4 | 41.8 | 49.6 | 35 | 41.1 |

**1.2 Material**

The Ba/F3 cell line transfected with different ALK mutants were constructed by Kyinno Biotechnology Co., Ltd.; 96-Well Flat Clear Bottom Black Polystyrene TC-Treated Microplate (Cat#165306, Thermo®); CellTiter-Glo^®^ Luminescent Cell Viability Assay (Cat. No.: G7573, Promega. Store at -20°C); FBS(GBICO, Cat#10099-141); RPMI-1640(Hyclone，Cat# SH30809.01).

**1.3 Method**

Harvest cells during the logarithmic growth period and count cells using blood counting chamber. Adjust cell concentration with culture medium. Add 90µl cell suspensions to two 96-well plates to the final cell density as described before. Incubate the plate overnight in humidified incubator at 37℃ with 5% CO_2_. Dispense 10µl drug solution (10×) to each well (triplicate for each concentration), and the DMSO final concentration in culture medium is 0.1% [v/v]. Culture the plates for 3 days. On Day 3, Read luminescent using Multi Label Reader. The software of GraphPad Prism was used to calculate IC_50_. The graphical curves were fitted using a nonlinear regression model with a sigmoidal dose response.

**2. The preclinical data of WX-0593 against *ROS1***

**2.1 Results**

WX-0593 has superior inhibitory activity against the *ROS1* wild type and mutants resistant to crizotinib at therapeutic concentration when administrated 180 mg, once daily (the free Cavg,ss is 127 nM), except for *ROS1*-G2032R and *ROS1*-L1951R mutants. WX-0593 may be a potent therapy for *ROS1* positive NSCLC patients who are resistant and not tolerated to crizotinib.

**Figure 1.** The inhibitory activity of ALKis on growth of Ba/F3 transfected with ROS1 wild type and different ROS1 resistant mutants

**Table 1.** The GI_50_ of ALKis on growth of Ba/F3 transfected with ROS1 wild type and different ROS1 resistant mutants

| ROS1 Mutant | IC_50_ (nM) | | | | | | |
| --- | --- | --- | --- | --- | --- | --- | --- |
|  | WX-0593 | Crizotinib | Ensartinib | Ceritinib | Alectinib | Brigatinib | Lorlatinib |
| Ba/F3 SLC34A2-ROS1 | 11 | 8 | 49 | 14 | >1000 | 22 | <1 |
| Ba/F3 SLC34A2-ROS1-G2032R | 584 | 982 | 1635 | 1100 | >1000 | 846 | 197 |
| Ba/F3 SLC34A2-ROS1-L2026M | 12 | 162 | 278 | 54 | >1000 | 48 | <1 |
| Ba/F3 CD74-ROS1-S1986Y | 15.9 | 22.2 | 188.7 | 32.5 | 10000 | 12.5 | 1.5 |
| Ba/F3 CD74-ROS1-S1986F | 22.4 | 29.9 | 191.8 | 49.6 | 10000 | 20.2 | 1.1 |
| Ba/F3 CD74-ROS1-D2033N | 33.8 | 89.9 | 195.2 | 159.2 | 5068 | 36.5 | 1.7 |
| Ba/F3 CD74-ROS1-L1951R | 625.8 | 179.3 | 970.4 | 641.6 | 5409 | 775.3 | 5.1 |

**2.2 Material**

The Ba/F3 cell line transfected with different ALK mutants were constructed by Kyinno Biotechnology Co., Ltd. and Precedo Pharmaceuticals Co., Ltd.; 96-Well Flat Clear Bottom Black Polystyrene TC-Treated Microplate (Cat#165306, Thermo®); CellTiter-Glo^®^ Luminescent Cell Viability Assay (Cat. No.: G7573, Promega. Store at -20°C); FBS(GBICO, Cat#10099-141); RPMI-1640(Hyclone，Cat# SH30809.01).

**2.3 Method**

Harvest cells during the logarithmic growth period and count cells using blood counting chamber. Adjust cell concentration with culture medium. Add 90µl cell suspensions to two 96-well plates to the final cell density as described before. Incubate the plate overnight in humidified incubator at 37℃ with 5% CO_2_. Dispense 10µl drug solution (10×) to each well (triplicate for each concentration), and the DMSO final concentration in culture medium is 0.1% [v/v]. Culture the plates for 3 days.On Day 3, Read luminescent using Multi Label Reader. The software of GraphPad Prism was used to calculate IC_50_. The graphical curves were fitted using a nonlinear regression model with a sigmoidal dose response.

# Table S1. Patient enrollment and participating sites

| **Site number** | **Site** | **Location** | **Principal Investigator** | **Number of patients enrolled** |
| --- | --- | --- | --- | --- |
| 02 | Beijing Cancer Hospital | Beijing, China | Jian Fang | 24 |
| 01 | National Cancer Center/National Clinical Research Center for Cancer/Cancer Hospital, Chinese Academy of Medical Sciences & Peking Union Medical College, Beijing Key Laboratory of Clinical Study on Anticancer Molecular Targeted Drugs | Beijing, China | Yuankai Shi | 21 |
| 03 | Beijing Chest Hospital, Capital Medical University | Beijing, China | Shucai Zhang | 17 |
| 04 | The First Hospital of China Medical University | Shenyang, China | Yunpeng Liu | 16 |
| 32 | Hunan Cancer Hospital | Changsha, China | Jianhua Chen | 5 |
| 11 | Chinese PLA General Hospital | Beijing, China | Yi Hu | 5 |
| 19 | Affiliated Hospital of Jiangnan University | Wuxi, China | Xiaosheng Hang | 5 |
| 34 | Sichuan Cancer Hospital | Chengdu, China | Juan Li | 5 |
| 13 | Affiliated Tumor Hospital of Xinjiang medical University | Xinjiang, China | Chunling Liu | 4 |
| 20 | Zhejiang Cancer Hospital | Hangzhou, China | Yiping Zhang | 4 |
| 23 | Shandong Cancer Hospital and Institute, Shandong First Medical University and Shandong Academy of Medical Sciences | Jinan, China | Zhehai Wang | 4 |
| 45 | Hubei Cancer Hospital | Wuhan, China | Yanping Hu | 4 |
| 15 | The First Affiliated Hospital of Anhui Medical University | Hefei, China | Kangsheng Gu | 3 |
| 18 | The First Affiliated Hospital of Soochow University | Suzhou, China | Jianan Huang | 3 |
| 26 | Yantai Yuhuangding Hospital | Yantai, China | Liangming Zhang | 3 |
| 27 | Henan Cancer Hospital | Zhengzhou, China | Yanqiu Zhao | 3 |
| 33 | Army Medical Center of People’s Liberation Army | Chongqing, China | Jinlu Shan | 3 |
| 36 | Guizhou Cancer Hospital | Guiyang, China | Weiwei Ouyang | 3 |
| 40 | Fujian Provincial Cancer Hospital | Fuzhou, China | Wu Zhuang | 3 |
| 5 | Harbin Medical University Cancer Hospital | Harbin, China | Yan Yu | 2 |
| 10 | Beijing Cancer Hospital | Beijing, China | Jun Zhao | 2 |
| 29 | Tangdu Hospital of the Fourth Military Medical University | Xi'an, China | Helong Zhang | 2 |
| 38 | Liuzhou People's Hospital | Liuzhou, China | Pei Lu | 2 |
| 42 | Cancer Center of Guangzhou Medical University | Guangzhou, China | Weidong Li | 2 |
| 7 | Jilin Province Cancer Hospital | Changchun, China | Ying Cheng | 1 |
| 14 | Xuzhou Central Hospital | Xuzhou, China | Sanyuan Sun | 1 |
| 16 | The First Affiliated Hospital of Anhui Medical University | Anhui, China | Rongyu Liu | 1 |
| 17 | Anhui Province Cancer Hospital | Hefei, China | Changlu Hu | 1 |
| 22 | Qilu Hospital of Shandong University | Jinan, China | Xiuwen Wang | 1 |
| 25 | The Affiliated Hospital of Qingdao University | Qingdao, China | Xiaochun Zhang | 1 |
| 28 | The First Affiliated Hospital of Xi'an Jiaotong University | Xi'an, China | Yu Yao | 1 |
| 46 | Anhui Province Cancer Hospital | Hefei, China | Zhihong Zhang | 1 |
| 6 | First Hospital of Jilin University | Changchun, Jilin | Jiuwei Cui | 0 |
| 24 | The 960th Hospital of the PLA Joint Logistics Support Force | Jinan, China | Bocheng Wang | 0 |
| 30 | Hospital General Hospital of Lanzhou Military Region of of PLA | Lanzhou, China | Baihong Zhang | 0 |
| 35 | Affiliated Hospital of Zunyi Medical University | Guiyang, China | Hu Ma | 0 |
| 41 | The First Affiliated Hospital of Xiamen University | Xiamen, China | Feng Ye | 0 |
| 44 | Yunnan Cancer Hospital | Yunnan, China | Runxiang Yang | 0 |

# Table S2. Overall Summary of Adverse Events by Dose in safety set (n=152)

|  | **30mg**  **(n=3)** | **60mg**  **(n=3)** | **90mg**  **(n=8)** | **120mg**  **(n=61)** | **180mg**  **(n=61)** | **240mg**  **(n=13)** | **300mg**  **(n=3)** | **Total**  **(n=152)** |
| --- | --- | --- | --- | --- | --- | --- | --- | --- |
| Total | 3(100%) | 3(100%) | 8(100%) | 58(95%) | 60(98%) | 13(100%) | 3(100%) | 148(97%) |
| treatment-related | 2(67%) | 2(67%) | 8(100%) | 54(89%) | 58(95%) | 13(100%) | 3(100%) | 140(92%) |
| ≥grade 3 adverse event | 1(33%) | 1(33%) | 4(50%) | 20(33%) | 21(34%) | 5(38%) | 1(33%) | 53(35%) |
| treatment-related | 0 | 0 | 2(25%) | 13(21%) | 14(23%) | 5(38%) | 1(33%) | 35(23%) |
| led to dose reduction | 0 | 0 | 0 | 1(2%) | 2(3%) | 1(8%) | 0 | 4(3%) |
| treatment-related | 0 | 0 | 0 | 1(21%) | 2(3%) | 1(8%) | 0 | 4(3%) |
| led to dose interruption | 0 | 0 | 0 | 13(21%) | 14(23%) | 3(23%) | 0 | 30(20%) |
| treatment-related | 0 | 0 | 0 | 13(21%) | 11(18%) | 3(23%) | 0 | 27(18%) |
| led to treatment termination | 0 | 0 | 1(13%) | 5(8%) | 4(7%) | 0 | 1(33%) | 11(7%) |
| treatment-related | 0 | 0 | 0 | 4(7%) | 3(5%) | 0 | 1(33%) | 8(5%) |
| Serious adverse event | 1(33%) | 0 | 1(13%) | 11(18%) | 11(18%) | 0 | 1(33%) | 25(16%) |
| treatment-related | 0 | 0 | 0 | 4(7%) | 5(8%) | 0 | 1(33%) | 10(7%) |
| Discontinuation due to adverse event | 0 | 0 | 1(13%) | 5(8%) | 4(7%) | 0 | 1(33%) | 11(7%) |
| treatment-related | 0 | 0 | 0 | 4(7%) | 3(5%) | 0 | 1(33%) | 8(5%) |
| Death during treatment | 0 | 0 | 1(13%) | 4(7%) | 2(3%) | 0 | 0 | 7(5%) |
| treatment-related | 0 | 0 | 0 | 2(3%) | 0 | 0 | 0 | 2(1%) |
| not related with disease progression | 0 | 0 | 1(13%) | 4(7%) | 1(2%) | 0 | 0 | 6(4%) |
| related with disease progression | 0 | 0 | 0 | 0 | 1(2%) | 0 | 0 | 1(1%) |

# Table S3. Treatment related adverse events reported in ≥ 10% of patients by dose and severity

| Treatment-related  adverse event | **30mg**  **(n=3)** | | **60mg**  **(n=3)** | | **90mg**  **(n=8)** | | **120mg**  **(n=61)** | | **180mg**  **(n=61)** | | **240mg**  **(n=13)** | | **300mg**  **(n=3)** | | **Total**  **(n=152)** | | |
| --- | --- | --- | --- | --- | --- | --- | --- | --- | --- | --- | --- | --- | --- | --- | --- | --- | --- |
|  | grade 1-2 | ≥grade 3 | grade 1-2 | ≥grade 3 | grade 1-2 | ≥grade 3 | grade 1-2 | ≥grade 3 | grade 1-2 | ≥grade 3 | grade 1-2 | ≥grade 3 | grade 1-2 | ≥grade 3 | All grade | grade 1-2 | ≥grade 3 |
| AST elevated | 0 | 0 | 0 | 0 | 3(38%) | 0 | 15(25%) | 2(3%) | 18(30%) | 2(3%) | 5(38%) | 1(8%) | 0 | 0 | 46(30%) | 41(27%) | 5(3%) |
| ALT elevated | 0 | 0 | 0 | 0 | 3(38%) | 0 | 15(25%) | 3(5%) | 16(26%) | 2(3%) | 4(31%) | 1(8%) | 0 | 0 | 44(29%) | 38(25%) | 6(4%) |
| nausea | 0 | 0 | 2(67%) | 0 | 3(38%) | 0 | 12(20%) | 0 | 17(28%) | 0 | 8(62%) | 0 | 1(33%) | 0 | 43(28%) | 43(28%) | 0 |
| hypercholesterolemia | 0 | 0 | 0 | 0 | 2(25%) | 0 | 13(21%) | 1(2%) | 14(23%) | 0 | 8(62%) | 0 | 2(67%) | 0 | 40(27%) | 39(26%) | 1(1%) |
| vomiting | 1(33%) | 0 | 1(33%) | 0 | 1(13%) | 0 | 10(16%) | 0 | 15(25%) | 0 | 7(54%) | 0 | 2(67%) | 0 | 37(24%) | 37(24%) | 0 |
| hypertriglyceridemia | 0 | 0 | 0 | 0 | 2(25%) | 0 | 11(18%) | 2(3%) | 8(13%) | 2(3%) | 6(46%) | 0 | 1(33%) | 0 | 42(21%) | 28(18%) | 4(3%) |
| hypertension | 0 | 0 | 1(33%) | 0 | 0 | 2(25%) | 5(8%) | 1(2%) | 6(10%) | 2(3%) | 5(38%) | 3(23%) | 1(33%) | 0 | 26(17%) | 18(12%) | 8(5%) |
| CPK elevated | 0 | 0 | 0 | 0 | 1(13%) | 0 | 6(10%) | 0 | 16(26%) | 0 | 1(8%) | 0 | 1(33%) | 0 | 25(16%) | 25(16%) | 0 |
| diarrhea | 0 | 0 | 0 | 0 | 0 | 0 | 3(5%) | 0 | 10(16%) | 1(2%) | 6(46%) | 0 | 2(67%) | 0 | 22(15%) | 21(14%) | 1(1%) |
| rash | 0 | 0 | 0 | 0 | 1(13%) | 0 | 4(7%) | 0 | 7(11%) | 1(2%) | 3(23%) | 0 | 0 | 0 | 16(11%) | 15(10%) | 1(1%) |
| γ-GGT elevated | 0 | 0 | 0 | 0 | 1(13%) | 0 | 5(8%) | 2(3%) | 7(11%) | 1(2%) | 1(8%) | 1(8%) | 0 | 0 | 14(10%) | 10(7%) | 4(3%) |
| hyperlipidemia | 1(33%) | 0 | 1(33%) | 0 | 3(38%) | 0 | 3(5%) | 0 | 6(10%) | 0 | 0 | 0 | 1(33%) | 0 | 15(10%) | 15(10%) | 0 |

Abbreviations: AST, aspartate transaminase; ALT, alamine aminotransferase; CPK, creatine phosphokinase; γ-GGT, γ-glutamyl transpeptidase.

# Table S4. Summary of Efficacy by dose in escalation study phase

|  | **30mg**  **(n=3)** | **60mg**  **(n=3)** | **90mg**  **(n=8)** | **120mg**  **(n=11)** | **180mg**  **(n=13)** | **240mg**  **(n=13)** | **300mg**  **(n=3)** | **Total**  **(n=54)** |
| --- | --- | --- | --- | --- | --- | --- | --- | --- |
| ORR (n/N, 95%CI) | 66.7% (2/3, 9.4-99.2%) | 66.7% (2/3, 9.4-99.2%) | 37.5% (3/8, 8.5-75.5%) | 72.7% (8/11, 39.0-94.0%) | 69.2% (9/13, 38.6-90.9%) | 53.8% (7/13, 25.1-80.8%) | 33.3% (1/3, 0.8-90.6%) | 59.3% (32/54, 45.0-72.4%) |
| DCR (n/N, 95%CI) | 66.7% (2/3, 9.4-99.2%) | 100% (3/3, 29.2-100%) | 87.5% (7/8, 47.3-99.7%) | 90.9% (10/11, 58.7-99.8%) | 100% (13/13, 75.3-100%) | 84.6% (11/13, 54.6-98.1%) | 33.3% (1/3, 0.8-90.6%) | 87.0% (47/54, 75.1-94.6%) |
| TTP (mo) |  |  |  |  |  |  |  |  |
| Median (95%CI) | 3.06 (0.92, 11.89) | NR (NR, NR) | 4.37 (0.92, NR) | NR (2.17, NR) | NR (3.71, NR) | NR (NR, NR) | NR (0.89, NR) | 11.89 (6.44, 11.89) |
| DOR (mo) |  |  |  |  |  |  |  |  |
| Median (95%CI) | 5.78 (0.69, 10.87) | NR (NR, NR) | NR (1.08, NR) | NR (1.25, NR) | NR (2.69, NR) | NR (NR, NR) | NR (NR, NR) | 10.87 (10.87, NR) |
| PFS (mo) |  |  |  |  |  |  |  |  |
| number of events (%) | 3/3 (100) | 0/3 (0) | 6/8 (75.0) | 3/11 (27.3) | 2/13 (15.4) | 1/13 (7.7) | 1/3 (33.3) | 16/54 (29.6) |
| Median (95%CI) | 3.06 (0.92, 11.89) | NR (NR, NR) | 4.37 (0.92, NR) | NR (2.17, NR) | NR (3.71, NR) | NR (NR, NR) | NR (0.89, NR) | 11.89 (5.03, 11.89) |

Abbreviations: ORR, objective response rate; CI, confidence interval; DCR, disease control rate; DOR, duration of response; TTP, time to progression; PFS, progression-free survival; NR, not reached

# Table S5. Summary of objective response rate by subgroup in dose escalation phase (n=54)

|  | **30mg** | **60mg** | **90mg** | **120mg** | **180mg** | **240mg** | **300mg** | **Total** |
| --- | --- | --- | --- | --- | --- | --- | --- | --- |
| **Prior ALK TKI treatment** **(n/N, 95%CI)** |  |  |  |  |  |  |  |  |
| ALK TKI naive | 66.7% (2/3, 9.4-99.2%) | 66.7% (2/3, 9.4-99.2%) | 50.0% (1/2, 1.3-98.7%) | 75.0% (6/8, 34.9-96.8%) | 83.3% (5/6, 35.9-99.6%) | 60.0% (3/5, 14.7-94.7%) | 100.0% (1/1, 2.5-100.0%) | 71.4% (20/28, 51.3-86.8%) |
| Crizotinib only | NA | NA | 40.0% (2/5, 5.3-85.3%) | 50.0% (1/2, 1.3-98.7%) | 50.0% (3/6, 11.8-88.2%) | 33.3% (2/6, 4.3-77.7%) | 0% (0/2, 0-84.2%) | 38.1% (8/21, 18.1-61.6%) |
| Crizotinib and other ALK TKI | NA | NA | NA | 100% (1/1, 2.5-100%) | 100% (1/1, 2.5-100%) | 100% (1/1, 2.5-100%) | NA | 100% (3/3, 29.2-100%) |
| ***ALK*-rearranged patients (n/N, 95%CI)** | 100% (2/2, 15.8-100%) | 66.7% (2/3, 9.4-99.2%) | 33.3% (2/6, 4.3-77.7%) | 80.0% (8/10, 44.4-97.5%) | 63.6% (7/11, 30.8-89.1%) | 63.6% (7/11, 30.8-89.1%) | 33.3% (1/3, 0.8-90.6%) | 63.0% (29/46, 47.5-76.8% |
| ALK TKI naive | 100% (2/2, 15.8-100%) | 66.7% (2/3, 9.4-99.2%) | NA | 85.7% (6/7, 42.1-99.6%) | 75.0% (3/4, 19.4-99.4%) | 75.0% (3/4, 19.4-99.4%) | 100% (1/1, 2.5-100%) | 81.0% (17/21, 58.1-94.6%) |
| Crizotinib only | NA | NA | 40.0% (2/5, 5.3-85.3%) | 50.0% (1/2, 1.3-98.7%) | 50.0% (3/6, 11.8-88.2%) | 40.0% (2/5, 5.3-85.3%) | 0% (0/2, 0-8.2%) | 40.0% (8/20, 19.1-63.9%) |
| Crizotinib and other ALK TKIs | NA | NA | NA | 100% (1/1, 2.5-100%) | 100% (1/1, 2.5-100%) | 100% (1/1, 2.5-100%) | NA | 100% (3/3, 29.2- 100%) |
| ***ROS1*-rearranged patients (n/N, 95%CI)** | 0% (0/1, 0-97.5%) | NA | 33.3% (1/3, 0.8-90.6%) | 0% (0/1, 0-97.5%) | 66.7% (2/3, 9.4-99.2%) | 0% (0/2, 0-84.2%) | 0 | 30.0% (3/10, 6.7-65.2%) |
| ALK TKI naive | 0% (0/1, 0-97.5%) | NA | 50.0% (1/2, 1.3-98.7%) | 0% (0/1, 0-97.5%) | 100% (2/2, 15.8-100%) | 0% (0/1, 0-97.5%) | NA | 42.9% (3/7, 9.9%-81.6%) |
| Crizotinib only | NA | NA | 0% (0/1, 0-97.5%) | NA | 0% (0/1, 0-97.5%) | 0% (0/1, 0-97.5%) | NA | 0% (0/3, 0-70.8%) |
| Crizotinib and other ALK TKIs | NA | NA | 0% (0/1, 0-97.5%) | NA | 0% (0/1, 0-97.5%) | NA | NA | 0% (0/2, 0-84.2%) |
| **Baseline CNS metastasis (n/N, 95%CI)** | 50% (1/2, 1.3-98.7%) | NA | 20% (1/5, 0.5-71.6%) | 0% (0/3, 0-70.8%) | 0% (0/5, 0-52.2%) | 0% (0/1, 0-52.2%) | 0% (0/2, 0-84.2%) | 9.1% (2/22, 1.1-29.2%) |
| **Baseline measurable CNS metastasis (n/N, 95%CI)** | 100% (1/1, 2.5-100%) | NA | 50% (1/2, 1.3-98.7%) | 0% (0/1, 0-97.5%) | 0% (0/1, 0-97.5%) | 0% (0/1, 0-97.5%) | NA | 33.3% (2/6,4.3-77.7%) |

Abbreviations: ALK, anaplastic lymphoma kinase; TKI, tyrosine kinase inhibitor; CI, confidence interval; ROS1, c-ros oncogene 1 receptor kinase; CNS, central nervous system; NA, not applicable.

# Table S6. Summary of objective response rate by subgroup in dose expansion phase (n=99)

|  | **120mg** | **180mg** | **Total** |
| --- | --- | --- | --- |
| **Prior ALK TKI treatment (n/N, 95%CI)** |  |  |  |
| ALK TKI naive | 72.2% (13/18, 46.5-90.3%) | 79.2% (19/24, 57.8-92.9%) | 76.2% (32/42, 60.5-87.9%) |
| Crizotinib only | 41.4% (12/29, 23.5-61.1%) | 42.9% (9/21, 21.8-66.0%) | 42.0% (21/50, 28.2-56.8%) |
| Crizotinib and other ALK TKI | 0 (0/2, 0-84.2%) | 66.7% (2/3, 9.4-99.2%) | 40.0% (2/5, 5.3-85.3%) |
| ***ALK*-rearranged patients (n/N, 95%CI)** | 53.2% (25/47, 38.1-67.9%) | 63.6% (28/44, 47.8-77.6%) | 58.2% (53/91, 47.4-68.5%) |
| ALK TKI naive | 76.5% (13/17, 50.1-93.2%) | 76.2% (16/21, 52.8-91.8%) | 76.3% (29/38, 59.8-88.6%) |
| received chemotherapy before | 77.8% (7/9, 40.0-97.2%) | 55.6% (5/9, 21.2-86.3%) | 66.7% (12/18, 41.0-86.7%) |
| chemotherapy naïve | 75.0%(6/8, 34.9-96.8%) | 91.7% (11/12, 61.5-99.8%) | 85.0% (17/20, 62.1-96.8%) |
| Crizotinib only | 44.4% (12/27, 25.5-64.7%) | 47.4% (9/19, 24.4-71.1%) | 45.7% (21/46, 30.9-61.0%) |
| Crizotinib and other ALK TKIs | 0 (0/2, 0-84.2%) | 66.7% (2/3, 9.4-99.2%) | 40.0% (2/5, 5.3-85.3%) |
| Other ALK TKIs | 0 (0/1, 0-97.5%) | 100% (1/1, 2.5-100.0%) | 50.0% (1/2, 1.3-98.7%) |
| ***ROS1*-rearranged patients (n/N, 95%CI)** | 0 (0/3, 0-70.8%) | 66.7% (4/6, 22.3-95.7%) | 44.4% (4/9, 13.7-78.8%) |
| Crizotinib naive | 0 (0/1, 0-97.5%) | 100% (4/4, 39.8-100%) | 80.0% (4/5, 28.4-99.5%) |
| Received Crizotinib before | 0 (0/2, 0-84.2%) | 0 (0/2, 0-84.2%) | 0 (0/4, 0-60.2%) |
| **Baseline CNS metastasis (n/N, 95%CI)** | 22.9% (8/35, 10.4-40.1%) | 8.7% (5/23, 7.5-43.7%) | 22.4% (13/58, 12.5-35.3%) |
| ALK TKI naive | 23.1% (3/13, 5.0-53.8%) | 28.6% (2/7, 3.7-71.0%) | 25.0% (5/20, 8.7-49.1%) |
| Crizotinib only | 25.0% (5/20, 8.7-49.1%) | 15.4% (2/13, 1.9-45.4%) | 21.2% (7/33, 9.0-38.9%) |
| Radiotherapy before | 16.7% (2/12, 2.1-48.4%) | 0 (0/7, 0-41.0%) | 10.5% (2/19, 1.3-33.1%) |
| Radiotherapy naïve | 26.1% (6/23, 10.2-48.4%) | 31.3% (5/16, 11.0-58.7%) | 28.2% (11/39, 15.0-44.9%) |
| **Baseline measurable CNS metastasis (n/N, 95%CI)** * | 45.5% (2/11, 16.7-76.6%) | 50.0% (2/4, 6.8-93.2%) | 46.7% (7/15, 21.3-73.4%) |
| ALK TKI naive | 33.3% (1/3, 0.8-90.6%) | 100% (1/1, 2.5-100%) | 50.0% (2/4, 6.8-93.2%) |
| Crizotinib only | 50.0% (4/8, 15.7-84.3%) | 50.0% (1/2, 1.3-98.7%) | 50.0% (5/10, 18.7-81.3%) |
| Radiotherapy before | 28.6% (2/7, 3.7-71.0%) | NA | 28.6% (2/7, 3.7-71.0%) |
| Radiotherapy naïve | 75.0% (3/4, 19.4-99.4%) | 31.3% (5/16, 11.0-58.7%) | 28.2% (11/39, 15.0-44.9%) |

* Data were intracranial objective response rate.

Abbreviations: ALK, anaplastic lymphoma kinase; TKI, tyrosine kinase inhibitor; CI, confidence interval; ROS1, c-ros oncogene 1 receptor kinase; CNS, central nervous system; NA, not applicable.

# Figure S1. The molecular structure of WX-0593


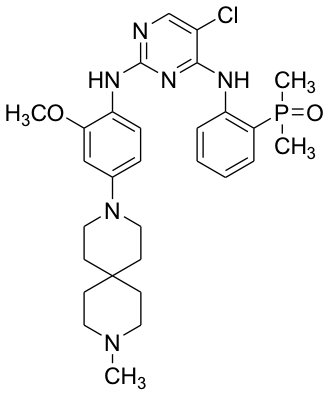


# Figure S2. (a) Mean plasma WX-0593 concentration-time curve of different dose groups for single-dosing in dose escalation phase (PK set). (b) Mean plasma WX-0593 concentration-time curve of different dose groups on D21 for multiple-dosing in dose escalation phase (PK set)

**
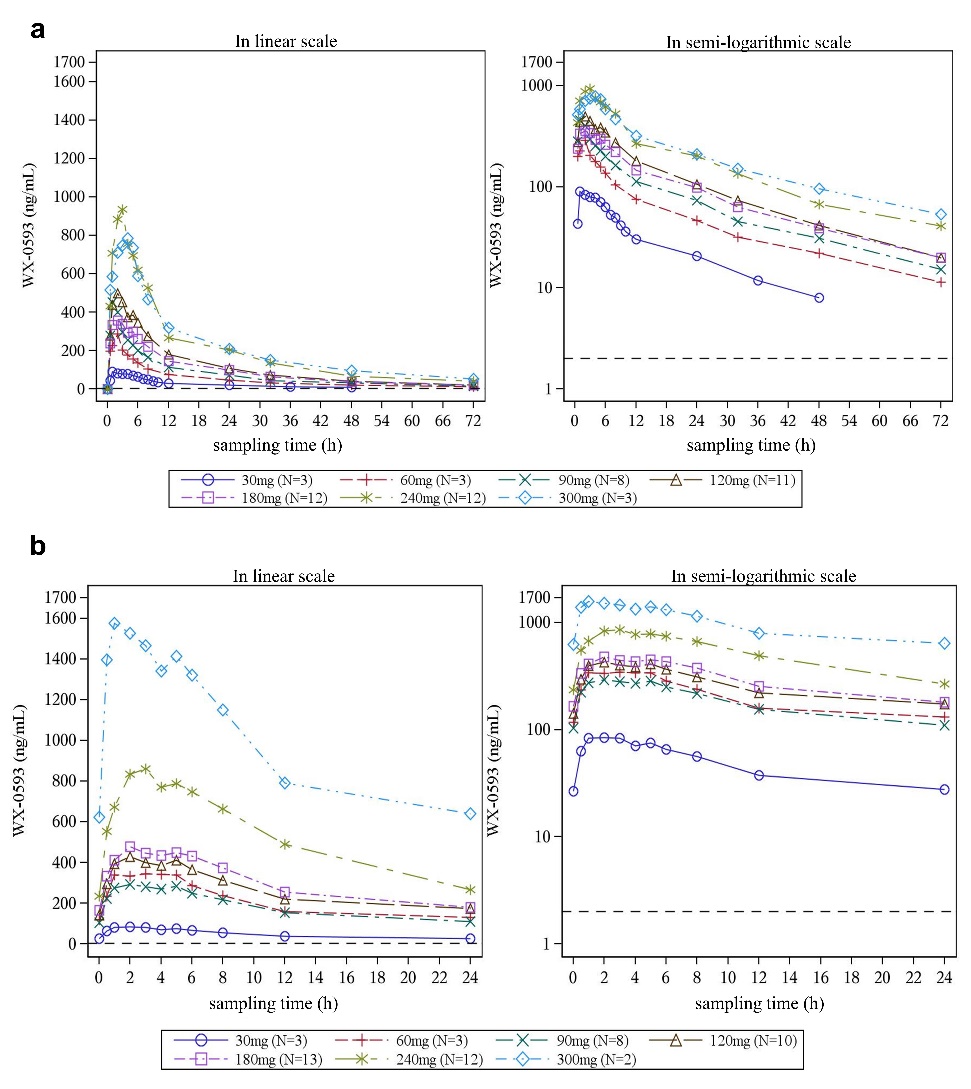
**

# Figure S3. (a) Duration of response of patients in dose-expansion phase (n=56); (b) Progression-free survival of patients in dose-expansion phase (n=99)

**
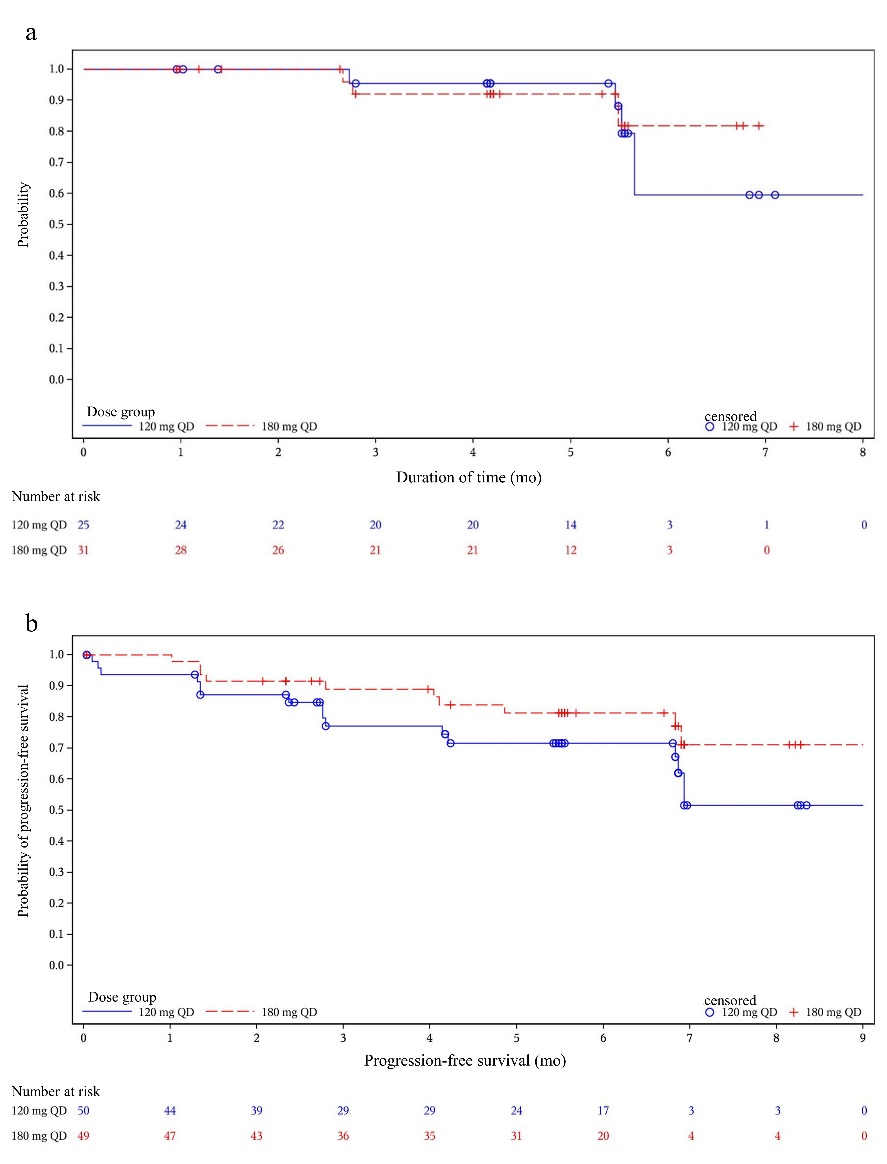
**
