# Supplementary material for: Safety and activity of WX-0593 (Iruplinalkib) in patients with ALK- or ROS1-rearranged advanced non-small cell lung cancer: a phase 1 dose-escalation and dose-expansion trial
Source: Signal Transduct Target Ther. 2022 Jan 28;7:25. doi: 10.1038/s41392-021-00841-8 (PMC8795197; doi:10.1038/s41392-021-00841-8)
Supplement: Supplementary file 2 — Protocol [file 41392_2021_841_MOESM2_ESM.docx]

Chemical Drug Class 1

CFDA Approval No.: 2016L10577, 2016L10578

**A Dose-Escalation/Expansion, Safety, Pharmacokinetics, and Efficacy Study of WX-0593 Tablets in Patients with Alk/Ros1-Positive Advanced Solid Tumors**

Protocol Number: WX0593-001

Version: 3.0

Version Date: Mar. 1, 2019

Lead Site: Cancer Hospital of the Chinese Academy of Medical Science

Statistical Unit: Nanjing CR Medicon Technology Co., Ltd.

Sponsor: Qilu Pharmaceutical Co., Ltd

CONFIDENTIALITY STATEMENT

All information contained in this protocol is owned by Qilu Pharmaceutical Co., Ltd, and is only provided to relevant medical institutions such as investigators, ethics committees, and regulatory authorities and should not be provided to a third party not involved in this study.

**Contact Information**

**Clinical Study Leading Site**

**Cancer Hospital of the Chinese Academy of Medical Science**

Address: No. 17 Panjiayuan Nanli, Chaoyang District, Beijing

Postal code: 100021

Principal Investigator: Shi Yuankai

Tel.: 010-67781331

**Sponsor**

**Qilu Pharmaceutical Co., Ltd.**

Address: No. 243 Gongyebei Road, Jinan

Postal code: 250100

Protocol Signature Page

Signature of Principal Investigator

I will fulfill my duties as an investigator in accordance with the Chinese GCP, and personally participate in or directly supervise this clinical study. I have read and confirmed this protocol (Protocol No.: WX0593-001; Version: 3.0; Version Date: Mar. 1, 2019), and agree with the science and ethics of this protocol. I will fulfill my duties in accordance with Chinese laws, the Declaration of Helsinki, the Chinese GCP, as well as this study protocol, notify the sponsor before modifying the protocol and obtain approval from the ethics committee before implementing the protocol, and I will not take additional actions unless it is necessary for the protection of safety, rights, and interests of subjects.

I will keep this study protocol confidential.

| Leading study site: Cancer Hospital of the Chinese Academy of Medical Science | | |
| --- | --- | --- |
| Shi Yuankai |  |  |
| Principal Investigator (Print) | Principal Investigator (Signature) | Date of Signature (MM/DD/YYYY) |

Protocol Signature Page

Participating Site Signature

I will fulfill my duties as an investigator in accordance with the Chinese GCP, and personally participate in or directly supervise this clinical study. I have read and confirmed this protocol (Protocol No.: WX0593-001; Version: 3.0; Version Date: Mar. 1, 2019), and agree with the science and ethics of this protocol. I will fulfill my duties in accordance with Chinese laws, the Declaration of Helsinki, the Chinese GCP, as well as this study protocol, notify the sponsor before modifying the protocol and obtain approval from the ethics committee before implementing the protocol, and I will not take additional actions unless it is necessary for the protection of safety, rights, and interests of subjects.

I will keep this study protocol confidential.

| Study Site: | | |
| --- | --- | --- |
|  |  |  |
| Investigator (Printed) | Investigator (Signature) | Date of Signature (MM/DD/YYYY) |

Protocol Signature Page

Signature of Statistical Unit

I have read and confirmed this protocol (protocol no.: WX0593-001, version: 3.0, version date: Mar. 1, 2019). I agree to fulfill my duties in accordance with Chinese laws, the Declaration of Helsinki, Chinese GCP, and this study protocol.

I will keep this study protocol confidential.

| Statistical Unit: Nanjing CR Medicon Technology Co., Ltd. |
| --- |

| Peng Yumei |  |  |
| --- | --- | --- |
| Supervisor (Printed) | Supervisor (Signature) | Date of Signature (MM/DD/YYYY) |

Protocol Signature Page

Sponsor Signature

I have read and confirmed this protocol (protocol no.: WX0593-001, version: 3.0, version date: Mar. 1, 2019). I agree to fulfill my duties in accordance with Chinese laws, the Declaration of Helsinki, Chinese GCP, and this study protocol.

| Sponsor: Qilu Pharmaceutical Co., Ltd | | |
| --- | --- | --- |
| Yu Shunjiang |  |  |
| Project Manager (Printed) | Project Manager (Signature) | Date of Signature (MM/DD/YYYY) |

**TABLE OF CONTENTS**

[Protocol Abstract 4](#_Toc59449738)

[Schedule of events — dose escalation phase 12](#_Toc59449739)

[Abbreviations and Definitions 16](#_Toc59449740)

[1 BACKGROUND 19](#_Toc59449741)

[1.1 Medication Background 19](#_Toc59449742)

[1.2 Preclinical Studies 21](#_Toc59449743)

[2 OVERALL TRIAL DESIGN 30](#_Toc59449744)

[3 TRIAL OBJECTIVE 31](#_Toc59449745)

[3.1 Study Objectives 31](#_Toc59449746)

[3.2 Study endpoints 32](#_Toc59449747)

[4 Study design 33](#_Toc59449748)

[4.1 Determination of starting dose 34](#_Toc59449749)

[4.2 Dose Arm Setting And Dose Escalation 36](#_Toc59449750)

[4.3 Determination criteria for dose-limiting toxicity (DLT) and maximum tolerated dose (MTD) 37](#_Toc59449751)

[4.4 Pharmacokinetic study 38](#_Toc59449752)

[5 SUBJECT SELECTION 39](#_Toc59449753)

[1.1 Inclusion Criteria 39](#_Toc59449754)

[5.1 Exclusion Criteria 40](#_Toc59449755)

[5.2 Criteria for Discontinuation 41](#_Toc59449756)

[6 TREATMENT REGIMEN 42](#_Toc59449757)

[6.1 Dose Regimen 42](#_Toc59449758)

[6.2 Investigational Product Packaging, Specification, and Storage 42](#_Toc59449759)

[6.3 Management of the Investigational Product 43](#_Toc59449760)

[7 CONCOMITANT TREATMENTS AND DOSE ADJUSTMENTS 43](#_Toc59449761)

[7.1 Drugs Prohibited During the Study 43](#_Toc59449762)

[7.2 Drugs That Can Be Used with Discretion During the Study 43](#_Toc59449763)

[8 SCHEDULE OF TRAIL 45](#_Toc59449764)

[8.1 Screening Period (within 28 Days Prior to the First Dose) 45](#_Toc59449765)

[8.2 Treatment period (after single dosing and Cycle 1 of repeated dosing, applicable for the dose escalation phase; population expansion phase begins with repeated dosing) 46](#_Toc59449766)

[8.3 Continuous treatment period (Week 4/5-) 47](#_Toc59449767)

[8.4 Safety Follow-Up Period 49](#_Toc59449768)

[8.5 Telephone follow-up (population expansion phase) 49](#_Toc59449769)

[8.6 End of study 49](#_Toc59449770)

[9 SAFETY EVALUATION 50](#_Toc59449771)

[9.1 Definitions 50](#_Toc59449772)

[9.2 Potential Adverse Drug Reactions 51](#_Toc59449773)

[9.3 Criteria for AE Severity 51](#_Toc59449774)

[9.4 Criteria for Causality Assessment Between AEs and Investigational Product 52](#_Toc59449775)

[9.5 AE Reporting and Management 52](#_Toc59449776)

[9.6 Serious Adverse Event Reporting and Management 53](#_Toc59449777)

[10 PHARMACOKINETIC STUDY 54](#_Toc59449778)

[10.1 Sample Collection and Storage 54](#_Toc59449779)

[10.2 Sampling Time Point 54](#_Toc59449780)

[10.3 Test method 55](#_Toc59449781)

[10.4 PK Parameters Analysis 56](#_Toc59449782)

[11 TREATMENT RESPONSE EVALUATION 56](#_Toc59449783)

[11.1 Baseline Assessment 56](#_Toc59449784)

[11.2 Efficacy Evaluation 57](#_Toc59449785)

[12 STUDY MANAGEMENT 58](#_Toc59449786)

[12.1 Ethics and Informed Consent 58](#_Toc59449787)

[12.2 Investigational Product Management 59](#_Toc59449788)

[12.3 Protocol Revisions 59](#_Toc59449789)

[12.4 Monitoring 59](#_Toc59449790)

[12.5 Auditing 60](#_Toc59449791)

[12.6 Quality Control and Assurance 60](#_Toc59449792)

[12.7 Data Management 60](#_Toc59449793)

[12.8 Protocol Violations 61](#_Toc59449794)

[12.9 Document Storage 62](#_Toc59449795)

[12.10 Publication of Study Results 62](#_Toc59449796)

[12.11 Duties and Regulations 62](#_Toc59449797)

[13 STATISTICAL ANALYSIS 63](#_Toc59449798)

[13.1 General Principles 63](#_Toc59449799)

[13.2 Management of missing values 63](#_Toc59449800)

[13.3 Selection of Statistical Analysis Data 63](#_Toc59449801)

[13.4 Statistical Analysis Plan 64](#_Toc59449802)

[14 TRIAL PROGRESS 65](#_Toc59449803)

[REFERENCES 66](#_Toc59449804)

[Appendix I: NCI-Common Terminology Criteria 4.03 for Toxicity 68](#_Toc59449805)

[Appendix II: ECOG Performance Status 75](#_Toc59449806)

[Appendix III: Response Evaluation Criteria in Solid Tumors Version 1.1 76](#_Toc59449807)

[Appendix V: Deviation Table of Blood Collection Timepoints 90](#_Toc59449808)

[Appendix IV: Principles of Replacement Doses 92](#_Toc59449809)

Protocol Abstract

| **Study Title** | A Dose-Escalation/Expansion, Safety, Pharmacokinetics, and Efficacy Study of WX-0593 Tablets in Patients with ALK/ROS1-positive Advanced Solid Tumors |
| --- | --- |
| **Protocol No.** | WX0593-001 |
| **Version** | 3.0 |
| **Version Date** | March 1, 2019 |
| **Sponsor** | Qilu Pharmaceutical Co., Ltd. |
| **Principal Investigator** | Shi Yuankai |
| **Lead Site** | Cancer Hospital of the Chinese Academy of Medical Science |
| **Study Objective** | 1. **Dose escalation phase**   **Primary objective:** To observe the safety and tolerability and to determine the dose-limiting toxicity (DLT), maximum tolerated dose (MTD), and the subsequent recommended dose of WX-0593 oral tablets in patients with ALK-positive (translocation/over-expression) or ROS1-positive advanced solid tumors.  **Secondary objectives:** To observe and analyze the pharmacokinetic characteristics of WX-0593 tablets in patients with ALK-positive (translocation/over-expression) or ROS1-positive advanced solid tumors; to preliminarily observe the clinical efficacy of WX-0593 tablets in patients with ALK-positive (rearrangement/over-expression) or ROS1-positive advanced solid tumors.   1. **Population expansion phase**   **Primary Objective:** To preliminarily evaluate the efficacy of WX-0593 tablets in patients with ALK-positive (translocation/over-expression) or ROS1-positive advanced non-small cell lung cancer (NSCLC)  **Secondary Objective**: To observe the safety of WX-0593 tablets in patients with ALK-positive (translocation/over-expression) or ROS1-positive advanced NSCLC.   1. **Exploratory study**  - To explore disease progression-related ALK gene mutation, ROS1 gene mutation, and other molecular mechanisms of drug resistance after treatment with crizotinib, WX-0593, or other ALK inhibitors. - To measure WX-0593 concentration in cerebrospinal fluid and preliminarily assess the efficacy of the investigational product in patients with brain metastases and its drug concentration basis. - To assess the overall survival (OS) of patients treated with WX-0593. |
| **Study Endpoints** | 1. **Dose escalation phase**   **Primary endpoints:**   - To determine the MTD, DLT, and subsequent recommended dose - Incidence of adverse events, including adverse events (AEs), serious adverse events (SAEs), and treatment-emergent adverse events (TEAEs). Causality is determined by the investigator.   **Secondary endpoints:**   - Pharmacokinetic (PK) parameters: T_max_, C_max_, AUC, and t_1/2_ after a single dose; C_ssmin_, C_ssmax_, C_ss-av_, t_1/2_, AUC_ss_, DF, V_z_, and CLs after repeated doses. - Preliminary efficacy: The evaluation indicators include objective response rate (ORR), progression-free survival (PFS), disease control rate (DCR), time to progression (TTP), duration of response (DOR) and intracranial ORR.  1. **Population expansion phase**   **Primary endpoints**: ORR  **Secondary endpoints:**   - PFS, DCR, TTP, DOR, CNS objective response rate, etc. - Incidence of adverse events, including AEs, SAEs, and TEAEs. Causality is determined by the investigator.  1. **Exploratory study**  - The relationship between ALK gene mutation, ROS1 gene mutation, and other molecular changes in blood and tumor tissues and antineoplastic activity of WX-0593. - (Some patients with brain metastases will be selected) to measure WX-0593 concentration in cerebrospinal fluid and preliminarily assess the efficacy of the investigational product in patients with brain metastases and its drug concentration basis. - OS |
| **Study Design and**  **Treatment regimen** | This study includes a dose escalation phase and a subsequent population expansion phase. After the maximum tolerated dose (MTD) is determined in the dose escalation phase, 120 mg QD and 180 mg QD are selected for the population expansion phase study to determine the optimal target dose for subsequent studies. However, corresponding adjustments during the study period can be jointly determined by the investigator and sponsor based on the results of previous clinical studies. Before administration of 120 mg QD or 180 mg QD in subjects, it is recommended that a run-in dose of 60 mg QD be administered for 7 continuous days. If the subject is tolerable, the dose is escalated to 120 mg QD or 180 mg QD and administered until disease progression, unacceptable toxicity, voluntary withdrawal, loss to follow-up, start of another anti-cancer therapy, death, or end of study (whichever occurs first). After efficacy and safety comparison data between two groups were obtained at the population expansion phase, the optimal target recommended dose will be determined for subsequent phase II studies.   1. **Investigational Product**   WX-0593 tablet, supplied by Qilu Pharmaceutical Co., Ltd, specification: 30mg; 60 mg   1. **Dose escalation phase**   **Study Design**  This is a multi-center, single-arm, open-label, dose-escalation clinical trial.  Patients with ALK-positive or ROS1-positive advanced solid tumors were planned to be enrolled in the dose-escalation trial consisting of a single dose and repeated doses. The pharmacokinetic study at a single dose and repeated doses was carried out simultaneously with the tolerability study.  The proposed escalation doses are as follows: 30 mg, 60 mg, 90 mg, 120 mg, 180 mg, 240 mg, 300 mg, and 360 mg. During the trial, dose modification may be performed by the investigator and the sponsor according to preliminary data. If DLT was not observed at the highest dose arm, whether to continue the dose-escalation trial should be decided by the investigator and the sponsor. The 3+3 dose escalation principle was adhered to and 39–60 patients were to be enrolled.  **Treatment regimen**   - After single dosing on Day 1, pharmacokinetic blood sampling will be simultaneously carried out and the subject will be observed for 4 days. Repeated dosing will start on Day 5 and dosing will be carried out orally on an empty stomach in fasting condition once daily. PK blood sampling will be carried out on Day 21 of repeated dosing. One cycle consists of 21 days of repeated dosing. - The dose escalation study for the next dose arm can begin after the last subject in the dose arm completed the first cycle of treatment and no DLT is observed. Subjects in this dose arm will continue dosing with the original dose. - The first tumor assessment is performed after 3 weeks of repeated dosing, then once every 6 weeks subsequently. Treatment is continued for CR/PR or SD and is terminated for PD.  1. **Population expansion phase**   **Study Design**  This study is a single-arm, open-label, multi-center clinical trial in China. Patients with ALK-positive (translocation/over-expression) or ROS1-positive advanced NSCLC will be enrolled for a repeated-dose efficacy and safety study.  After MTD is basically determined, 120 mg QD and 180 mg QD are selected for the population expansion phase study. However, corresponding adjustments during the study period can be jointly determined by the investigator and sponsor based on the results of preliminary clinical studies. Before administration of 120 mg QD or 180 mg QD in subjects, a run-in dose of 60 mg QD is administered for 7 continuous days. If the subject is tolerable, the dose is escalated to 120 mg QD or 180 mg QD and administered until disease progression, unacceptable toxicity, voluntary withdrawal, loss to follow-up, start of another anti-cancer therapy, death, or end of study (whichever occurs first). 30–70 patients were planned to be enrolled per arm and 60–140 patients were planned to be enrolled in total.  **Treatment regimen**   - Prior to the dose of 120 mg QD or 180 mg QD, the subject will be given a dose of 60 mg QD for 7 consecutive days, and the dose will be increased to 120 mg QD or 180 mg QD if it is tolerable. The drug should be administered orally in fasting condition once daily for 21 days continuously for each cycle. - Efficacy evaluation is carried out once every 6 weeks in the first 48 weeks, then once every 12 weeks subsequently. Treatment is to be continued for CR/PR or SD and terminated for PD. Subjects must repeat the efficacy assessment 4 weeks after the first documented CR/PR. If the confirmation assessment is < 4 weeks from the next scheduled tumor assessment, then the next assessment is skipped.  1. **Exploratory study**  - Peripheral blood samples will be collected and tumor samples will be collected as much as possible from ALK-positive patients who are resistant to crizotinib or other ALK inhibitors and ROS1-positive patients who are resistant to crizotinib for an exploratory study on drug resistance genes. - During the trial, peripheral blood samples will be collected and tumor samples will be collected as much as possible from subjects with progressive disease for an explanatory study on drug resistance genes. - For patients with brain metastases, after comprehensive judgment by the investigator and after patient consent is obtained, cerebrospinal fluid can be collected at specific time points for drug concentration measurement and for comparison with plasma drug concentration to determine the relationship between efficacy and concentration of WX-0593 in patients with brain metastases. - To assess the OS of patients treated with WX-0593. |
| **Inclusion/Exclusion Criteria** | 1. **Inclusion criteria** 2. Above 18 years old; 3. Gender: Male or female; 4. ECOG PS score 0/1; 5. Expected survival no less than 12 weeks; 6. Patients should have at least one measurable lesion (RECIST 1.1); Lesions previously treated with radiotherapy can be considered target lesions only if there is clear evidence of progression after radiotherapy; 7. The following patients who are proven to be ALK-positive (translocation/over-expression) or ROS1-positive by histopathological or cytological tests in Grade III hospitals (Subjects in the population expansion phase are ALK- or ROS1-positive advanced NSCLC patients):  - Patients with advanced malignant tumors (such as NSCLC, lymphoma, inflammatory myofibroblastic tumor, etc.) who failed standard treatment [such as resistance to ALK inhibitors (excluding brigatinib and other structurally similar drugs), chemotherapy failure, etc.]; - Advanced NSCLC patients who cannot receive or tolerate chemotherapy; - Advanced NSCLC patients who cannot receive ALK inhibitors due to financial reasons;  1. Patients should provide tissue biopsy samples or archived tumor tissue samples if possible before enrollment; 2. Absence of brain metastasis, or asymptomatic brain metastasis, or symptomatic brain metastasis that has remained stable for more than 4 weeks after treatment; 3. Organ functions should meet the following requirements (blood components, cell growth factors, drugs that stimulate the growth of WBC or platelets, or drugs used to correct anemia are not permitted within 14 days prior to the laboratory test): ANC ≥ 1.5 × 10^9^/L; PLT ≥ 100 × 10^9^/L; Hb ≥ 90 g/L; TBIL ≤ 1.5 × ULN (TBIL ≤ 3.0 × ULN and DBIL ≤ 1.5 × ULN if Gilbert's syndrome); ALT and AST ≤ 2.5 × ULN; ALT and AST ≤ 5 × ULN if liver metastasis; Cr ≤ 1.5 × ULN; LVEF ≥ 50%; 4. Any surgery and prior radiotherapy (except for palliative radiotherapy)/procedures must have been completed at least 4 weeks prior to the treatment with the investigational product. Palliative radiotherapy must have been completed 48 hours prior to the treatment; 5. Any toxicity associated with previous anti-cancer treatment must have recovered to Grade ≤ 1 (except for alopecia); 6. The subject understands and voluntarily provides informed consent. 7. **Exclusion Criteria** 8. Patients with leptomeningeal metastases; 9. Any clinically significant cardiovascular disease within 3 months prior to the first dose of the investigational product, including but not limited to: myocardial infarction, severe/unstable angina, coronary artery/peripheral artery bypass graft, congestive heart failure, cerebrovascular accident (including transient ischemic attack); 10. Patients with NCI-CTCAE Grade ≥ 2 arrhythmia, any grade of uncontrolled atrial fibrillation, or two continuously corrected QT interval (QTc) > 480 ms on ECG during screening; 11. Concomitant use of medications that may cause QTc prolongation or induce torsades de pointes within 14 days prior to the first dose of the investigational product or during treatment; 12. Grade ≥ 3 peripheral neuropathy (CTCAE V4.03); 13. Continuous use of corticosteroids for more than 30 days, or the need for chronic use of corticosteroids or other immunosuppressants; 14. A large area of diffuse/interstitial pulmonary fibrosis, or a known history of Grade 3 or 4 interstitial pulmonary fibrosis disease, which includes a history of pneumonitis, hypersensitivity pneumonitis, interstitial pneumonitis, interstitial lung disease, obliterative bronchiolitis, and pulmonary fibrosis, but does not include a history of radiation pneumonitis; 15. Patients with Grade > 1 nausea, vomiting, or diarrhea (CTCAE V4.03), other GI dysfunction or GI disease that may potentially affect drug absorption (such as ulcerative disease or malabsorption syndrome); 16. Patients currently on warfarin sodium (Coumadin) or other coumadin derivative anticoagulant treatment, or patients with a bleeding tendency or coagulation disorders; 17. Subject received other clinical trial treatment within 1 month prior to the first dose of the investigational product (if the medication received is a marketed drug, then refer to exclusion criteria #12); 18. Acute or chronic infectious disease, active hepatitis (hepatitis A, hepatitis B, or hepatitis C), or known HIV infection; 19. No more than 2 weeks between the most recent use of another anti-cancer treatment (half-life ≤ 3 days) and the first dose of the investigational product, or the most recent use of another anti-cancer treatment (half-life > 3 days) is less than 4 weeks. Patients may begin WX-0593 treatment 1 week after the last dose of crizotinib. 20. Patients who cannot suspend the use of a strong CYP3A4 inducer or inhibitor at least one week prior to this study and during the study. These drugs include but are not limited to carbamazepine, phenobarbital, phenytoin, rifabutin, rifampicin, rifapentine, tipranavir, ritonavir, St. John's wart, and ketoconazole; 21. Patients who cannot suspend the use of a CYP3A4 substrate at least one week prior to this study and during the study, with low therapeutic index; 22. Pregnant or lactating female patients or a positive pregnancy test at baseline for females of childbearing potential; 23. Female patients of childbearing age who are unwilling to use effective contraceptive measures or male patients who plan to have children during the study and within 6 months after the study; 24. Concurrent diseases that may seriously affect patient safety or impact patient completion of the study as determined by the investigator (such as clinically uncontrolled hypertension (blood pressure > 160/110 mmHg), severe diabetes, thyroid disease, etc.); 25. Drug or alcohol abuse; Alcoholism refers to consumption of 4 or more alcoholic drinks during 2 hours. One alcoholic drink refers to 150 mL of wine, 350 mL of beer, or 50 mL of 80-proof distilled spirits (or equivalent alcohol concentration). 26. History of definitive neurological or mental disorder, including epilepsy and dementia; 27. Patients with other malignant tumors within 5 years prior to screening (except for cured basal cell carcinoma of the skin, cervical carcinoma *in situ*, and papillary thyroid carcinoma); 28. Patients with added risks associated with the study, or those who may interfere with the interpretation of study results as determined by the investigator, or those deemed unsuitable for this study by the investigator and/or the sponsor. |
| **Safety Evaluation** | Refer to the Schedule of Events for all safety evaluations. Safety evaluations will be conducted based on medical history, signs and symptoms during the study, laboratory examinations, vital signs, physical examination, ECG, and LVEF. Standard safety monitoring and grading will be conducted using NCI-CTCAE 4.03 (see Appendix I). |
| **Pharmacokinetic Assessment** | At the dose escalation phase, after the end of the tolerability study for each arm, provided that there was good safety, the investigator will enroll additional patients in 3–6 dose arms for single dose and repeated dose pharmacokinetic studies. At least 8 patients in each arm are included in the PK studies (ensuring that there are at least 8 evaluable subjects in each arm). The plasma concentration of WX-0593 will be determined using LC-MS. |
| **Efficacy assessment** | The investigator will carry out the first efficacy evaluation based on RECIST 1.1 after 3 weeks of repeated dosing at the dose escalation phase and subsequent efficacy evaluations will be carried out once every 6 weeks. At the population expansion phase, efficacy evaluation will be carried out once every 6 weeks in the first 48 weeks and then once every 12 weeks after Week 48 until radiology shows progressive disease (PD). At the population expansion phase, subjects must repeat the efficacy assessment 4 weeks after the first documented CR/PR. If the confirmation assessment is < 4 weeks from the next scheduled tumor assessment, then the next assessment is skipped. A CT or MRI scan must be performed as soon as disease progression is suspected. Except for PD, subjects who complete treatment will proceed with imaging assessments as planned until progressive disease, start of a new anti-cancer therapy, withdrawal of consent, loss to follow-up or death. |
| **Statistical methods** | **Safety analysis:** AEs were coded using MedDRA and graded as per NCI CTCAE V4.03. The number of cases, number of subjects, and incidence of all AEs, drug-related AEs, SAEs, drug-related SAEs, AEs resulting in drop-out, and deaths associated with AEs were summarized.  All completed examinations such as physical examinations, ECG, and laboratory parameters (based on the clinician's judgment) and their descriptive statistics for each visit were summarized using a cross table involving pre-treatment and post-treatment values.  PK analysis: C_max_, C_min_, AUC_0–t_, AUC_0–∞_, T_max_, t_1/2_, CL, and V_z_ will be calculated using the PK software WinNonlin.  **Efficacy analysis:** Objective response rate (ORR), progression-free survival (PFS), disease control rate (DCR), time to progression (TTP), duration of response (DOR), CNS ORR and their respective 95% confidence intervals were determined. |
| **End of study** | For subjects who can benefit from the treatment as determined by the investigator, the sponsor will supply the drug to every subject after the first dose of the investigational product until progressive disease, intolerability, active withdrawal by patient, death, or end of study (whichever comes first). If the subject has not achieved PD at the end of the study and may still benefit from the study as comprehensively determined by the investigator, the sponsor will continue to supply the investigational product for free but examinations will no longer be arranged and safety and efficacy data will not be collected.   - - - 1. **Dose escalation phase**   Statistical analysis and trial summary can be carried out for the entire trial after all PK blood sampling has been completed for the last subject. The entire dose escalation study can be completed two years after the last subject is enrolled.   - - - 1. **Population expansion phase**   After the first efficacy assessment and efficacy confirmation is completed for the last subject, observation is continued until 12 weeks since enrollment. Then, statistical analysis and trial summary can be carried out. The entire population expansion study can be completed when survival follow-up is 2 years after the last subject is enrolled. |
| **Trial Progress** | Anticipated during: Sep. 2017 to Dec. 2020. |

Schedule of events — dose escalation phase

|  | Screening period | | Treatment period | | | | | | Continuous treatment period | Follow-up |
| --- | --- | --- | --- | --- | --- | --- | --- | --- | --- | --- |
|  | Visit 1  Pre-dose  (-28–0) | Visit 2  Pre-dose  (-7–0) | Visit 3  Single dose  Day 1 | Visit 4  After single dose  Day 4 | Visit 5  Repeated dose  Day 1 | Visit 6  After repeated dose  Day 7 | Visit 7  After repeated dose  Day 14 | Visit 8  After repeated dose  Day 21 | Every 1/2 dosing cycles^d^ | Retest within 28 days after discontinuing study treatment |
| **Signing of informed consent form** | √ |  |  |  |  |  |  |  |  |  |
| **Demographics collection** | √ |  |  |  |  |  |  |  |  |  |
| **Medical history and medication history** | √ |  |  |  |  |  |  |  |  |  |
| **ALK/ROS1**  **Status determination** | √ |  |  |  |  |  |  |  |  |  |
| **Cancer treatment history** | √ |  |  |  |  |  |  |  |  |  |
| **Vital signs measurement** |  | √ | √ | √ |  | √ | √ | √ | √ | √ |
| **Physical examination** |  | √ |  | √ |  | √ | √ | √ | √ | √ |
| **ECOG PS score** | √ |  |  | √ |  |  |  | √ | √ |  |
| **Blood routine** |  | √ |  | √ |  | √ | √ | √ | √ | √ |
| **Blood biochemistry** |  | √ |  | √ |  | √ | √ | √ | √ | √ |
| **Urinalysis** |  | √ |  | √ |  | √ | √ | √ | √ | √ |
| **Routine stool test** |  | √ |  | √ |  | √ |  | √ |  |  |
| **Pre-transfusion blood screening** |  | √ |  |  |  |  |  |  |  |  |
| **Coagulation function** |  | √ |  |  |  |  |  |  |  |  |
| **ECG** |  | √ | √c | √ |  | √ | √ | √ | √ | √ |
| **Color doppler echocardiography** |  | √ |  |  |  |  |  | √ | √▽ | √▽ |
| **test** |  | √ |  |  |  |  |  |  |  |  |
| **Distributing daily log** |  |  | √ | √ | √ | √ | √ | √ | √ |  |
| **Daily log evaluation** |  |  |  | √ | √ | √ | √ | √ | √ | √ |
| **Radiology examination (CT/MRI)** | √ |  |  |  |  |  |  | √ | √**★** |  |
| **Investigational product administration** |  |  | √a |  | √b | √b | √b | √b | √b |  |
| **PK sampling** |  |  | √ |  | √ | √ | √ | √ |  |  |
| **Concomitant medication** | √ |  | √ | √ | √ | √ | √ | √ | √ | √ |
| **Adverse events** | √ | √ | √ | √ | √ | √ | √ | √ | √ | √ |

Note: ★Every 2 cycles of continuous administration (42 days ± 7d), **▽**when deemed necessary by the investigator a: One dose on D1, no dose given on D2–4, b: Once daily, c: Test at 4 h ± 30 min and 24 h ± 30 min after a single dose, ECG monitoring during single dose administration, usually for 2 h. **If electrocardiogram abnormalities are noted, monitoring would be continued until 1 h after electrocardiogram returns to normal; d: every 6 weeks after Week 51 (± 3d)**

**Schedule of events-population expansion phase**

|  | **Screening period** | | **Treatment period** | | | **Continuous treatment period** | **Follow-up** | **Survival visit** |
| --- | --- | --- | --- | --- | --- | --- | --- | --- |
|  | **Pre-dose**  **(-28–0)** | **Pre-dose**  **(-7–0)** | **Day 1  (-1d–1d)** | **Day 7**  **(± 2d)** | **Day 21**  **(± 2d)** | **Every 2/4 dosing cycles^c^**  **(± 3d)** | **Review within 28 days after discontinuing study treatment^d^** | **(12w ± 7d)** |
| **Signing of informed consent form** | √ |  |  |  |  |  |  |  |
| **Demographics collection** | √ |  |  |  |  |  |  |  |
| **Medical history and medication history** | √ |  |  |  |  |  |  |  |
| **ALK/ROS1 status determination** | √ |  |  |  |  |  |  |  |
| **Cancer treatment history** | √ |  |  |  |  |  |  |  |
| **Vital signs measurement** |  | √ |  | √ | √ | √ | √ |  |
| **Physical examination** |  | √ |  | √ | √ | √ | √ |  |
| **ECOG PS score** | √ |  |  |  | √ | √ |  |  |
| **Laboratory tests^a^** |  | √ |  | √ | √ | √ | √ |  |
| **Pre-transfusion blood screening** |  | √ |  |  |  |  |  |  |
| **Coagulation function** |  | √ |  |  |  |  |  |  |
| **ECG** |  | √ |  | √ | √ | √ | √ |  |
| **Color doppler echocardiography** |  | √ |  |  | √**^b^** | √▽ | √▽ |  |
| **Pregnancy test (for females of childbearing age only)** |  | √ |  |  |  |  |  |  |
| **Distributing daily log** |  |  | √ | √ | √ | √ |  |  |
| **Daily log evaluation** |  |  |  | √ | √ | √ | √ |  |
| **Radiology examination (CT/MRI)** | √ |  |  | √△ |  | √**★** |  |  |
| **Investigational product administration** |  |  | √ | √ | √ | √ |  |  |
| **Concomitant medication** | √ |  | √ | √ | √ | √ | √ |  |
| **Adverse events** | √ | √ | √ | √ | √ | √ | √ |  |
| **Survival information and subsequent treatment/medication** |  |  |  |  |  |  | √ | √ |

Note: a. Laboratory tests include blood routine, blood biochemistry, urinalysis, and stool routine, of which stool routine is only carried out at the screening phase, D7 ± 2d, D21 ± 2d, and when necessary as determined by the investigator; b: The window period is ± 3d; c: Every 6 cycles (42 days) in the first 48 weeks and every 12 weeks (84 days) after Week 48 weeks; d: Not carried out if the patient starts new anti-cancer treatment within 2 weeks after drug discontinuation, please complete at before starting anti-cancer treatment at other time points within 28 days. Not carried out if the patient starts new anti-cancer treatment without informing the investigator. ▽Necessary as determined by the investigator; △After repeated dosing with 60 mg QD for 7 days and the investigator deemed that a chest CT is necessary based on the clinical condition to observe safety; ★ The window period is ± 7 d. At the population expansion phase, efficacy confirmation must be carried out after 4 weeks if the patient is assessed to be CR/PR in the first imaging assessment. If efficacy confirmation is less than 4 weeks from the next imaging assessment, the next imaging assessment will be skipped.

Abbreviations and Definitions

| Abbreviations and Terms | Interpretation |
| --- | --- |
| AE | Adverse events |
| ALB | Albumin |
| ALT | Alanine aminotransferase/serum glutamic pyruvic transaminase |
| ALK | Anaplastic lymphoma kinase |
| AST | Aspartate aminotransferase/serum glutamic-oxaloacetic transaminase |
| AUC_0~t_ | The area under the concentration-time curve from concentration 0 to the last measurable concentration |
| AUC_0~∞_ | The area under the concentration-time curve from time 0 to infinity |
| AUC_ss_ | The area under the concentration-time curve during steady state |
| BMI | Body mass index |
| BP | Blood pressure |
| BUN | Urea nitrogen |
| Ca | Serum calcium |
| CHOL | Cholesterol |
| CK | Creatinine kinase |
| C_max_ | Maximum serum drug concentration |
| C_ssmax_ | Maximum blood drug concentration at steady state |
| C_ssmin_ | Minimum blood drug concentration |
| C_ssav_ | Average blood drug concentration at steady state |
| CL/F | Apparent clearance/F |
| Cr | Creatinine |
| CR | Complete response |
| CRF | Case Report Forms |
| CYP450 | Cytochrome P450 |
| DBIL | Direct bilirubin |
| DF | Degree of fluctuation at steady state |
| DLT | Dose-limiting toxicity |
| ECG | Electrocardiogram |
| FISH | Fluorescence in situ hybridization |
| GCP | Good Clinical Practice |
| GLU | Blood glucose |
| HB | Hemoglobin |
| HCT | Hematocrit |
| HDL | High-density lipoprotein |
| IHC | Immunohistochemistry |
| LC-MS | Liquid chromatography-mass spectrometry |
| LDH | Lactate dehydrogenase |
| LDL | Low-density lipoprotein |
| LOAEL | Lowest-observed-adverse-effect level |
| MTD | Maximum tolerated dose |
| NOAEL | No-observed-adverse-effect level |
| PD | Progressive disease |
| PLT | Platelet |
| PR | Partial response |
| RBC | Red blood cell count |
| SAE | Serious adverse event |
| SD | Standard deviation |
| SFDA | State Food and Drug Administration |
| SOP | Standard Operating Procedure |
| TBA | Total bile acids |
| TBIL | Total bilirubin |
| TG | Triglycerides |
| TP | Total protein |
| UA | Uric acid |

**A Dose-Escalation/Expansion, Safety, Pharmacokinetics, and Efficacy Study**

**of WX-0593 Tablets in Patients with ALK/ROS1-positive Advanced Solid Tumors**

# BACKGROUND

## Medication Background

Lung cancer has one of the highest incidence and mortality rates worldwide. In 2014, the World Health Organization published the World Cancer Report 2014, which shows that lung cancer ranks first in incidence and mortality among all cancers worldwide with 1.8 million new cases and 1.6 million deaths each year. The report also indicated that in China, with the deterioration of issues such as air pollution, there are 676,000 new cases and 565,000 deaths each year, ranking first in the world in the incidence and mortality rate of lung cancer. Non-small cell lung cancer (NSCLC) is the most common histological subtype, which accounts for 80-85% of all lung cancers^[1]^. In recent years, with the rapid development of molecular biology and translational medicine, the understanding of NSCLC has gone from the tissue level to the molecular level, and increasing cancer driver genes have been identified. Targeted therapies targeting these driver genes have replaced traditional chemotherapy as the standard treatment for advanced NSCLC in patients with these driver genes^[1]^.

The most representative drug of targeted therapy for NSCLC is epidermal growth factor receptor (EGFR) tyrosine kinase inhibitors (TKI). Clinical studies have shown that patients with EGFR mutations benefit significantly from TKIs (gefitinib, erlotinib, etc.) whose clinical efficacy is significantly superior to that of traditional chemotherapeutic agents and whose toxicity is relatively low. The current use of TKIs as first-line treatment in patients with EGFR mutations has become standard clinical treatment. However, a considerable number of patients with EGFR-negative cancer samples cannot benefit from the above treatment. Therefore, it is essential to find new therapeutic targets.

In 2007, Soda et al. identified a new fusion gene EML4-ALK in NSCLC. This fusion gene comprises portions of the echinoderm microtubule-associated protein-like 4 (EML4) gene and the anaplastic lymphoma kinase (ALK) gene. The total expression of EML4-ALK is 3.4% in unselected NSCLC patients and as high as 13.5% in some selected NSCLC populations. Due to the diversity of EML4-ALK fusion sites, at least a dozen different variants have been identified. EML4-ALK has carcinogenic activity in vitro and in vivo, which can be effectively blocked by ALK inhibitors. This indicates that it plays a key role in the development of lung cancer^[2-3]^.

Crizotinib is the first-generation oral ATP-competitive small-molecule ALK inhibitor. It inhibits c-Met and disrupts the signal transduction pathway, thereby inhibiting the ALK fusion gene and subsequent growth of tumor cells. The ALK fusion gene is found in 3–7% of all patients with NSCLC, and is often seen clinically in young, non-smoking patients with adenocarcinoma. ALK, EGFR and KRAS mutations are mutually exclusive. The advent of crizotinib significantly improved the prognosis of patients with this NSCLC subtype. The objective response rate (ORR) reached 60%. The progression-free survival (PFS) reached 8-10 months. And the overall survival (OS) was significantly prolonged. Although patients with ALK+ NSCLC benefit significantly, these patients often develop resistance to crizotinib within 1-2 years, and CNS recurrence and progression are relatively common^[4-6]^.

Around 1/3 of patients with crizotinib resistance develop point mutations in the ALK kinase region, resulting in the inability for crizotinib to inhibit enzyme activity. In other cases, the activation of alternative pathways allows tumor cells to undergo ALK-independent growth. It is generally agreed that point mutations pre-exist in a small number of subclones and are expanded after drug treatment. The activation of the alternative pathway is considered an adaptive mechanism^[7-8]^.

Point mutations have been extensively studied in vivo and in vitro. Like most first-generation inhibitors, crizotinib selectively avoids cancer cells with mutations in their housekeeping genes. Mutation in ALK housekeeping genes refers to the mutation of leucine at ALK1196 to methionine. The housekeeping gene is a key site for controlling the active site of ALK kinase. When the housekeeping gene is replaced by amino acids with large side chains, such as L1196M, it causes steric hindrance of crizotinib and hinders inhibitor binding. Therefore, drugs that are not affected by changes in the N-terminus or more effective inhibitors are needed to overcome these mutations^[7-8]^.

The emergence of second-generation ALK inhibitors effectively solved the problem of crizotinib resistance. Second-generation ALK inhibitors not only have activity against ALK-positive cancer cells, but also have activity against various ALK resistance mutations that have been identified. These drugs include: ceritinib (LDK-378), alectinib (CH5424804), and brigatinib (BRIGATINIB)^[9-12]^.

Ceritinib has demonstrated significant efficacy and sound safety in crizotinib-resistant or crizotinib-naive patients with ALK+ NSCLC. Ceritinib was marketed in the U.S. in 2014. However, after treatment with ceritinib for a period of time, chemoresistance recurs. Mutations in the ALK kinase domain at positions F1174 and G1202 have been observed in patients resistant to ceritinib.^[7-8]^.

Alectinib was jointly developed by Roche and Chugai and was marketed in the U.S. in Dec. 2015. Alectinib has shown good efficacy in patients with ALK+ NSCLC. However, after a period of treatment, chemoresistance was still observed. Mutations in the ALK kinase domain at positions I1171, F1174, and G1202 have been observed in patients resistant to alectinib^[7-8]^.

Brigatinib was developed by ARIAD and has been studied in phase I and II clinical trials. Brigatinib indicates comprehensive anti-tumor activity. All reported ALK mutations are sensitive to brigatinib, showing that brigatinib is able to overcome these mutations and can prevent or limit the development of drug resistance^[12,14-16]^.

At present, most clinical trials on second-generation ALK inhibitors highlight an interesting phenomenon: Second-generation ALK inhibitors showed a greater response rate in crizotinib-resistant patients than the expected rate of ALK-dependent resistance (approximately 30–40%). Second-generation ALK inhibitors can overcome ALK-dependent drug resistance and may also inhibit drug resistance caused by activation of alternative pathways. For example, as an effective EGFR target inhibitor, brigatinib can inhibit the EGFR alternative pathway, and ceritinib can inhibit the IGF1R alternative pathway^[7-8]^.

WX-0593 is a highly selective ALK inhibitor whose active ingredient is WX-0593. The new compound has been developed by Qilu Pharmaceutical Co., Ltd., and patents for the compound and the crystal structure have been applied. WX-0593 targets ALK and inhibits ALK activity in wild types with different fusion variants and in crizotinib-resistant mutants. At the cellular level, WX-0593 inhibits ALK activity and tumor cell proliferation in wild types with different fusion variants and in crizotinib-resistant mutant tumor cell lines. WX-0593 acts on downstream signaling pathways such as STAT3 by inhibiting ALK. *In vivo* tumor model studies have shown that WX-0593 is able to effectively inhibit the growth of crizotinib-sensitive and crizotinib-resistant ALK+ tumors, with superior efficacy to ceritinib and crizotinib. In addition, WX-0593 also inhibits kinase activity of EGFR^T790M/L858R^, an EGFR mutant, at the molecular level.

Drugs were clinically developed for the treatment in patients with locally advanced or metastatic ALK+ NSCLC, as well as metastatic ALK+ NSCLC with deteriorated conditions or who are intolerant after crizotinib therapy. According to the "Classification for Registration of Chemical Drugs" in the *Reform Scheme of the Classification System for Registration of Chemical Drugs*, this product belongs to Category 1, i.e. innovative new drugs not marketed anywhere in the world. Therefore, Phase I–III clinical trials are required.

## Preclinical Studies

WX-0593 targets ALK and inhibits ALK activity in wild types with different fusion variants and in crizotinib-resistant mutants. At the cellular level, WX-0593 inhibits ALK activity and tumor cell proliferation in wild types with different fusion variants and in crizotinib-resistant mutant tumor cell lines. WX-0593 acts on downstream signaling pathways such as STAT3 by inhibiting ALK. *In vivo* cancer models showed that WX-0593 can effectively inhibit the growth of crizotinib-sensitive and crizotinib-resistant ALK-positive cancers. At the molecular level, WX-0593 also inhibited enzyme activities of EGFR mutants EGFRT790M/L858R.

### Main Pharmacodynamic Studies

#### In Vitro Pharmacodynamic Studies

Results from the in vitro enzyme inhibition study showed that the inhibitory activity of the test compound WX-0593 for the main kinase target ALK wild type, ALK mutants (L1196M, C1156Y), and EGFR mutants EGFR L858R/T790M was comparable with the activity of brigatinib (with a difference in inhibitory activity within 3 times). The inhibitory activities were low for other serine, threonine, and tyrosine proteases, except for the EGFR wild type, within the tested concentration range. This indicates that WX-0593 is highly selective.

Results from the inhibitory study of 27 non-target kinases (MAP4K4, MINK, MST4, TAOK2, TINK, PDK1, CDK2, AUR2, PKA, CAMK2 alpha, JNK1, MAPKAPK2, CHK1, DYRK3, GSK3 beta, ABL, LCK, SRC, JAK2, EPHA2. The inhibition of KDR, FGFR1, P38 alpha, CK1 alpha, c-MET, INSR, and EGFR wild type) showed that the inhibitory activity of WX-0593 was basically the same as that of positive compound AP26113 (AP26113_003). Except for EGFR wild type (EGFR^wt^), WX-0593 and AP26113 have no significant inhibitory activity against other kinases, thus having good selectivity.

Results from the cell proliferation inhibition assay showed that the in vitro effects of WX-0593 were comparable with the reference compound AP26113. Both significantly inhibits the growth of Karpas299 (NPM-ALK), Ba/F3 (EML4-ALK-WT), Ba/F3 (EML4-ALK-L1196M), and Ba/F3 (EML4-ALK-C1156Y), somewhat inhibits the growth of NCI-H1975 (EGFR mutant L858R/T790M). The IC_50_ for Ba/F3 was significantly higher than that of ALK-related cells, showing sound selectivity and safety at the cellular level.

In the agonist screening assay, the activity percent at maximum dose of WX-0593 was all less than 50% for the 21 GPCR targets tested. The half effective concentration (EC_50_) was all greater than 133.3 μM (highest dose detected). In the antagonist screening assay, the IC_50_ of WX-0593 was all > 10 μM for targets of op-kappa, M1, and M2, and 0.5 μM for Eta target, showing slight activity. The IC_50_ for the remaining 17 GPCR targets was all greater than 111.1 μM (highest dose detected). Results showed that WX-0593 did not demonstrate significant agonistic or antagonistic effects against the 21 GPCR targets, indicating good safety for the off-targets tested.

#### In Vivo Pharmacodynamic Studies

The anti-tumor activity of WX-0593 was evaluated in two PDX models. Results showed that WX-0593 15 mg/kg, 10 mg/kg (D0–D7)/2.5 mg/kg (D8–D27), and 5 mg/kg had significant anti-tumor activity against ALK+ human lung cancer LU-01-0015 xenograft. WX-0593 was well-tolerated at all concentrations. There was no significant decrease in weight in all treatment arms. WX-0593 1 mg/kg, 2.5 mg/kg, and 5 mg/kg had good anti-tumor activity against ALK+ human lung cancer LU-01-0319 xenograft. There was no significant decrease in mean weight in all treatment arms.

A targeted therapy usually results in failure of treatment due to drug resistance. Use of crizotinib often leads to ALK mutations during the therapy, which then also gives rise to drug resistance. A new generation of ALK inhibitors can effectively overcome crizotinib resistance. In this study, PDX model of LU-01-0319R NSCLC was selected to investigate whether WX-0593 antagonizes crizotinib resistance. Results showed that this model was resistant to the first-generation ALK inhibitor crizotinib while second-generation inhibitors ceritinib 10/20 mg/kg and brigatinib 10 mg/kg had significant effects against crizotinib-induced resistance. WX-0593 2.5 mg/kg, 5 mg/kg, and 10 mg/kg had significant anti-tumor effects. There was no significant decrease in weight in all treatment arms.

NCI-H3122 (EML4-ALK fusion gene positive) is an ALK wild type human NSCLC. Results from the CDX model showed that drug exposure of WX-0593 2.5, 5, and 10 m/kg in plasma, tumor, brain, and lung tissue all increased with dose, and treatment efficacy also increased with dose. A significant increase in drug exposure in tumors is the material basis for significantly improving efficacy. At equivalent doses, the exposure of WX-0593 in tumor, brain, and lung tissue at 8 h was significantly higher than at 1 h after dosing; At 1h and 8 h after dosing, the drug concentrations of WX-0593 were all lungs > tumor > brain across all dose arms. Higher drug concentration in the lungs provides the material basis for the treatment of lung cancer.

Efficacy was comparable between WX-0593 and brigatinib at doses of 5 mg/kg. Plasma drug exposure was also comparable. The drug exposure of WX-0593 in tumor tissue was 2 times greater than brigatinib. At 8 h after dosing, the drug exposure of WX-0593 in the brain was also approximately 2 times greater than brigatinib. At 1h after dosing, the drug exposure of WX-0593 in the lungs was also approximately 2 times greater than brigatinib. At 8 h after dosing, the drug exposure of WX-0593 in the lungs was approximately 3 times greater than brigatinib, suggesting a higher pulmonary to systemic blood flow ratio. Since the tumor in the animal model was inoculated subcutaneously rather than in the lungs, it is concluded that the inhibitory effect of WX-0593 is comparable to brigatinib in brain cancer patients, and comparable or superior to brigatinib in lung cancer patients.

In summary, this study has confirmed the in vivo inhibitory effects of test compound WX-0593 on ALK targets through 3 ALK+ PDX models. In addition, WX-0593 is able to overcome crizotinib resistance without significant toxicity and visible adverse reactions.

### Pharmacokinetic study

#### Absorption

Rats and cynomolgus monkeys were selected for the absorption studies based on the "Guidance on the non-clinical pharmacokinetics of medicinal products" (CFDA, 2014) and the in vitro study of species differences in liver microsomes and hepatocyte metabolism.

- Absorption study in Sprague Dawley (SD) rats

After a single IV dose of WX-0593 2 mg/kg in SD rats, plasma clearance (CL) was 22.2 ± 4.67 mL/min/kg (40.3% of hepatic blood flow in rats), apparent volume of distribution (Vdss) was 5.23 ± 0.942 L/kg, mean residence time (MRT_0-inf_) was 3.96 ± 0.355 h, mean plasma elimination half-life T_1/2_ was 3.58 ± 0.652 h, and AUC_0-inf_ was 1560 ± 348 ng•h/mL.

After oral gavage of WX-0593 2–24 mg/kg in SD rats, the T_max_ was between 2.08–2.75 h and mean plasma terminal half-life was between 3.14–3.70 h. After dose correction, the absolute bioavailability in SD rats after a single oral gavage of 2, 8, and 24 mg/kg was 36.9%, 64.7%, and 69.6%, respectively, based on the mean AUC_0-inf_. Within the dose range of 2–24 mg/kg, the exposure of WX-0593 increased with dose, linearly between 8–24 mg/kg. However, the increase in exposure was higher than the proportion of dose increase between 2–8 mg/kg, showing non-linear absorption.

There were no significant gender differences in systemic exposure (AUC and C_0_/C_max_) of SD rats administered at 2 mg/kg via intravenous injection and 2, 8, and 24 mg/kg via oral gavage (exposure ratios of male to female were 1.13–1.37). After oral gavage of WX-0593 at 8 mg/kg for 7 consecutive days, no significant drug accumulation was observed on Days 1 and 7.

- Absorption study in cynomolgus monkeys

After a single IV dose of WX-0593 0.5 mg/kg in female and male cynomolgus monkeys, mean plasma clearance (CL) was 26.8 ± 6.82 mL/min/kg, apparent volume of distribution (Vdss) was 7.70 ± 1.24 L/kg, mean residence time (MRT_0-inf_) was 5.03 ± 138 h, mean plasma elimination half-life T_1/2_ was 4.37 ± 2.38 h, and AUC_0-24h_ was 323 ± 91.1 ng•h/mL.

After oral gavage of WX-0593 0.5–8 mg/kg in female and male cynomolgus monkeys, the mean T_max_ was between 2.00–3.33 h and mean plasma terminal half-life was between 3.29–8.14 h. Calculated based on the mean AUC_0-24 h_ after dose calibration, the absolute bioavailabilities of single oral doses of 0.5, 2, and 8 mg/kg in cynomolgus monkeys were 37.5%, 46.6%, and 74.7%, respectively.

Following dose increase from 0.5 mg/kg to 2 mg/kg, the systemic exposure, AUC_0-24 h_, and C_max_, in female and male cynomolgus monkeys increased with increasing doses. Following dose increase from 2 mg/kg to 8 mg/kg, the systemic exposure, AUC_0-24 h_ and C_max_, in female and male cynomolgus monkeys increased proportionately with the dose. However, the increase of systemic exposure, AUC_0-24 h_ and C_max_, in female cynomolgus monkeys was higher than that of the dose, exhibiting a certain extent of non-linear absorption characteristics. In general, within the range of 0.5 mg/kg–8 mg/kg, the systemic exposure in male cynomolgus monkeys increased proportionately with the dose, while increased slightly faster than the dose in female cynomolgus monkeys.

After oral administration at 2 mg/kg for 7 consecutive days, no significant drug accumulation was observed on Day 7 comparing the systemic exposure, AUC_0-24 h_, of WX-0593 in cynomolgus monkeys to that on Day 1. There were no significant gender differences in the systemic exposure, AUC_0-24 h_ and C_max_, of WX-0593 in cynomolgus monkeys at all the oral doses.

- Caco-2 cell monolayer permeability assay

The objective of this study was to determine the bidirectional permeability and efflux ratio (ER) of the test article WX-0593 using the Caco-2 cell monolayer model. Results showed that WX-0593 is very likely to be a substrate of efflux transporters. At low doses (0.5 and 5 µM), WX-0593 showed low permeability in Caco-2 cells; when the dose was increased to 50 µM, WX-0593 permeability in Caco-2 cells increased, and characteristics consistent with saturation of efflux transporters by substrates were observed, demonstrating high permeability.

#### Distribution

- Plasma protein binding rate

The objective of this study is to evaluate the plasma protein binding rate of WX-0593 in CD-1 mice, SD rats, beagles, cynomolgus monkeys, and humans using equilibrium dialysis. Results showed that except for a high protein binding rate (≥ 95.0 and ≤ 99.0) at a concentration of 0.2 μM in rat plasma, WX-0593 showed moderate protein binding (≥ 50.0 and < 95.0) at the 3 test concentrations (0.2, 2, and 10 μM) in all five species described above. As the test concentration increased, protein binding of WX-0593_013 in rat plasma decreased gradually, exhibiting some concentration dependence. Concentration dependence was not observed in the other species.

- Distribution in rat tissue

No significant difference in total radioactivity between tissues as well as whole blood and plasma was found at 0.5, 3, 24, and 72 hours after a single oral dose of [^14^C]WX-0593 8 mg/kg/100 µCi/kg in SD rats.

The experimental results showed that the C_max_ of total radioactivity in the tested tissues was reached at 3 hours after drug administration, except for the oesophagus, stomach wall and intestinal wall, in which the C_max_ was reached at 0.5 h after oral drug administration, and the testis and epididymis, in which the C_max_ was reached at 24 h after drug administration. At 0.5 h after drug administration, the total radioactivity was mainly distributed in the gastrointestinal tract, liver, oesophagus, and adrenal glands. The mean total radioactivity of intestinal wall (42737 ng Eq./g), stomach wall (28265 ng Eq./g), liver (15160 ng Eq./g), oesophagus (13803 ng Eq./g), and adrenal glands (13334 ng Eq./g) were significantly higher than that of plasma (1522 ng Eq./g) at the same time point, which were 28.1 times, 18.6 times, 9.96 times, 9.07 times and 8.76 times of that of plasma, respectively. The distribution of total radioactivity in other tissues in descending order was lungs, kidneys, spleen, pancreas, lymph nodes, heart, uterus and ovaries, plasma, thymus, prostate, whole blood, skeletal muscles, skin, testis and epididymis, and whole brain. At 3 h after drug administration, the C_max_ of total radioactivity in most tissues was reached and was significantly higher than that of plasma (2062 ng Eq./g) at the same time point, which was 1.60 times (prostate) to 15.1 times (intestinal wall) of that of plasma. Only the total radioactivity of skeletal muscles (1417 ng Eq./g), whole blood (1366 ng Eq./g), body fat (1079 ng Eq./g), testis and epididymis (619 ng Eq./g), and whole brain (114 ng Eq./g) were lower than that of plasma at the same time point. At 24 h after drug administration, the total radioactivity of most tested tissues was significantly lowered, of which the distribution concentration of skeletal muscles, body fat, whole brain, and whole blood were below the lower limit of quantitation. At the last sampling time point (72 h), most total radioactivity had been cleared from the body and only the spleen, adrenal glands, liver, testis and epididymis, kidneys, lymph nodes, uterus and ovaries, intestinal wall, lungs, and thymus had been traced small amount of radioactivity. The mean total radioactivity in those tissues were 1883 ng Eq./g, 1296 ng Eq./g, 922 ng Eq./g, 504 ng Eq./g, 438 ng Eq./g, 292 ng Eq./g, 187 ng Eq./g, 129 ng Eq./g, 103 ng Eq./g, and 95.8 ng Eq./g, respectively.

#### Metabolism

- In vitro metabolic stability assay in liver microsomes

The objective of this study is to evaluate the metabolic stability of tested drug WX-0593 in CD-1 mice, SD rats, beagles, cynomolgus monkeys, and human liver microsomes. Results showed that the elimination half-life of WX-0593 co-incubated with the liver microsomes solution of CD-1 mice, SD rats, beagles, cynomolgus monkeys and humans was 105.0, > 145, 135.9, > 145, and > 145 minutes, respectively, which corresponds to a CLint (liver) of 52.3, < 17.3, 14.7, < 13.0, and < 9.5 mL/min/kg. The above results showed that WX-0593 was metabolized at a slow rate in SD rats, cynomolgus monkeys, and human liver microsomes, and metabolized at a moderate rate in CD-1 mice and beagle liver microsomes.

- Metabolite identification in liver microsomes

Metabolites of WX-0593 in liver microsomes incubation systems of humans, cynomolgus monkeys, beagles, rats and mice were analyzed and identified by UPLC/Q-TOF-MS. A total of 5 metabolites were detected and identified: a mono-oxidation metabolite, M1 (MW = 584.24); an N-demethylation metabolite, M2 (MW = 554.23); a dehydrogenation metabolite, M3 (MW = 566.23); an O-demethylation and dehydration metabolite, M4 (MW = 536.22); a mono-oxidation metabolite, M5 (MW = 584.24). M1, M2, and M5 were detected in human microsomes; M1, M2, and M5 were detected in monkey microsomes; M2 and M5 were detected in dog microsomes; M1, M2, M4, and M5 were detected in rat microsomes; and M1, M2, M3, and M5 were detected in mice microsomes. Based on the type of metabolites, it is speculated that WX-0593 is mainly metabolized by oxidation, dehydrogenation, N-demethylation and O-demethylation in liver microsomes incubation system.

According to the ultraviolet absorption peak area, M2 (7.8%) and M5 (7.1%) were considered to be the main metabolites in human microsomes incubation system, M1 (13.1%), M2 (7.5%), and M5 (9.0%) in monkey microsome incubation system, M5 (39.4%) in dog microsome incubation system, M5 (5.1%) in rat microsome incubation system, and M2 (21.2%) and M3 (10.9%) in mice microsome incubation system. There were no detected metabolites that were specific to liver microsomes of human as compared to the animal species. All the metabolites could be detected in animal species.

- Metabolite identification in hepatocytes

Only M5 was detected in human, monkey, and dog hepatocytes incubation system (M1, M2, M3, and M4 were detected in microsomes). M5 was identified as a mono-oxidation metabolite (MW = 584.24). The UV peak area % of M5 was 2.26%, 0.96%, and 8.48% in human, monkey, and dog hepatocyte incubation systems, respectively. No relevant metabolites were detected in the hepatocyte incubation systems of rats and mice. The main metabolic pathway of WX-0593 in hepatocytes is oxidation.

There were no detected metabolites that were specific to human hepatocytes as compared to the animal species. All the metabolites could be detected in animal species.

- Metabolite identification in SD rat plasma

Under current experimental conditions, a total of 2 metabolites in addition to the parent drug were detected in rat plasma: a demethylation metabolite, M2 (MW = 554.23, P - CH2) and a mono-oxidation and dehydrogenation product, M6 (MW = 582.23, P + O - 2H). Based on the % peak area from mass spectrometry, the drug exists primarily as the parent drug in the plasma of both male and female rats (97.24% in females and 96.81% in males). Metabolites M2 (2.76% in females and 2.88% in males) and M6 (1.49% in females and 0.31% in males) were both secondary metabolites. Based on the types of metabolites, it is speculated that WX-0593 is mainly metabolized through demethylation, oxidation, and dehydrogenation after oral administration in rats. There was no significant difference in metabolites between female and male rats.

- Metabolite identification in cynomolgus monkey plasma

Under current experimental conditions, 1 metabolite in addition to the parent drug was detected in monkey plasma: a demethylation metabolite, M2 (MW = 554.23, P - CH_2_). Based on the % peak area from mass spectrometry, the drug exists primarily as the parent drug in the plasma of both male and female monkeys (99.06% in females and 99.47% in males). Metabolite M2 (0.94% in females and 0.53% in males) was a secondary metabolite. Based on the types of metabolites, it is speculated that WX-0593 is mainly metabolized through demethylation after oral administration in monkeys. There was no significant difference in metabolites between female and male monkeys.

- Metabolic enzyme phenotyping of WX-0593

Experimental results of specific chemical inhibitors and recombinant human cytochrome P450 enzyme metabolism showed that metabolites M1, M2 and M5 of WX-0593 were primarily metabolized by CYP3A4. Other isoenzymes (CYP1A2, CYP2B6, CYP2C8, CYP2C9, CYP2C19 and CYP2D6) played a secondary or insignificant role in the formation of M1, M2, and M5.

#### Excretion

- Excretion and mass balance

After a single oral administration of [^14^C]WX-0593 8 mg/100 µCi/kg in female and male rats, overall or bile-duct cannulation (BDC), results showed that total radioactivity was similar between females and males rats based on the rate and amount of excretion. Therefore, the discussion below uses the mean excretion of female and male rats.

The mean radioactivity recovery rate was 96.71% within 0-168 hours after a single oral administration in female and male rats, among which 9.36% of the dose was excreted in urine, 85.84% in feces, and 1.51% in cage rinses and washes.

The mean radioactivity recovery rate was 94.75% within 0-72 hours after a single oral administration in female and male BDC rats, among which 30.56% of the dose was excreted in bile, 30.73% in urine, 32.04% in feces, and 1.43% in cage rinses and washes.

### Preclinical safety studies

#### General pharmacology

The inhibition of hERG currents by compound WX-0593 at the maximum tested dose of 30 μM was 58.06% ± 4.63%. The IC_50_ was 20.05 μM and the slope was 0.95, outperforming those of the drugs with similar targets: 1.1 μM of crizotinib, 0.4 μM of ceritinib, and 0.45 μM of alectinib. Therefore, WX-0593 has a low risk of cardiotoxicity. There were no effects on the CNS and respiratory system in rats at doses of 3, 10, and 30 mg/kg. However, WX-0593 10 and 30 mg/kg may result in benign respiratory rate and decreases in minute ventilation in female rats. There were no effects on the cardiovascular system, blood pressure, ECG parameters or waveforms in monkeys at doses of 5, 10, and 25 mg/kg; 10 and 25 mg/kg may result in mild decreases in heart rate (within the normal range) and PR prolongation and QTc shortening associated with bradycardia within the normal range (no statistical difference when compared to the control arm). No cardiovascular system adverse reactions related to the test product were found. The human equivalent dose (using 60 kg) is 3636 mg/person in CNS, 3636 mg/person in male respiratory system and 363.6 mg/person in female respiratory system, and 607 mg/person in cardiovascular system.

#### Acute toxicity study

In a single-dose toxicity study (acute toxicity), the maximum tolerated dose (MTD) was 50 mg/kg in female animals and 200 mg/kg in males. The murine effective dose was 2.5 mg/kg. The above doses are 10 and 40 times of the effective dose, respectively. The MTD in cynomolgus monkeys was 50 mg/kg after a single oral dose. At this dose, AUC_0-24h_ and C_max_ of WX-0593 in male animals were 35000 h·ng/mL and 2180 ng/mL, respectively. AUC_0-24h_ and C_max_ of WX-0593 in female animals were 50600 h·ng/mL and 4010 ng/mL, respectively. The above dose is 20 times of the effective dose in nude mice. At this dose, AUC_0-24h_ in nude mice was 3700 h·ng/mL. Drug exposure is 9.5 and 13.7 times of the effective dose, respectively.

#### Chronic toxicity study

In a repeated-dose toxicity study (chronic toxicity), the NOAEL was 10 mg/kg/day in rats after 28 days of continuous administration by oral gavage. The severely toxic dose in 10% of the animals (STD10) in rodents was > 30 mg/kg. At a dose of 10 mg/kg/day, AUC_0-24h_ (Day 28) was 16800 h·ng/mL in males and 17300 h·ng/mL in females, which is 4.5 times and 4.7 times of the exposure at the effective dose in mice (2.5 mg/kg, AUC_0-24h_: 3700 h·ng/mL). At a dose of 30 mg/kg/day, AUC_0-24h_ (Day 28) was 27100 h·ng/mL in males and 42400 h·ng/mL in females, which is 7.3 times and 11.5 times of the exposure at the effective dose in nude mice (2.5 mg/kg, AUC_0-24h_: 3700 h·ng/mL).

The NOAEL was 3 mg/kg/day in cynomolgus monkeys after 28 days of continuous administration by oral gavage. The HNSTD was greater than 10 mg/kg/day, which is 4.8 times and 16 times of the effective dose in nude mice.

#### Mutagenicity study

The results from mutagenicity study showed that WX-0593 had no mutagenic activity, no DNA damage- and chromosomal aberration-inducing activity in mice, and no cell chromosomal aberration-inducing activity.

#### Reproductive toxicity studies

Results from a reproductive toxicity study showed that the NOAEL of rat fertility and early embryo developmental toxicity was 10 mg/kg, and the NOAEL of embryo-fetal developmental toxicity was 10 mg/kg, which was 8 times of the effective dose in nude mice.

# OVERALL TRIAL DESIGN

First, single dose and repeated dose escalation studies will be carried out in patients with ALK-positive or ROS1-positive advanced solid tumors. The pharmacokinetic study for single and repeated dose will be carried out simultaneously with the tolerability study and antineoplastic activity will be preliminarily evaluated.

Subsequently, the population expansion study will be conducted based on the earlier study. The target dose will be selected and target tumor patients with measurable lesions will be enrolled. ORR (assessed using RECIST 1.1) is deemed to be the primary efficacy marker and the efficacy and safety of this product will be assessed.

When the dose escalation study has reached a certain stage, a suitable dose will be selected for a food effects study in healthy subjects. Subsequently, studies on mass balance and interactions with other drugs will be carried out in healthy subjects.

In subsequent studies, head-to-head comparison with marketed doses of the ALK inhibitor crizotinib in patients with ALK-positive advanced or metastatic non-small cell lung cancer can be carried out to assess the safety and efficacy of the drug.

In the phase I clinical trial, an exploratory study will be simultaneously conducted to explore possible drug resistance genes for crizotinib, WX-0593, or other ALK inhibitors, to explore possible drug resistance genes when ROS1-positive NSCLC patients are treated with crizotinib or WX-0593, to measure WX-0593 concentration in cerebrospinal fluid and preliminarily assess the efficacy of the investigational product in patients with brain metastases and its drug concentration basis, and to assess the OS of patients treated with WX-0593.

# TRIAL OBJECTIVE

## Study Objectives

### Dose escalation phase

**Primary objectives:**

To observe the safety and tolerability and to determine the dose-limiting toxicity (DLT), maximum tolerated dose (MTD), and the subsequent recommended dose of WX-0593 oral tablets in patients with ALK-positive (translocation/over-expression) or ROS1-positive advanced solid tumors.

**Secondary objectives:**

To observe and analyze the pharmacokinetic characteristics of WX-0593 tablets in patients with ALK-positive (translocation/over-expression) or ROS1-positive advanced solid tumors; to preliminarily observe the clinical efficacy of WX-0593 tablets in patients with ALK-positive (translocation/over-expression) or ROS1-positive advanced solid tumors.

### Population expansion phase

**Primary objectives:**

Preliminary evaluation of the efficacy of WX-0593 tablets in patients with ALK-positive (translocation/over-expression) or ROS1-positive advanced NSCLC patients.

**Secondary objectives:**

To observe the safety of WX-0593 tablets in patients with ALK-positive (translocation/over-expression) or ROS1-positive advanced NSCLC.

### Exploratory study

- To explore disease progression-related ALK gene mutation, ROS1 gene mutation, and other molecular mechanisms of drug resistance after treatment with crizotinib, WX-0593, or other ALK inhibitors.
- To measure WX-0593 concentration in cerebrospinal fluid and preliminarily assess the efficacy of the investigational product in patients with brain metastases and its drug concentration basis.
- To assess the overall survival (OS) of patients treated with WX-0593.

## Study endpoints

### Dose escalation phase

**Primary endpoints:**

- To determine the MTD, DLT, and subsequent recommended dose(s).

Incidence of adverse events, including adverse events (AEs), serious adverse events (SAEs), and treatment-emergent adverse events (TEAEs). Causality is determined by the investigator.

**Secondary endpoints:**

- Pharmacokinetic (PK) parameters: T_max_, C_max_, AUC, and t_1/2_ after a single dose; C_ssmin_, C_ssmax_, C_ss-av_, t_1/2_, AUC_ss_, DF, V_z_, and CLs after repeated doses.
- Preliminary efficacy: The evaluation indicators include objective response rate (ORR), progression-free survival (PFS), disease control rate (DCR), time to progression (TTP), duration of response (DOR) and intracranial ORR.

### Population expansion phase

**Primary endpoints**: ORR

**Secondary endpoints:**

- PFS, DCR, TTP, DOR, and CNS objective response rate.
- Incidence of adverse events, including AEs, SAEs, and TEAEs. Causality is determined by the investigator.

### Exploratory study

- The relationship between ALK gene mutation, ROS1 gene mutation, and other molecular changes in blood and tumor tissues and antineoplastic activity of WX-0593.
- (Some patients with brain metastases will be selected) to measure WX-0593 concentration in cerebrospinal fluid and preliminarily assess the efficacy of the investigational product in patients with brain metastases and its drug concentration basis.
- OS

# Study design

- Dose escalation phase

This study is a single-arm, open-label, multi-center clinical trial in China.

Patients with ALK-positive or ROS1-positive advanced solid tumors were planned to be enrolled in the dose-escalation trial consisting of a single dose and repeated doses. The pharmacokinetic study at a single dose and repeated doses was carried out simultaneously with the tolerability study. PK blood sampling and safety observation were carried out after administration of a single dose. Continuous administration began on the 5th day after a single dose was administered. PK blood sampling was carried out on the 21st day of continuous administration. At the end of the tolerability study for each arm, provided that there is good safety and the patients benefit from the treatment as judged by the investigator, 3–6 dose arms will be selected and additional patients will be enrolled for single-dose and repeated-dose PK studies. At least 8 subjects in each arm should be included in the PK studies (ensuring that there are at least 8 evaluable subjects in each arm).

The starting dose in the dose escalation phase is 30 mg, and safety and DLT will be observed. The proposed dose escalation is 30 mg, 60 mg, 90 mg, 120 mg, 180 mg, 240 mg, 300 mg, and 360 mg. During the trial, dose modification may be performed by the investigator and the sponsor according to preliminary data. If DLT was not observed at the highest dose arm, whether to continue the dose-escalation trial should be decided by the investigator and the sponsor. The 3+3 dose escalation principle was adhered to and 39–60 patients were to be enrolled.

- Population expansion phase

This study is a single-arm, open-label, multi-center clinical trial in China. Patients with ALK-positive (translocation/over-expression) or ROS1-positive advanced NSCLC will be enrolled for a repeated-dose efficacy and safety study.

After the maximum tolerated dose (MTD) is determined in the dose escalation phase, 120 mg QD and 180 mg QD are selected for the population expansion phase study to determine the optimal target dose for subsequent studies. However, corresponding adjustments during the study period can be jointly determined by the investigator and sponsor based on the results of previous clinical studies. Before administration of 120 mg QD or 180 mg QD in subjects, it is recommended that a run-in dose of 60 mg QD be administered for 7 continuous days. If the subject is tolerable, the dose is escalated to 120 mg QD or 180 mg QD and administered until disease progression, unacceptable toxicity, voluntary withdrawal, loss to follow-up, start of another anti-cancer therapy, death, or end of study (whichever occurs first). After efficacy and safety comparison data between two arms is obtained at the population expansion phase, the optimal target recommended dose will be determined for subsequent phase II studies. 30–70 patients were planned to be enrolled per arm and 60–140 patients were planned to be enrolled in total.

- Exploratory study
- Peripheral blood samples will be collected and tumor samples will be collected as much as possible from ALK-positive or ROS1-positive patients who resistant to crizotinib or other ALK inhibitors for an exploratory study on drug resistance genes.
- During the trial, peripheral blood samples will be collected and tumor samples will be collected as much as possible from subjects with progressive disease for an explanatory study on drug resistance genes.
- With regards to patients with brain metastases, a few of such patients can be selected after integrated judgment by the investigator and after consent was obtained from the patients. Cerebrospinal fluid will be collected at the first imaging assessment at the end of 6 continuous weeks of treatment in the 2nd cycle or 3 h (+ 0.5 h) after dosing in other stable states. Drug concentrations in the cerebrospinal fluid will be compared with plasma drug concentrations (blood samples should be simultaneously collected) to determine the relationship between efficacy and concentration of WX-0593 in brain metastasis patients.
- To assess the overall survival (OS) of patients treated with WX-0593.

## Determination of starting dose

### Preclinical study data

Repeated administration for 28 days was carried out. 3, 10, and 30 mg/kg were administered to rats. 1, 3, and 10 mg/kg were administered to cynomolgus monkeys. The results show that the confirmed no-observed-adverse-effect-level (NOAEL) for rats and cynomolgus monkeys in the 28-day repeated-dose toxicity studies is 10 mg/kg and 3 mg/kg, respectively. The STD10 of rats (dose resulting in severe toxicity in 10% of rodents) is greater than 30 mg/kg. The highest non-severely toxic dose (HNSTD) of cynomolgus monkeys is greater than 10 mg/kg.

### Basis of guidelines

According to "Technical Guidelines for Clinical Trials for Anti-tumor Drugs", for non-cytotoxic drugs that have low toxicity, 1/5 or a higher portion of the NOAEL for non-rodent animals in non-clinical studies can be used to calculate the starting single dose for the Phase I clinical trial.

The 4-week chronic toxicity study of WX-0593 in cynomolgus monkeys shows that NOAEL is 1 mg/kg.

The human equivalent dose (HED) = 1 mg/kg÷3.1÷5×60 kg/person = 12 mg/person

### Basis of FDA/ICH guidelines

According to FDA/ICH: Guidance for Industry S9 Nonclinical Evaluation for Anticancer Pharmaceuticals, the aim of selecting a starting dose is to find a dose that is both safe and pharmacologically effective. All pre-clinical data (such as pharmacokinetics, pharmacodynamics, and toxicological data) should be used for scientific validation of the starting dose. The following methods should be used for selection: For most systemic small molecule preparations, the interspecies extrapolation of animal doses to a HED should be based on the standardized body surface area. For small molecules and biologics, interspecies extrapolation based on weight, AUC, or other exposure parameters is also reasonable^[13]^.

For many small molecular antineoplastic drugs, a universal method is to use 1/10 of the STD10 of rodents as the starting dose. For non-rodent animals, 1/6 of its HNSTD is used as the starting dose;

In the following, only body weight was used for conversion to HED:

Rat STD10 greater than 30 mg/kg and cynomolgus monkeys HNSTD greater than 10 mg/kg for WX-0593

Based on 1/10 of rat STD10: 30 mg/kg÷6.2÷10×60 kg/person = 29 mg/person

Based on 1/6 of cynomolgus monkeys HNSTD: 10 mg/kg÷3.1÷6×60 kg/person = 32 mg/person

### Situation of similar drugs

Comparison of non-clinical toxicological dose and clinical starting dose for escalation of similar drugs^[3-9,14-16]^

|  | Non-clinical toxicological dose (mg/kg) | Clinical starting dose for escalation |
| --- | --- | --- |
| BRIGATINIB | Rats: 3, 10, 30 | Patients: 30, 60, 90, 120, 180, 240, 300 |
|  | Cynomolgus monkeys: none |  |
| Ceritinib | Rats: 7.5, 25, 75, 50 | Patients: 50, 100, 200, 300, 400, 500, 600, 700, and 750 mg/person/day |
|  | Cynomolgus monkeys: 3, 10, 30 |  |
| Alectinib | Rats: 3, 10, 30 | Patients: (1) 20, 40, 80, 160, 240, 300, QD  (2) 20, 40, 80, 160, 240, 300 mg, BID |
|  | Cynomolgus monkeys: 1, 3, 10 |  |

### Use of allometric scaling model and gastroplus software for prediction of effective dose in humans

Use the allometric scaling model to predict the effective dose in humans:

The effective dose in humans is calculated by using the formula Dose·F= Cl·AUC:

According to the method based purely on weight, CL is 57.4L/h and the effective dose in humans is calculated to be 26.6 mg.

According to the MLP correction method, CL is 27.9 L/h, and the effective dose in humans is calculated to be approximately 12.9 mg.

Use the Gastroplus software to predict the effective dose in humans:

Use the formula Dose·F= Cl·AUC, in which CL = 18.5 L/h and F = 44.2%, and the effective dose in humans is calculated to be approximately 11 mg.

**Conclusion:** According to currently available preclinical data and simultaneous referencing of the preclinical toxicology and clinical escalation doses for the template drug, brigatinib, an **expected starting dose** **of 30 mg** will be used for the single dose study in humans.

## Dose Arm Setting And Dose Escalation

### Dose arm setup

After taking into consideration pre-clinical data and the dose escalation data for the template drug, the modified Fibonacci sequence was used for dose escalation, and doses were set as follows:

| **Group** | **1** | **2** | **3** | **4** | **5** | **6** | **7** | **8** |
| --- | --- | --- | --- | --- | --- | --- | --- | --- |
| **Dose (mg)** | 30 | 60 | 90 | 120 | 180 | 240 | 300 | 360 |
| **Dose increment ratio** | / | 100% | 50% | 33% | 50% | 33% | 25% | 20% |
| **No. of enrolled patients** | 3–6 | 3–6 | 3–6 | 3–6 | 3–6 | 3–6 | 3–6 | 3–6 |

### Principles for dose escalation

A 3+3 dose-escalation design as follows will be used:

- Three subjects will be enrolled in each dose arm. Subjects will be observed for 4 days after a single dose on Day 1. Then, repeated dosing will be carried out daily from Day 5 onwards. Dose will be escalated to the next dose group after the 3rd subject in the dose arm completed 21 days of repeated dosing and no DLT is observed.
- If 2 or more DLTs occur among the 3 subjects in a certain dose arm after a single dose is administered and in the first 21 days of repeated dosing, dose escalation should be stopped.
- If DLT is observed in 1 subject in a dose arm within 21 days after single dosing and repeated dosing, then another 3 subjects should be enrolled into that dose arm. Dose escalation should be stopped if DLT is observed in 1 or more of these 3 subjects. Dose escalation will continue to the next dose arm if no DLT is observed.
- If DLT is not observed for patients in all dose arms, then the original dose would be maintained until disease progression. If DLT is not observed at the highest dose arm, whether to continue the dose-escalation would be decided by the investigator and the sponsor.

## Determination criteria for dose-limiting toxicity (DLT) and maximum tolerated dose (MTD)

Based on the clinical development experiences for similar drugs (crizotinib, ceritinib, alectinib, and brigatinib), possible toxic side effects that may occur at the dose escalation phase mainly include^[3-9,14-16]^:

Crizotinib: The most common adverse reactions are usually Grades 1 and 2: visual disturbances, nausea, diarrhea, constipation, vomiting, and peripheral edema. The most common Grade 3 and 4 adverse reactions are neutropenia, ALT increased, hypophosphatemia, lymphocytopenia, pneumonitis, fatigue, and prolonged QT interval.

Ceritinib: Grades 1 and 2 adverse reactions are most common, including nausea (82%), diarrhea (75%), vomiting (65%), fatigue (47%), and ALT increased (35%); the most common Grade 3 and 4 adverse reactions are ALT increased (21%), diarrhea (7%), lipase increased (7%), interstitial pneumonia (4%), hyperglycemia (13%), and prolonged QT interval (3%).

Alectinib: Grades 1 and 2 adverse reactions are most common, including myalgia (17%), constipation (15%), fatigue (14%), and asthenia (11%). The most common Grade 3 and 4 adverse reactions are gamma-glutamyl transferase elevation, neutropenia, hypophosphatemia, interstitial pneumonia (0.4%), and bradycardia (7.5%).

Brigatinib: The most common adverse reactions of brigatinib are usually Grades 1 and 2, including nausea (45%), diarrhea (36%), fatigue (36%), coughing (26%), and headache (26%). Two DLTs occurred in the tolerability study: One Grade 3 ALT increased, one Grade 4 dyspnea and Grade 3 hypoxia. Serious adverse events (SAEs) that occurred in more than 2 patients included pneumonia (7%), coughing (4%), dyspnea (4%), hypoxia (4%), and pleural effusion (4%)

Particular attention should be paid to similar phenomena for this drug during dose escalation.

**Definition of DLT**

Based on the preclinical study results for this product and by referencing the clinical trial materials for similar drugs (ceritinib and brigatinib), the DLT principles are confirmed (refer to NCI-CTC AE 4.03) and proposed as follows (after single dose administration and first cycle of repeated dosing):

**Occurrence of the following grades of drug-related toxicity after subjects have received treatment:**

- Hematological toxicity: Grade 4 thrombocytopenia or Grade 3 thrombocytopenia with significant hemorrhagic diathesis, Grade 4 neutropenia that persists for ≥ 3 days or Grade 3 neutropenia with fever ≥ 38.3℃, and other Grade 4 hematological toxicity.
- Non-hematological toxicity: Grade 3 and above non-hematological toxicity (excluding transient electrolyte abnormality and diarrhea, nausea, and vomiting that could resolve to Grade 2 after best supportive treatment); Grade 2 and above cardiac insufficiency.

**Definition of MTD**

The dose-escalation process is terminated if DLT is observed in more than 1/3 of the patients. The previous dose is the maximum tolerated dose (i.e. MTD).

## Pharmacokinetic study

At the end of the tolerability study for each arm, provided that there is good safety and the patients benefit from the treatment as judged by the investigator, 3–6 dose arms will be selected and additional patients will be enrolled for single-dose and repeated-dose PK studies. At least 8 subjects in each arm should be included in the PK studies (ensuring that there are at least 8 evaluable subjects in each arm). Blood collection time points will be adjusted and set based on the preliminary trial results (see Section 10 for details).

Sample collection: Single dosing and repeated dosing plasma samples will be used for pharmacokinetic parameter analysis;

Test method: The plasma drug concentration will be determined using LC-MS.

# SUBJECT SELECTION

The dose escalation phase is carried out in patients with ALK-positive or ROS1-positive advanced solid tumors. The population expansion phase is carried out in patients with ALK-positive NSCLC.

## Inclusion Criteria

1. Above 18 years old;
2. Gender: Male or female;
3. ECOG PS score 0/1;
4. Expected survival no less than 12 weeks;
5. Patients should have at least one measurable lesion (RECIST 1.1); Lesions previously treated with radiotherapy can be considered target lesions only if there is clear evidence of progression after radiotherapy;
6. The following patients who are proven to be ALK-positive (translocation/over-expression) or ROS1-positive by histopathological or cytological tests in Grade III hospitals (Subjects in the population expansion phase are ALK- or ROS1-positive advanced NSCLC patients):

- Patients with advanced malignant tumors (such as NSCLC, lymphoma, inflammatory myofibroblastic tumor, etc.) who failed standard treatment [such as resistance to ALK inhibitors (excluding brigatinib and other structurally similar drugs), chemotherapy failure, etc.];
- Advanced NSCLC patients who cannot receive or tolerate chemotherapy;
- Advanced NSCLC patients who cannot receive ALK inhibitors due to financial reasons;

1. Patients should provide tissue biopsy samples or archived tumor tissue samples if possible before enrollment;
2. Absence of brain metastasis, or asymptomatic brain metastasis, or symptomatic brain metastasis that has remained stable for more than 4 weeks after treatment;
3. Organ functions should meet the following requirements (blood components, cell growth factors, drugs that stimulate the growth of WBC or platelets, or drugs used to correct anemia are not permitted within 14 days prior to the laboratory test): ANC ≥ 1.5 × 10^9^/L; PLT ≥ 100 × 10^9^/L; Hb ≥ 90 g/L; TBIL ≤ 1.5 × ULN (TBIL ≤ 3.0 × ULN and DBIL ≤ 1.5 × ULN if Gilbert's syndrome); ALT and AST ≤ 2.5 × ULN; ALT and AST ≤ 5 × ULN if liver metastasis; Cr ≤ 1.5 × ULN; LVEF ≥ 50%;
4. Any surgery and prior radiotherapy (except for palliative radiotherapy)/procedures must have been completed at least 4 weeks prior to starting the treatment with the investigational product. Palliative radiotherapy must have been completed 48 hours prior to the start of treatment;
5. Any toxicity associated with previous anti-cancer treatment must have recovered to Grade ≤ 1 (except for alopecia);
6. The subject understands and voluntarily provides informed consent.

## Exclusion Criteria

1. Patients with leptomeningeal metastases;
2. Any clinically significant cardiovascular disease within 3 months prior to the first dose of the investigational product, including but not limited to: myocardial infarction, severe/unstable angina, coronary artery/peripheral artery bypass graft, congestive heart failure, cerebrovascular accident (including transient ischemic attack);
3. Patients with NCI-CTCAE Grade ≥ 2 arrhythmia, any grade of uncontrolled atrial fibrillation, or two continuously corrected QT interval (QTc) > 480 ms on ECG during screening;
4. Concomitant use of medications that may cause QTc prolongation or induce torsades de pointes within 14 days prior to the first dose of the investigational product or during treatment;
5. Grade ≥ 3 peripheral neuropathy (CTCAE V4.03);
6. Continuous use of corticosteroids for more than 30 days, or the need for chronic use of corticosteroids or other immunosuppressants;
7. A large area of diffuse/interstitial pulmonary fibrosis, or a known history of Grade 3 or 4 interstitial pulmonary fibrosis disease, which includes a history of pneumonitis, hypersensitivity pneumonitis, interstitial pneumonitis, interstitial lung disease, obliterative bronchiolitis, and pulmonary fibrosis, but does not include a history of radiation pneumonitis;
8. Patients with Grade > 1 nausea, vomiting, or diarrhea (CTCAE V4.03), other GI dysfunction or GI disease that may potentially affect drug absorption (such as ulcerative disease or malabsorption syndrome);
9. Patients currently on warfarin sodium (Coumadin) or other coumadin derivative anticoagulant treatment, or patients with a bleeding tendency or coagulation disorders;
10. Subject received other clinical trial treatment within 1 month prior to the first dose of the investigational product (if the medication received is a marketed drug, then refer to exclusion criteria #12);
11. Acute or chronic infectious disease, active hepatitis (hepatitis A, hepatitis B, or hepatitis C), or known HIV infection;
12. No more than 2 weeks between the most recent use of another anti-cancer treatment (half-life ≤ 3 days) and the first dose of the investigational product, or the most recent use of another anti-cancer treatment (half-life > 3 days) is less than 4 weeks. Patients may begin WX-0593 treatment 1 week after the last dose of crizotinib.
13. Patients who cannot suspend the use of a strong CYP3A4 inducer or inhibitor at least one week prior to this study and during the study. These drugs include but are not limited to carbamazepine, phenobarbital, phenytoin, rifabutin, rifampicin, rifapentine, tipranavir, ritonavir, St. John's wart, and ketoconazole;
14. Patients who cannot suspend the use of a CYP3A4 substrate at least one week prior to this study and during the study, with low therapeutic index;
15. Pregnant or lactating female patients or a positive pregnancy test at baseline for females of childbearing potential;
16. Female patients of childbearing age who are unwilling to use effective contraceptive measures or male patients who plan to have children during the study and within 6 months after the end of the study;
17. Concurrent diseases that may seriously affect patient safety or impact patient completion of the study as determined by the investigator (such as clinically uncontrolled hypertension (blood pressure > 160/110 mmHg), severe diabetes, thyroid disease, etc.);
18. Drug or alcohol abuse; Alcoholism refers to consumption of 4 or more alcoholic drinks during 2 hours. One alcoholic drink refers to 150 mL of wine, 350 mL of beer, or 50 mL of 80-proof distilled spirits (or equivalent alcohol concentration).
19. History of definitive neurological or mental disorder, including epilepsy and dementia;
20. Patients with other malignant tumors within 5 years prior to screening (except for cured basal cell carcinoma of the skin, cervical carcinoma *in situ*, and papillary thyroid carcinoma);
21. Patients with added risks associated with the study, or those who may interfere with the interpretation of study results as determined by the investigator, or those deemed unsuitable for this study by the investigator and/or the sponsor.

## Criteria for Discontinuation

1. Intolerable toxic reactions occur.
2. Poor subject compliance. The study is not carried out according to protocol requirements.
3. Voluntary withdrawal by the patient.
4. Other reasons requiring study discontinuation as determined by the investigator.

# TREATMENT REGIMEN

## Dose Regimen

### Dose escalation phase

- After a single dose on Day 1, the subject will be observed for 4 days and pharmacokinetic blood sampling will be simultaneously carried out. Repeated dosing will start on Day 5 and dosing will be carried out orally on an empty stomach every morning. One cycle consists of 21 days of repeated dosing.
- The dose escalation study for the next dose arm can begin after the last subject in the same dose arm completed the first cycle of treatment and no DLT is observed. Subjects in this dose arm will continue dosing with the original dose.
- The first tumor assessment is performed after 3 weeks of repeated dosing, then once every 6 weeks subsequently. Treatment is continued for CR/PR or SD and is terminated for PD.

### Population expansion phase

- Prior to using the dose of 120 mg QD or 180 mg QD, the subject will start at a dose of 60 mg QD for 7 consecutive days. If the subject is tolerated, then increase the dose to 120 mg or 180 mg QD. The drug should be administered orally once daily on an empty stomach for 21 days continuously for each cycle.
- The efficacy assessment is performed once every 6 weeks for the first 48 weeks. After 48 weeks, the frequency of tumor assessments is adjusted to once every 12 weeks. Dose administration will be continued if subject achieves CR/PR or SD and terminated if PD is observed. A confirmation assessment is performed 4 weeks after the first documented CR/PR. If the confirmation assessment is < 4 weeks from the next scheduled tumor assessment, then the next assessment is skipped.

## Investigational Product Packaging, Specification, and Storage

Main ingredient: WX-0593

Dosage form: tablets

Strength: 30 mg; 60 mg (based on WX-0593)

Manufacturer: Qilu Pharmaceutical Co., Ltd.

Storage: Tightly sealed.

## Management of the Investigational Product

The investigator is responsible for recording the receipt, dispensing, return, storage, and use of the investigational product in the related documents.

1. After the investigational product WX-0593 is delivered to the trial sites by Qilu Pharmaceutical Co., Ltd, the designated personnel at the trial site will receive the drugs and fill out the drug receiving form, which is retained at the trial site.
2. All investigational products sent to the trial site are limited to the clinical trial of the sponsor only and are not permitted to be used for other purposes.
3. The trial site must set an Investigational Product Usage Form to document the actual quantity of the investigational product dispensed to and returned from each subject. These records should be kept by the investigator.
4. During each onsite monitoring visit, the monitor will verify the actual quantity of the investigational product used by each patient.
5. All unused or partially used investigational products as well the all drug packaging will be collected by monitors of Qilu at the end of the trial.

# CONCOMITANT TREATMENTS AND DOSE ADJUSTMENTS

During the study, all antineoplastic drugs should be discontinued. During the DLT observation period, other drugs are not used in principle. If past treatment is continued during the trial, the dose used should be maintained at the same level as before the trial.

## Drugs Prohibited During the Study

Pre-clinical studies have shown that CYP3A4 is the major metabolic enzyme for WX-0593. Therefore, CYP3A4 inducers (such as dexamethasone, carbatazidine, rifampicin, phenobarbital, and phenytoin) and inhibitors (ketoconazole, itraconazole, erythromycin, clarithromycin, atazanavir, indinavir, nefazodone, nelfinavir, ritonavir, saquinavir, telithromycin, and voriconazole) are not permitted during the treatment period. CYP3A4 substrate (simvastatin, cyclosporine, and pimozide) and other drugs that are metabolized by CYP3A4 (such as benzodiazepines, dihydropyridines, calcium antagonists, and HMG-COA reductase inhibitors) should be used with caution.

## Drugs That Can Be Used with Discretion During the Study

Subjects should be closely observed if they develop adverse reactions and aggressive symptomatic treatment should be given if necessary. The drugs used should be documented and described in the CRF. The regulations used for the population expansion phase are the same as those for the continuous treatment period.

### Within the DLT observation period

If non-DLT adverse reactions occur during the first treatment cycle, treatment is not carried out in principle in order to observe possible adverse reactions towards the investigational product and their severity and reversibility. However, aggressive treatment must be carried out when DLT specified in the protocol occurs, and the drugs used must be documented in the CRF. Subjects who developed DLTs are withdrawn from the DLT observation period according to protocol stipulations. The investigator should decide whether to reduce the dose and continue the treatment if the subject benefits from the treatment.

### Outside the DLT observation period

When Grade < 3 toxicities occur at Cycle 2 and later at the dose escalation phase and the population expansion phase, the drug discontinuation or reduction should not be carried out in principle; treatment can be provided accordingly. When Grade ≥ 3 toxicities occur (Grade 2 and above cardiac dysfunction, renal impairment, etc.), treatment should be discontinued. After corresponding treatment has been given and toxicity has resolved to Grade ≤ 1 within 14 days (except for hyperlipidemia and transient liver enzyme abnormalities), the investigator can determine whether to resume the original dose or reduce to the next dose level (from 180 mg to 120 mg, from 120 mg to 90 mg). If Grade III or higher toxicity reoccurs after administration on recovery, the trial should be terminated and the patient should withdraw from the study. If treatment suspension exceeds 14 consecutive days or more than 14 days within one treatment cycle, the patient should withdraw from the study.

The rules for suspending and resuming treatment are outlined above. In special cases, the investigator can make decisions by considering risks and benefits based on the subject's specific conditions.

Hematology support: when a hematological toxicity occurs, the investigator may provide symptomatic treatment based on clinical manifestations. Treatments such as G-CSF and blood transfusions may be given when Grade 3 or higher hematological toxicities occur.

Diarrhea treatment: When diarrhea occurs, anti-diarrheal drugs are recommended along with appropriate symptomatic treatment.

Antiemetic treatment: Instead of being used prophylactically, antiemetics should be given when patients experience nausea and vomiting.

Hepatoprotection: When the patient experiences Grade 1 or higher hepatic dysfunction, hepatoprotection may be provided based on the investigator's judgment.

Other treatment: When the patient experiences Grade 2 or higher non-hematological toxicities, corresponding treatment may be provided based on the investigator's judgment.

Drugs used in the above treatments should be documented in detail in the CRF.

# SCHEDULE OF TRAIL

## Screening Period (within 28 Days Prior to the First Dose)

Subjects are selected for enrollment, signs the informed consent form, and undergoes screening procedures:

- CT/MRI scan: CT/MRI scan of chest, abdomen (entire abdomen and pelvis), and brain. These examinations need not be repeated if they are performed within 28 days prior to the first dose of the drug unless the investigator believes that a change in tumor burden has occurred in the patient.
- ALK/ROS1 status determination
- Demographic data collection: patient's identification card number, gender, age, address, and telephone number;
- Medical history, treatment history, ECOG, height, and weight;
- Vital signs (respiratory rate, body temperature, pulse, and, blood pressure); within 1 week prior to the first dose;
- Physical examination: within 1 week prior to the first dose;
- Laboratory examination: within 1 week prior to the first dose;

Laboratory examinations during the screening period include: routine blood test, coagulation function, routine urinalysis, routine stool test, blood biochemistry [hepatic function (ALT, AST, TP, ALB, TBIL, DBIL, TBA, Cr, BUN, UA, CK, CK-MB, and LDH), fasting blood glucose (GLU), blood lipids (TG, CHOL, LDL, and HDL), and electrolytes (K, Na, Cl, and Ca)], and pre-transfusion blood screening: hepatitis B surface antigen (HBsAg), hepatitis C antibody (HCV), human immunodeficiency virus antigen (HIV), and *Treponema pallidum* antibody;

- Pregnancy test: within 1 week prior to the first dose;
- ECG: within 1 week prior to the first dose;
- Echocardiogram: within 1 week prior to the first dose;
- Concomitant medications.

## Treatment period (after single dosing and Cycle 1 of repeated dosing, applicable for the dose escalation phase; population expansion phase begins with repeated dosing)

To avoid ambiguity, single dose and repeated dose time points are described as follows:

- Single dose: Day 1 of Week 1, recorded as Week 1 D1 of the single dosing;
- Cycle 1 of repeated dosing could start on D5+5 after single dosing, Weeks 2–4 (counted from the start of single dosing) or expressed as Weeks 1–3 of repeated dosing (counted from the first dose of repeated dosing), or D1–D21 of repeated dosing;

### Method of administration

- For Day 1 of single-dose administration in the dose escalation phase and Day 21 of the 1st cycle for continuous administration: subjects start to fast after 21:00 on the night before administration but are allowed to drink water. After fasting for at least 10 hours overnight, subjects take WX-0593 tablets along with 240 ml of lukewarm water at 8:00 in the morning on the day of administration. Other than before administration and within 1 hour after administration, subjects are allowed to drink water when necessary. 4 hours after administration, subjects consume low-fat light food (500-600 Kcal).
- Dose administration at other time points Subjects must take WX-0593 orally with lukewarm water one hour before meals.
- Alcohol and tobacco, coffee, carbonated drinks, dragon fruit, grapefruit, and mango, as well as fruit juices containing the above fruits (which may affect hepatic enzymes) are prohibited during the trial. Prolonged bed rest and strenuous exercise should also be avoided.

### Items should be carried out during single dosing and first cycle of repeated dosing (corresponding tests are only carried out on D7 and D21 of repeated dosing in the population expansion phase):

- Vital signs (respiratory rate, body temperature, pulse, and, blood pressure): before single dose administration, D4 ± 2 after single dose administration; and D7 ± 2, D14 ± 2, and D21 ± 2 after repeated dosing. The number of measurements must be increased if abnormalities occur during monitoring. During single dosing, electrocardiography monitoring should be carried out for generally 2 h. If electrocardiogram abnormalities occur, monitoring should be continued until 1 h after electrocardiogram returns to normal.
- Dose administration and drug administration record filling out
- Pharmacokinetic samples are collected at stipulated time points
- Physical examination: D4 ± 2 after single dosing; D7 ± 2, D14 ± 2, and D21 ± 2 after repeated dosing.
- ECOG PS score: D4 ± 2 after single dosing and D21 ± 2 after repeated dosing.
- Laboratory examination:

Blood routine test: D4 ± 2 after single dosing; D7 ± 2, D14 ± 2, and D21 ± 2 after repeated dosing.

Urinalysis: D4 ± 2 after single dosing; D7 ± 2, D14 ± 2, and D21 ± 2 after repeated dosing.

Stool routine test: D4 ± 2 after single dosing; D7 ± 2 and D21 ± 2 after repeated dosing.

Blood biochemistry: hepatic and renal functions (ALT, AST, TP, ALB, TBIL, DBIL, TBA, Cr, BUN, UA, CK, CK-MB, LDH), fasting blood glucose (GLU), blood lipids (TG, CHOL, LDL, HDL), electrolytes (K, Na, Cl, Ca) on D4 ± 2 after single dosing; D7 ± 2, D14 ± 2, and D21 ± 2 after repeated dosing;

- ECG: 4 h ± 30 min, 24 h ± 30 min, and D4 ± 2 after single dosing; D7 ± 2, D14 ± 2, and D21 ± 2 after repeated dosing.
- Echocardiogram: D21 ± 3 after continuous administration.
- CT/MRI scan: D21 ± 3 after repeated dosing (only for the dose escalation phase); after 60 mg QD is given for 7 consecutive days, the investigator may decide to perform a chest CT to monitor safety if clinically indicated (population expansion phase).

Monitor and document all adverse events. Document concomitant medications/treatments.

## Continuous treatment period (Week 4/5-)

The first efficacy assessment is carried out after 3 weeks of repeated dosing at the dose escalation phase and subsequent efficacy assessments will be carried out once every 6 weeks. In the population expansion phase, efficacy assessment is performed once every 6 weeks for the first 48 weeks and subsequent efficacy assessments will be carried out once every 12 weeks. Dose administration will be continued if subject achieves CR/PR or SD and will be terminated if PD is observed. In the population expansion phase, a confirmation assessment is performed 4 weeks after the first documented CR/PR. If the confirmation assessment is < 4 weeks from the next scheduled tumor assessment, then the next assessment is skipped.

At dose escalation phase, during continuous treatment, if PD occurs for a patient in a given dose arm and the investigator determined that the patient may benefit from an increased dose, then treatment could be continued with one dose increase with the consent of the patient. If the patient benefits from treatment but experiences an unacceptable toxicity, the investigator may decide to continue treatment with one dose reduction (refer to concomitant treatments and dose adjustments for details).

At the population expansion phase, one dose reduction is permitted if the subjects experience unacceptable toxicities at a certain dose during the treatment period and the continuous treatment period, provided that the treatment is effective (refer to concomitant treatments and dose adjustments for details).

### The following tests will be performed once every cycle (D21 ± 2), every 6 weeks (D42 ± 3) after Week 51 at the dose escalation phase; once every 6 weeks (D42 ± 3) in the first 48 weeks and every 12 weeks (D84 ± 3) after Week 48 at the population expansion phase:

- Laboratory examinations: blood routine test, routine urinalysis, hepatic and renal functions (ALT, AST, TP, ALB, TBIL, DBIL, TBA, Cr, BUN, UA, CK, CK-MB, LDH), fasting blood glucose (GLU), blood lipids (TG, CHOL, LDL, HDL), electrolytes (K, Na, Cl, Ca):
  - Physical examination;
  - ECOG PS score;
  - Vital signs;
  - ECG

### The following tests will be performed once every 6 weeks (D42 ± 7) at the dose escalation phase; once every 6 weeks (D42 ± 7) in the first 48 weeks and every 12 weeks (D84 ± 7) after Week 48 at the population expansion phase:

- - CT/MRI examinations. Subsequent imaging method should be the same as baseline, but only lesions identified at baseline are assessed, unless there are signs of metastasis.

At the population expansion phase, subjects must repeat the efficacy assessment 4 weeks after the first documented CR/PR. If the confirmation assessment is < 4 weeks from the next scheduled tumor assessment, then the next assessment is skipped.

### The following examinations will be performed when deemed necessary by the investigator:

- Color doppler echocardiography

## Safety Follow-Up Period

The following examinations are performed within 28 days after treatment completion:

- Vital signs
- Physical examination
- Laboratory examinations: blood routine test, routine urinalysis, hepatic and renal functions (ALT, AST, TP, ALB, TBIL, DBIL, TBA, Cr, BUN, UA, CK, CK-MB, LDH), fasting blood glucose (GLU), blood lipids (TG, CHOL, LDL, HDL), electrolytes (K, Na, Cl, Ca):
- ECG

The following examinations will be performed when deemed necessary by the investigator:

- - Color doppler echocardiography

The safety follow-up is not performed if the patient starts a new anti-cancer therapy within 2 weeks after stopping the study treatment. If the patient starts a new anti-cancer therapy within 3–4 weeks after stopping the study treatment, the safety follow-up visit should be completed prior to the new treatment. The safety follow-up visit is not performed if the patient starts a new anti-cancer therapy without notifying the investigator.

## Telephone follow-up (population expansion phase)

If subjects discontinue study treatment due to PD, the investigator will contact the patient or family once every 12 weeks ± 7 days via telephone to conduct survival visits and collect subsequent anti-cancer treatment and survival information, until death, withdraws informed consent, is lost to follow-up or refuses phone visits, or study completion.

## End of study

For subjects who can benefit from the treatment as determined by the investigator, administration will continue until progressive disease, intolerability, active withdrawal by patient, death, or end of study (whichever comes first). If the subject did not achieve PD at the end of the study and may still benefit from the study as comprehensively determined by the investigator, the sponsor will continue to supply the investigational product for free but examinations will no longer be arranged and safety and efficacy data will not be collected.

- - - - Dose escalation phase

Statistical analysis and trial summary can be carried out for the entire trial after all PK blood sampling has been completed for the last subject. The entire dose escalation study is completed two years after the last subject is enrolled.

- - - - Population expansion phase

After the first efficacy assessment and efficacy confirmation is completed for the last subject, observation is continued until 12 weeks since enrollment. Then, statistical analysis and trial summary can be carried out. The entire population expansion study can be completed when survival follow-up is 2 years after the last subject is enrolled.

# SAFETY EVALUATION

The investigational product for this trial is WX-0593 tablets. All subjects who received at least one dose of the investigational product will be included in the safety assessment.

## Definitions

Adverse event (AE): Refers to any untoward medical event during the study period (from signing of the informed consent form to 28 days after the last dose or start of other anti-cancer treatment; whichever comes first) and includes any untoward or unexpected signs (including laboratory test abnormalities), symptoms, or transient diseases related to the study treatment, regardless of whether there is a causal relationship between that event and treatment.

Significant adverse event: Refers to an adverse event or other significant laboratory test abnormalities other than serious adverse event leading to targeted medical measures (such as drug discontinuation, dose reduction, or symptomatic treatment).

Serious adverse event (SAE): Any of the following event: those that result in death, those that are life-threatening, those that require inpatient treatment or prolongation of hospitalization, those that result in permanent or significant disability/impairment, those that result in congenital deformities/birth defects, and those that the investigator deems as SAEs.

Unidentified/unexpected adverse event: AE whose nature, severity, and outcome does not match the investigator's brochure.

Adverse drug reaction (ADR): A harmful and unexpected reaction that has a causal relationship with drug usage when the drug is used normally within the stipulated dose. In new drug clinical trials, when the therapeutic dose is not determined, all harmful and unexpected reactions that have a causal relationship with drug usage are also considered to be adverse reactions.

## Potential Adverse Drug Reactions

The following adverse reactions may be seen with this investigational product. Monitor them closely and prepare a treatment plan.

1) The wards are equipped with ventilators, oxygen, and rescue medicines, and are attended by trained doctors and nurses.

2) If serious adverse events such as anaphylaxis, shock, or respiratory and circulatory failure occur during the study, treatment should be carried out according to local standard practice.

3) Potential adverse drug reactions:

According to the adverse reactions observed in clinical trials involving brigatinib, the following adverse reactions may occur: nausea, vomiting, fatigue, cough, headache, ALT elevation; possible serious adverse events include pneumonitis, dyspnea, hypoxia, and pleural effusion.

4) Management of adverse reactions:

Nausea/vomiting: Nausea and vomiting may be treated symptomatically according to the degree of severity, such as metoclopramide, ondansetron, granisetron, or tropisetron.

Diarrhea: Severe diarrhea should be monitored closely. If the patient becomes dehydrated, fluids and electrolytes should be given immediately. Standard antidiarrheal treatments such as loperamide should be started as soon as possible.

QT/QTc prolongation: Drug administration should be suspended in patients with significant QT/QTc prolongation (QT/QTc > 500 ms or > 60 ms from baseline with clinical significance) and symptomatic treatment should be provided. Drugs such as propranolol, phenytoin, carbamazepine, and guanethidine should be given immediately.

## Criteria for AE Severity

Grading criteria: CTC AE 4.03 (Appendix I).

The severity of the AE is a qualitative assessment of the range or intensity of the AE confirmed by the investigator or reported by the subject. The severity does not reflect the seriousness of the event and its correlation with the investigational product and only describes the severity or range of the AE experienced by the subject.

Grade 1: mild; asymptomatic or mild symptoms; clinical or diagnostic observations only; treatment not indicated

Grade 2: moderate; minimal, local or noninvasive intervention indicated; limiting age-appropriate instrumental activities of daily living.

Grade 3: severe or medically significant, but not immediately life-threatening; hospitalization or prolongation of hospitalization indicated; disabling; limiting self-care activities of daily living.

Grade 4: Life-threatening; urgent intervention indicated.

Grade 5: death due to adverse events

## Criteria for Causality Assessment Between AEs and Investigational Product

The National Center for ADR Monitoring recommends that the relationship between the drug and adverse event be determined as: definitely related, possibly related, probably related, unlikely related, not related, and not assessable The specific assessment method is as follows:

**Criteria for causality assessment between AEs and investigational product**

|  | a | b | c | d | e |
| --- | --- | --- | --- | --- | --- |
| Definitely related+ | + | - | + | + | Definitely related+ |
| Probably+ | + | - | + | ? | Probably+ |
| Possibly+ | + | ± | ± | ? | Possibly+ |
| Probably unrelated+ | - | ± | ± | ? | Probably unrelated+ |
| Unlikely- | - | + | - | - | Unlikely- |
| To be assessed | Additional materials for assessment are required | | | | |
| Not assessable | Data necessary for evaluation cannot be obtained | | | | |

Note: "+": definitely; "-": unlikely; "±": difficult to confirm or reject; "?": unclear

a Whether there is a reasonable sequence between the time of initial dosing and the time of adverse reaction occurrence;

b Whether the suspected adverse reaction conforms to the type of adverse reaction known for the drug;

c Whether the suspected adverse reaction can be explained by concomitant medications, clinical condition of patient, or other treatments;

d Whether the reaction is alleviated or disappeared after drug discontinuation or dose reduction;

e Whether the same reaction occurs again after re-exposure to the suspected drug;

## AE Reporting and Management

All AE information, regardless of whether it is narrated by the subject or obtained through questioning by the investigator, or obtained through physical examinations, laboratory tests, or other methods must be recorded and described in the AE page of the CRF, regardless of whether the investigator considers it to be related to the investigational product. Details of AE signs and symptoms are included: start date and time, date and time of resolution, event description, severity, relationship with the investigational product, measures adopted, and outcome.

The investigator must also submit clinically significant or significantly abnormal laboratory test results to the sponsor and record in the AE page of the CRF. All AEs must be reported to the sponsor regardless of the relationship thereof with the selected investigational product. Any abnormal laboratory test result that requires hospitalization or prolongs hospitalization must also be reported if the results are related to drug overdose or considered to be clinically significant. Once these abnormal laboratory test results are considered to be SAEs by the investigator, they must be promptly reported to drug regulatory departments and medical management must be carried out.

AEs must be followed up until the events disappeared or are stable. If the subject does not return for follow-up and AE outcome cannot be obtained, the outcome of that AE will be handled as lost to follow-up.

Medical conditions or diseases that are present before the study are considered AEs only if they worsen after the study has started.

## Serious Adverse Event Reporting and Management

Any SAE that occurs during the clinical study must be immediately reported to the principal investigator, Ethics Committee of the study site, Food and Drug Administration of the area (province or city) in which the investigator is based at and the sponsor, regardless of whether it is related to the investigational product; and the Office of Drug Research and Supervision, Department of Registration, National Medical Products Administration and health administrative departments must be notified within 24 hours. During the trial, the adverse event form must be completed by the investigator. The time of occurrence, severity, duration, measures adopted, and outcomes should be documented.

The serious adverse event reporting route is as follows:

| Institution | Contact information |
| --- | --- |
| Qilu Pharmaceutical Co., Ltd. | E-mail: qlsaemailbox@qilu-pharma.com  Telephone: 0531-83126996  Fax: 0531-83126688 (ext. 6996) |
| Department of Registration and Division of Drug Research Supervision, National Medical Products Administration | Telephone: 010-68313344  Fax: 010-88363228 |
| Department of Medical Safety and Hematology, Bureau of Medical Administration, National Health and Family Planning Commission | Telephone: 010-68792201  Fax: 010-68792734 |

SAEs that are unresolved at the end of the trial or at the time of premature withdrawal by the subject must be followed up until one of the following situations occur:

1) The SAE is resolved

2) The SAE has stabilized

3) The SAE has recovered to baseline levels (if baseline values are available)

The SAE can be attributed to drugs other than the investigational product or factors that are not related to the study, or when more information cannot be obtained (the patient or medical worker refuses to provide more information or there is evidence showing that the patient is still lost to follow-up after maximum efforts).

# PHARMACOKINETIC STUDY

Single dose and repeated dose pharmacokinetic studies will be simultaneously carried out during the dose escalation phase. PK blood sampling and tolerability observation will be carried out after administrations of a single dose. Continuous dosing will start on Day 5 after a single dose was administered. PK blood sampling will be carried out on Day 21 of continuous dosing. After the end of the tolerability trial for each arm, since there were good safety and benefits to patients, the investigator selected 3–6 dosage arms and additional patients were enrolled into these dose arms for single-dose and repeated-dose pharmacokinetic studies. At least 8 subjects were included in each arm. The PK blood sampling time points in the entire trial will be adjusted according to the preliminary results of the first dose arm.

With regards to patients with brain metastases, a few of them can be selected after integrated judgment by the investigator and after consent was obtained from the patients. Cerebrospinal fluid will be collected at the first imaging assessment at the end of 6 continuous weeks of treatment in the 2nd cycle or 3 h (+ 0.5 h) after dosing in other stable states. Drug concentrations in the cerebrospinal fluid will be compared with plasma drug concentrations (blood samples should be simultaneously collected) to determine the relationship between efficacy and concentration of WX-0593 in brain metastasis patients.

## Sample Collection and Storage

Plasma sample collection: Blood is collected from the median cubital vein. 3 mL of blood is collected using an anticoagulant tube each time and immediately placed in a wet ice bath. Then the sample is centrifuged at 4 °C, 3000 rpm for 10 minutes. The supernatant is separated and stored in 3 EP tubes at -20 °C or below.

Cerebrospinal fluid collection: Cerebrospinal fluid is collected according to the standard medical procedures of the hospital. 1–2 ml of cerebrospinal fluid is collected into a sterile test tube each time and immediately placed in wet ice bath before storage at -20 °C or below;

## Sampling Time Point

- Blood sampling time points for single dose: Blood samples are collected before dosing and 0.5 h, 1 h, 2 h, 3 h, 4 h, 5 h, 6 h, 8 h, 12 h, 24 h, 32 h, 48 h, and 72 h after dosing (after adjustment according to the pharmacokinetic parameters of patients in the first dose arm).
- Blood sampling time points for repeated dosing: Repeated dosing starts 4 days after single dose administration. Blood samples are collected before repeated dosing on Days 7, 14, and 21 before repeated dosing, and within 24h after dosing (0.5 h, 1 h, 2 h, 3 h, 4 h, 5 h, 6 h, 8 h, 12 h, 24 h) for measurement of blood pharmacokinetic parameters.
- Cerebrospinal fluid samples are collected at the first imaging assessment at the end of 6 continuous weeks of treatment in the 2nd cycle or 3 h (+0.5 h) after dosing in other stable states. At the same time, venous blood is collected 3h (+0.5 h) after dosing.

## Test method

The plasma concentration of WX-0593 will be determined using LC-MS.

- Specificity

Endogenous substances as well as metabolites and degradation products in plasma should not interfere with the testing of the sample. Chromatogram of blank plasma is provided: chromatogram of blank plasma plus control substance as well as chromatogram of plasma after administration;

- Standard Curve and Quantitative Range

The linear equations and correlation coefficients of the standard curve are provided to illustrate the degree of linearity. The quantitative range should cover the range of all plasma sample concentrations to be tested. The variation of each concentration on the standard curve is within the acceptable range (variation is within ±20% for low concentrations, and ±15% for the remaining concentrations). The concentration range is wider, and the standard curve is calculated using curve weighting.

- Lower limit of quantitation

The lower limit of quantitation (LLOQ) should at least be the drug concentration at 3–5 half-lives of the sample or 1/10–1/20 of the C_max_. The accuracy should be within the range of 80-120% of the actual concentration, and the relative standard deviation should be less than 20%. At least 5 LLOQ samples should be tested to evaluate the accuracy and precision.

- Precision and accuracy

Precision and accuracy should be evaluated using low, medium, and high concentrations. The intra-batch and inter-batch RSD of the quality control samples may be used to evaluate the precision of the method. Generally, RSD should be less than 15%, and less than 20% near the LLOQ. Accuracy, or relative percent recovery, is required to be between 85–115%, and between 80–120% near the LLOQ.

- Sample stability

The storage conditions and time of plasma samples should be determined, including: long-term stability, freeze-thaw stability, bench-top stability, and processed sample stability.

- Extraction efficiency

The extraction efficiency of a low, medium, and high concentration is evaluated. Results should be precise and reproducible.

- Matrix effect evaluation

Matrix effect: Matrix effect is the effect of endogenous substances in a biological sample on the mass spectral response of the analyte, usually expressed as M = A/B. A: A blank biological matrix is processed according to the sample processing method except for adding the analyte and the internal standard. Then a certain concentration of the standard solution is added to the extracted residue, vortexed, loaded, then a peak area is obtained. B: Directly loading a solution with the same concentration, and then a peak area is obtained. Calculating A/B to obtain the matrix effect, M. The experiment should be conducted with 3 concentrations, high, medium, and low. If the linear range is wide, 4 concentrations can be used to evaluate the matrix effect. At least 4 replicates are prepared for each concentration, and each sample is loaded once. Determining the M value for each concentration. The results should be consistent, precise, and reproducible. To ensure the sensitivity of the test, it is generally required that the mean M value obtained at each concentration should be 80–120%, and RSD should be less than 15%.

## PK Parameters Analysis

- Single dose: time to peak (T_max_), maximum blood drug concentration (C_max_), drug concentration-time curve (AUC), elimination half life (t_1/2_). Repeated-dose: trough concentration at steady state (C_ssmin_), peak concentration at steady state (C_ssmax_), mean concentration at steady state (C_ss-av_), elimination half life (t_1/2_), area under plasma concentration-time curve at steady state (AUC_ss_), DF fluctuation coefficient (DF), and clearance (CLs).

# TREATMENT RESPONSE EVALUATION

## 11.1 Baseline Assessment

Imaging assessment (CT or MRI) should be performed within 28 days prior to the first dose: CT/MRI scanning of the chest, abdomen, pelvis, and brain will be carried out. The RECIST 1.1 criteria will be employed to determine target lesions. The size of target lesions will be measured and recorded.

## 11.2 Efficacy Evaluation

The first efficacy assessment is carried out after 3 weeks of repeated dosing at the dose escalation phase and subsequent efficacy assessments will be carried out once every 6 weeks. At the population expansion phase, efficacy assessment is performed once every 6 weeks for the first 48 weeks and subsequent efficacy assessments will be carried out once every 12 weeks and continued until PD is observed in radiologic examinations. At the population expansion phase, a confirmation assessment is performed 4 weeks after the first documented CR/PR. If the confirmation assessment is < 4 weeks from the next scheduled tumor assessment, then the next assessment is skipped. CT or MRI scanning must be performed as soon as there is suspicion of the progressive disease (PD). Except for PD, subjects who complete treatment will proceed with imaging assessments as planned until progressive disease, start of a new anti-cancer therapy, withdrawal of consent, loss to follow-up or death (whichever occurs first).

Efficacy assessment is based on RECIST l.1 criteria (see Appendix III for details).

Primary endpoint: objective response rate (ORR): number of subjects with (CR + PR)/total number of subjects × 100%

Secondary endpoints: progression-free survival (PFS), disease control rate (DCR), time to progression (TTP), duration of response (DOR) and CNS ORR.

- Objective response rate (ORR) refers to the proportion of CR+PR patients whose tumor shrinks to a certain size and is maintained for a certain period of time.
- Complete response (CR): Complete disappearance of the target lesion, any pathological lymph nodes (whether target or non-target) must have reduction in short axis to <10 mm.
- Partial response (PR): At least a 30% decrease relative to baseline in the sum of diameters of target lesions.
- Disease control rate (DCR): refers to the proportion of CR+PR+SD patients whose tumor (solid tumors) shrinks to a certain size and is maintained for a certain period of time.
- Stable disease (SD): Neither sufficient shrinkage to qualify for PR nor sufficient increase to qualify for PD, taking as reference the smallest sum diameters while on study.
- Progressive disease (PD): At least a 20% increase in the sum of diameters of target lesions, taking as reference the smallest sum on study (this includes the baseline sum if that is the smallest on study). In addition, the sum must also demonstrate an absolute increase of at least 5 mm (the appearance of one or more new lesions is also considered progression).
- Duration of response (DOR): refers to the time from the first CR or PR is met to the first documentation of PD or death from any cause.
- Progression-free survival (PFS) is the time from enrollment to PD or death.
- Time to progression (TTP): refers to the time from enrollment to objective tumor progression.

# STUDY MANAGEMENT

## Ethics and Informed Consent

Compliance with documents

The trial must be conducted in strict accordance with the trial protocol and relevant SOPs. The design and implementation of the study protocol must be in accordance with the following documents:

1. "Declaration of Helsinki"
2. "Drug Administration Law of the People's Republic of China"
3. Administrative Measures for Drug Registration
4. Good Clinical Practice

Ethical standards

The clinical trial must comply with the "Declaration of Helsinki" (2008 edition), "Good Clinical Practice (GCP)" issued by NMPA, and other relevant regulations. Approval from the ethics committee of the leading site must be obtained before initiating this study. During the study, any revisions to the study protocol must be reported to the ethics committee. The investigator is responsible for submitting an annual report of the trial regularly as required by the ethics committee, and notifying the ethics committee when the study has been completed.

Informed consent

During the informed consent process, the investigator must explain to every subject the nature and purpose of the trial, study procedures, anticipated duration, potential risks and benefits, as well as any potential discomforts. Each subject must understand that participation in this trial is voluntary. The subject may withdraw from the trial and withdraw informed consent at any time, and doing so will not affect subsequent treatments or the relationship with the doctor.

Informed consent should be given in a standard written format and jargon should be avoided. Each informed consent form must include all of the above information, and include a voluntary statement. The informed consent form should be submitted to the ethics committee for review and approval.

After explaining the basic information regarding the trial and confirming that each participant understands the purpose of the trial, the investigator should ask each subject to sign the name and date on the informed consent form. The subject should read and consider the statements in the informed consent form before signing and dating the document, and should obtain the original copy of the signed document. Subjects may not be enrolled in the trial without first be provided an informed consent.

## Investigational Product Management

Designated personnel is responsible for the management, dispensing, and return of the investigational product used in this clinical trial. The investigator must ensure that all investigational products are used only by subjects participating in this clinical trial. The dosage and administration must comply with the trial protocol, remaining investigational products will be returned to the sponsor and must not be given to anyone who does not participate in this clinical trial.

The monitor is responsible for monitoring the supply, usage, and storage of the investigational product, and the handling of remaining drugs.

## Protocol Revisions

No one may make revisions to the protocol except for the sponsor. Any necessary changes to the protocol must be made in the form of protocol revisions, and must be reviewed or filed by the ethics committee after obtaining signatures from the sponsor and the principal investigator. Details of previous revisions should be explained in the protocol.

## Monitoring

The sponsor will appoint a person with appropriate medical, pharmacy, or relevant professional qualifications as the monitor of this clinical trial. The monitor must have received the necessary training and be familiar with the GCP and applicable regulations in order to monitor and report the progress of the trial and verify data. The monitor must ensure that subjects' rights and interests are protected during the trial, that trial records and reported data are accurate and complete, and that the trial is conducted in accordance with the approved protocol, GCP, and applicable regulations.

All adverse events and serious adverse events during the clinical trial must be monitored to ensure that all events are documented and reported accurately, reliably, and in a timely manner.

## Auditing

The sponsor may entrust a third-party or the sponsor's quality assurance department to audit the trial. Auditing includes: the supply of the drug, all required trial documents, documentation of the informed consent discussion, as well as consistency between case report forms and source documents. The content and scope of the audit can also be increased accordingly. The investigator must agree to participate in the auditing at a reasonable time and in a reasonable manner.

## Quality Control and Assurance

- The trial sites must be NMPA-approved drug clinical trial sites with clinical research conditions.
- Study staff must receive clinical trial training and work under the supervision of senior professionals;
- Before the trial, the clinical ward must be examined as meeting the standardized requirements, ensuring that the site is fully equipped with emergencies;
- Professionals should instruct subjects to take the medication and should fully understand the use of the drug to ensure subject compliance;
- Each trial site must conduct the trial in strict accordance with the study protocol and fill in the CRF truthfully;
- The monitor will monitor the clinical trial in accordance with SOP, ensuring that all data are documented and reported accurately and completely, that all CRF are filled out correctly and consistent with source data, and that the trial is implemented according to the clinical study protocol.
- In the event of an unexpected serious adverse reaction, the sponsor must immediately notify all trial sites and suspend the trial when necessary.
- Each participating trial site must accept auditing by the sponsor and regulatory authorities. It is particularly important that investigators and the relevant staff provide convenience and time for monitoring and auditing.

## Data Management

- An electronic data management system is used in this study. eCRF construction: The data manager must construct the electronic case report form (eCRF) according to the study protocol.
- User permission assignment: The data management personnel will create accounts and assign permission based on different user roles such as data entry personnel, investigator, and clinical monitor. The data entry personnel have access to data entry, modification, and queries. The investigator has access to modification, browsing, queries, and review. The monitor has access to browsing and sending queries. The data management personnel have access to browsing, sending queries, and locking data.
- Data entry: The clinical investigator or data entry personnel designated by the investigator (clinical coordinator) must enter data from the medical records into the eCRF accurately and in a timely manner.
- Queries sending and resolution: The monitor and data management personnel send queries through the eCRF. The data entry personnel and the investigator must respond to the queries and correct erroneous data. Queries may be resent when necessary. All records should be saved in the eCRF.
- Data modification and review: The data entry personnel or the investigator may make modifications after verifying data. The reasons for the modifications should be provided in the eCRF. The investigator has access to reviewing all final data.
- Data lock and export: After reviewing all data, the data management personnel will lock the data. If modifications are required after locking the data, signatures must be obtained from the sponsor, investigator, data entry personnel, monitor, and data management personnel. All data is ultimately imported into the designated database by the data management personnel and submitted to the statistician for analysis.

## Protocol Violations

Major protocol violations: Use of other anti-cancer treatment, enrollment of subjects who do not meet inclusion criteria, treatment suspension more than 14 days due to reasons other than toxicity.

All protocol requirements must be strictly implemented. Any intention or non-intentional deviations or violations of the study protocol and GCP are considered protocol deviations or protocol violations. If a protocol deviation is found during the monitoring process, the investigator or the monitor should fill out the protocol violation record, detailing the time that the violation was found, the time and process of the event, the reason and corresponding measures taken. The protocol violation record should be signed by the investigator and submitted to the ethics committee and the sponsor according to trial site requirements.

## Document Storage

In order to ensure the evaluation and supervision of the clinical study by the NMPA and the sponsor, the investigator should agree to retain all study-related documents, including the subjects' source documents during hospitalization, informed consent forms, case report forms, and detailed drug dispensing records. The study-related records should be retained by the trial site until 5 years after the end of the trial and retained by the sponsor until 5 years after product marketing. All information of this clinical study belongs to Qilu Pharmaceutical Co., Ltd. Except when required by the NMPA, the investigator must not provide the information to a third-party in any form without the written consent of the sponsor.

The trial site must retain source documents of each subject. All information in the case report form must be derived from these source documents. Source documents should contain all demographics information and treatment information, including laboratory test results and ECG, as well as the informed consent form signed by the subject.

The following basic documents must be retained by the trial site until the time required by regulations. Basic documents include:

1) NMPA Clinical Study Approval;

2) Investigational Product Certificate of Analysis;

3) IRB/EC approval of the trial protocol and all revisions;

4) All source documents and laboratory test records;

5) Case report form (CRF) and original medical records;

6) Informed consent form;

7) Laboratory data before and after the trial;

8) Vital signs measurements during the trial;

9) Other trial-related documents.

## Publication of Study Results

All articles and reports related to the trial should be published or made public with the written consent of the sponsor.

## Duties and Regulations

The sponsor will provide the investigator with materials such as the investigator's brochure and funding, and provide qualified investigational products (including the control drugs) and study materials (including the drug clinical study approval, the sponsor's business license, the drug manufacturing license, copy of the GMP certificate, and certificate of analysis) for free. The sponsor and the investigator will jointly develop the trial protocol and determine it together. The investigator will carry out the trial in accordance with the study protocol and relevant SOPs, as well as current regulations.

The investigator should keep all information provided by the sponsor strictly confidential, and likewise for other study staff and the ethics committee. The information provided to the investigator shall not be disclosed to others without the written consent of the sponsor.

All data and results of the trial are jointly owned by the investigator and the sponsor, and shall not be published by the investigator without the consent of the sponsor. All articles must be submitted to the sponsor prior to being published. The sponsor will review the accuracy of the paper, confirm that confidential information has not been disclosed, and add any relevant information.

# STATISTICAL ANALYSIS

## General Principles

All statistical analyses are performed using SAS 9.4 (or later).

Quantitative data will be summarized using N, mean, standard deviation, median, maximum, and minimum.

Qualitative data will be summarized using frequency and percentage.

Assuming a significance level of α = 0.05 (two-sided), there is a significant difference between treatment arms if *p* < 0.05.

## Management of missing values

Subjects with missing post-treatment imaging assessments will be reviewed one-by-one to determine the censor time when calculating the time variables involved (such as TTP).

Missing values are not estimated for baseline and safety data.

## Selection of Statistical Analysis Data

- Full Analysis Set

Efficacy will be analyzed on all enrolled subjects who received at least one dose of the investigational product based on the intention-to-treat principle. Missing data will not be imputed.

- Per-protocol analysis set

All cases meeting the trial protocol, with good compliance and without major protocol violations. Missing data will not be imputed.

- DLT Set

Patients who developed DLT during the first treatment cycle or received treatment for more than 14 days. This set is used to calculate the proportion of subjects with DLT.

- Safety Set

All enrolled subjects who received at least one dose of the investigational product and have post-treatment safety records are included in the Safety Set. This analysis set is used for the safety analysis.

Efficacy of the investigational product is analyzed using the PPS and FAS. Results are primarily based on the FAS.

- PK Analysis Set

Subjects with at least 1 measurable PK parameter, with no major protocol violations that significantly affect PK assessment.

## Statistical Analysis Plan

The results of this trial will be summarized mainly through descriptive statistics. Quantitative data will be summarized using mean, standard deviation, median, maximum, and minimum values. Qualitative data and rank data will be summarized using frequency (proportion), ratio, and confidence interval.

All statistical analyses will be computed using the SAS 9.4 software. All statistical tests will be two-sided tests where a *p*-value less than or equal to 0.05 is considered statistically significant, and 95% confidence level will be used for confidence interval.

⚫ Basic Characteristics: The mean, SD, median, maximum, and minimum of quantitative variables such as age, height, and weight will be computed. The frequency and percentage of qualitative variables such as gender and ECOG will be computed.

⚫ Tolerance evaluation: The number of cases, number of subjects, and incidence rate of all AEs, drug-related AEs, SAEs, drug-related SAEs, AEs resulting in drop-out, and deaths associated with AEs in this trial will be summarized mainly by employing descriptive statistical analysis. Normal laboratory test results before drug administration but abnormal test results after treatment as well as the relationship between abnormalities and the investigational product will be described. The mean, standard deviation, median, minimum, and maximum of vital signs (blood pressure, heart rate, temperature, and respiratory rate) and laboratory markers of different single dose arms before and after treatment will be calculated. The paired t-test is performed for before and after comparison when necessary. Changes in vital signs and laboratory markers in the different dose arms will be used for trend test. The various post-dosing time points of the repeated-dose arms will be compared with those before dosing.

Efficacy assessment:

The percent and 95% CI metastasis objective response rate (ORR), disease control rate (DCR), and CNS response rate will be computed.

Progression-free survival (PFS), duration of response (DOR), curves will be plotted by using Kaplan-Meier method, and the median PFS and DOR and their respective 95% CI will be computed.

Pharmacokinetic parameters: single dose: time of peak (T_max_), maximum serum concentration (C_max_), drug concentration-time curve (AUC_0-t_ and AUC_0-∞_), elimination half life (t_1/2_). Repeated-dose: trough concentration at steady state (C_ssmin_), peak concentration at steady state (C_ssmax_), mean concentration at steady state (C_ss-av_), elimination half life (t_1/2_), area under plasma concentration-time curve at steady state (AUC_ss_), fluctuation coefficient (DF), and systemic clearance (CLs). Descriptive statistical analysis will be carried out by dose arms. The statistics include number of subjects, mean, standard deviation, median, minimum, maximum, coefficient of variation, and 95% confidence interval.

# TRIAL PROGRESS

Anticipated duration: Sep. 2017 to Dec. 2020.

REFERENCES

[1] FDA: Ceritinib Clinical Review.

[2] Doebele, R. C., Pilling, A. B., Aisner, D. L., Kutateladze, T. G., Le, A. T., Weickhardt, A. J., ... & Varella-Garcia, M. (2012). Mechanisms of resistance to crizotinib in patients with ALK gene rearranged non–small cell lung cancer. *Clinical cancer research*, *18*(5), 1472-1482.

[3] Ou, S. H. I., Ahn, J. S., De Petris, L., Govindan, R., Yang, J. C. H., Hughes, B. G. M., ... & Mekhail, T. (2015, May). Efficacy and safety of the ALK inhibitor alectinib in ALK+ non-small-cell lung cancer (NSCLC) patients who have failed prior crizotinib: An open-label, single-arm, global phase 2 study (NP28673). In *ASCO Annual Meeting Proceedings* (Vol. 33, No. 15_suppl, p. 8008).

[4] Camidge, D. Ross, et al. "Activity and safety of crizotinib in patients with ALK-positive non-small-cell lung cancer: updated results from a phase 1 study." *The lancet oncology* 13.10 (2012): 1011-1019.

[5] Updated results of a global phase II study with crizotinib in advanced ALK-positive non-small cell lung cancer (NSCLC)[J]. *Ann. Oncol.,* 2012 (23): 402-402.

[6] Shaw, Alice T., et al. "Ceritinib in ALK-rearranged non–small-cell lung cancer." *New England Journal of Medicine* 370.13 (2014): 1189-1197.

[7] Katayama, Ryohei, et al. "Mechanisms of acquired crizotinib resistance in ALK-rearranged lung cancers." *Science translational medicine* 4.120 (2012): 120ra17-120ra17.

[8] Mologni, Luca. "Current and future treatment of anaplastic lymphoma kinase-rearranged cancer." *World Journal of Clinical Oncology* 6.5(2015):104-8.

[9] Gadgeel, Shirish M, et al. "Safety and activity of alectinib against systemic disease and brain metastases in patients with crizotinib-resistant ALK-rearranged non-small-cell lung cancer (AF-002JG): Results from the dose-finding portion of a phase 1/2 study." *Lancet Oncology* 15.10(2014):1119-1128.

[10] Ou SI, et al. "Alectinib in Crizotinib-Refractory ALK-Rearranged Non-Small-Cell Lung Cancer: A Phase II Global Study." *Journal of Clinical Oncology* (2015).

[11] Ou, Sai Hong Ignatius, et al. "Efficacy and safety of the ALK inhibitor alectinib in ALK+ non-small-cell lung cancer (NSCLC) patients who have failed prior crizotinib: An open-label, single-arm, global phase 2 study (NP28673).." *Journal of Clinical Oncology 33:5s, 2015 (suppl; abstr 8008)*(2015).

[12] Camidge, D. Ross, et al. "Safety and efficacy of brigatinib (BRIGATINIB) in advanced malignancies, including ALK+ non-small cell lung cancer (NSCLC)." *ASCO Annual Meeting Proceedings*. Vol. 33. No. 15_suppl. 2015.

[13] ICH S9: Guidance for Industry S9 Nonclinical Evaluation for Anticancer Pharmaceuticals.*http://www.ich.org/products/guidelines/safety/safety-single/article/nonclinical-evaluation-for-anticancer-pharmaceuticals.html*

[14] Kerstein, D., et al. "LBA4 * EVALUATION OF ANAPLASTIC LYMPHOMA KINASE (ALK) INHIBITOR BRIGATINIB [AP26113] IN PATIENTS (PTS) WITH ALK+ NON-SMALL CELL LUNG CANCER (NSCLC) AND BRAIN METASTASES." *Annals of Oncology* 26.suppl 1(2015):i60-i61.

[15] Rosell, R., et al. "99O * PHASE 1/2 STUDY OF AP26113 IN PATIENTS (PTS) WITH ADVANCED MALIGNANCIES, INCLUDING ANAPLASTIC LYMPHOMA KINASE (ALK)-POSITIVE NON-SMALL CELL LUNG CANCER (NSCLC): ANALYSIS OF SAFETY AND EFFICACY AT SELECTED PHASE 2 DOSES." *Annals of Oncology* 26.suppl 1(2015).

[16] Camidge, D. R., L. Bazhenova, and R. Salgia. "First-in-human dose-finding study of the ALK/EGFR inhibitor AP26113 in patients with advanced malignancies: Updated results." *Journal of Clinical Oncology* (2013).

Appendix I: NCI-Common Terminology Criteria 4.03 for Toxicity

| Grade | | | | | | | | | | | | | |
| --- | --- | --- | --- | --- | --- | --- | --- | --- | --- | --- | --- | --- | --- |
| Adverse events | **1** | | **2** | | | | **3** | **4** | | **5** | | | |
| **Blood and lymphatic system disorders** | | | | | | | | | | | | | |
| Anemia | Hemoglobin < LLN-10.0 g/dL; < LLN-6.2 mmol/L; < LLN-100 g/L | Hemoglobin < 10.0–8.0 g/dL; <6.2–4.9 mmol/L; < 100–80 g/L | | | Hemoglobin < 8.0–6.5 g/dL; < 4.9–4.0 mmol/L; < 80–65 g/L; require blood transfusion | | | Life-threatening consequences; urgent intervention indicated. | | | Death | | |
| Definition: A disorder characterized by a reduction in the amount of hemoglobin in 100 mL of blood. Signs and symptoms of anemia may include pallor of the skin and mucous membranes, shortness of breath, palpitations of the heart, soft systolic murmurs, lethargy, and fatigability. | | | | | | | | | | | | | |
| Bone marrow hypocellular | Mildly hypocellular or ≤ 25% reduction from normal cellularity for age | Moderately hypocellular or >25% - <50% reduction from normal cellularity for age | | | Severely hypocellular or >50 - ≤75% reduction cellularity from normal for age | | | Aplastic persistent for longer than 2 weeks | | | Death | | |
| Definition: A disorder characterized by the inability of the bone marrow to produce hematopoietic elements. | | | | | | | | | | | | | |
| Disseminated intravascular coagulation (DIC) | — | Laboratory findings with no bleeding | | | Laboratory findings with bleeding | | | Life-threatening consequences; urgent intervention indicated. | | | Death | | |
| Definition: A disorder characterized by systemic pathological activation of blood clotting mechanisms which results in clot formation throughout the body. There is an increase in the risk of hemorrhage as the body is depleted of platelets and coagulation factors. | | | | | | | | | | | | | |
| Febrile neutropenia | — | — | | | Present | | | Life-threatening consequences; urgent intervention indicated. | | | Death | | |
| Definition: Neutropenia with fever | | | | | | | | | | | | | |
| Definition: A disorder characterized by failure of the left ventricle to produce adequate output despite increases in diastolic pressure and end-diastolic volume. Clinical manifestations include signs such as dyspnea and orthopnea, as well as pulmonary congestion and pulmonary edema. | | | | | | | | | | | | | |
| **Gastrointestinal disorders** | | | | | | | | | | | | | |
| Diarrhea | Increase of <4 stools per day over baseline; mild increase in ostomy output compared to baseline | Increase of 4–6 stools per day over baseline; moderate increase in ostomy output compared to baseline | | | Increase of ≥ 7 stools per day over baseline; fecal incontinence; hospitalization indicated; severe increase in ostomy output compared to baseline; limiting self care activities of daily life | | | Life-threatening consequences; urgent intervention indicated. | | | Death | | |
| Definition: A disorder characterized by an increase in frequency and/or loose or watery bowel movements. | | | | | | | | | | | | | |
| Dry mouth | Symptomatic (e.g., dry or thick saliva) without significant dietary alteration; unstimulated saliva flow >0.2 ml/min | Moderate symptoms; oral intake alterations (e.g., copious water, other lubricants, diet limited to purees and/or soft, moist foods); unstimulated saliva 0.1 to 0.2 ml/min | | | | Inability to adequately aliment orally; tube feeding or TPN indicated; unstimulated saliva < 0.1 mL/min | | — | | | — | | |
| Definition: A disorder characterized by reduced salivary flow in the oral cavity. | | | | | | | | | | | | | |
| Definition: A disorder characterized by an abnormal communication between the duodenum and another organ or anatomic site. | | | | | | | | | | | | | |
| Nausea | Loss of appetite without alteration in eating habits | Oral intake decreased without significant weight loss, dehydration or malnutrition | | | Inadequate oral caloric or fluid intake; tube feeding, TPN, or hospitalization indicated | | | — | | | — | | |
| Definition: A disorder characterized by a queasy sensation and/or the urge to vomit. | | | | | | | | | | | | | |
| Obstruction gastric | Asymptomatic; clinical or diagnostic observations only; intervention not indicated | Symptomatic; altered GI function; limiting instrumental activities of daily life | | | Hospitalization indicated; invasive intervention indicated; limiting self care activities of daily life; disabled | | | Life-threatening consequences; urgent operative intervention indicated | | | Death | | |
| Definition: A disorder characterized by blockage of the normal flow of the contents in the stomach. | | | | | | | | | | | | | |
| Vomiting | 1–2 episodes (separated by 5 minutes) in 24 h | 3–5 episodes (separated by 5 minutes) in 24 h | | | ≥ 6 episodes (separated by 5 minutes) in 24 h; tube feeding, TPN, or hospitalization indicated | | | Life-threatening consequences; urgent intervention indicated. | | | Death | | |
| Definition: A disorder characterized by the reflexive act of ejecting the contents of the stomach through the mouth. | | | | | | | | | | | | | |
| Diarrhea | Increase of <4 stools per day over baseline; mild increase in ostomy output compared to baseline | | Increase of 4–6 stools per day over baseline; moderate increase in ostomy output compared to baseline | | | Increase of ≥ 7 stools per day over baseline; fecal incontinence; hospitalization indicated; severe increase in ostomy output compared to baseline; limiting self care activities of daily life | | Life-threatening consequences; urgent intervention indicated. | | | Death | |  |
| Definition: A disorder characterized by an increase in frequency and/or loose or watery bowel movements. | | | | | | | | | | | | |  |
| **Cardiac disorders** | | | | | | | | | | | | | |
| Chest pain - cardiac | Mild pain | | Moderate pain; limiting instrumental activities of daily life | | | | Pain at rest; limiting self care activities of daily life | — | | — | | | |
| Definition: A disorder characterized by substernal discomfort due to insufficient myocardial oxygenation. | | | | | | | | | | | | | |
| Conduction disorder | Mild symptoms; intervention not indicated | | Moderate symptoms | | | | Severe symptoms, intervention indicated | Life-threatening consequences; urgent intervention indicated. | | Death | | | |
| Definition: A disorder characterized by pathological irregularities in the cardiac conduction system. | | | | | | | | | | | | | |
| Cardiac failure | Asymptomatic with laboratory (e.g., BNP [B-type natriuretic peptide]) or cardiac imaging abnormalities | | Symptoms with mild to moderate activity or exertion | | | | Severe symptoms at rest or with minimal activity or exertion; intervention indicated | Life-threatening consequences; urgent intervention indicated (e.g., continuous IV therapy or mechanical hemodynamic support) | | Death | | | |
| Definition: A disorder characterized by the inability of the heart to pump blood at an adequate volume to meet tissue metabolic requirements, or, the ability to do so only at an elevation in the filling pressure. | | | | | | | | | | | | | |
| Left ventricular systolic dysfunction | — | — | | | Symptomatic due to drop in ejection fraction responsive to intervention | | | Refractory or poorly controlled heart failure due to drop in ejection fraction; intervention such as ventricular assist device, intravenous vasopressor support, or heart transplant indicated | | | Death | | |
| Definition: A disorder characterized by failure of the left ventricle to produce adequate output despite increases in diastolic pressure and end-diastolic volume. Clinical manifestations include signs such as dyspnea and orthopnea, as well as pulmonary congestion and pulmonary edema. | | | | | | | | | | | | | |
| **Nervous system disorders** | | | | | | | | | | | | | |
| Neuralgia | Mild pain | | Moderate pain; limiting instrumental activities of daily life | | | | Severe pain; limiting self care activities of daily life | — | | — | | | |
| Definition: A disorder characterized by intense painful sensation along a nerve or group of nerves. | | | | | | | | | | | | | |
| Paresthesia | Mild symptoms | | Moderate symptoms; limiting instrumental activities of daily life | | | | Severe symptoms; limiting self care activities of daily life | — | | — | | | |
| Definition: A disorder characterized by functional disturbances of sensory neurons resulting in abnormal cutaneous sensations of tingling, numbness, pressure, cold, and/or warmth. | | | | | | | | | | | | | |
| **Respiratory, thoracic, and mediastinal disorders** | | | | | | | | | | | | | |
| Cough | Mild symptoms; nonprescription intervention indicated | | Moderate symptoms, medical intervention indicated; limiting instrumental activities of daily life | | | | Severe symptoms; limiting self care activities of daily life | — | | — | | | |
| Definition: A disorder characterized by sudden, repetitive, spasmodic contraction of the thoracic cavity, resulting in violent release of air from the lungs and usually accompanied by a distinctive sound. | | | | | | | | | | | | | |
| Dyspnea | Shortness of breath with moderate exertion | | Shortness of breath with minimal exertion; limiting instrumental activities of daily life | | | | Shortness of breath at rest; limiting self care activities of daily life | Life-threatening consequences; urgent intervention indicated. | | Death | | | |
| Definition: Dyspnea. | | | | | | | | | | | | | |
| Epistaxis | Mild symptoms; intervention not indicated | | Moderate symptoms; medical intervention indicated (e.g., nasal packing, cauterization; topical vasoconstrictors) | | | | Transfusion; radiation, endoscopy, or surgery (e.g., hemostasis of bleeding site) | Life-threatening consequences; urgent intervention indicated. | | Death | | | |
| Definition: A disorder characterized by bleeding from the nose. | | | | | | | | | | | | | |
| Hiccups | Mild symptoms; intervention not indicated | | Moderate symptoms, intervention indicated; limiting instrumental activities of daily life | | | | Severe symptoms; interfering with sleep; limiting self care activities of daily life | — | | — | | | |
| Definition: A disorder characterized by repeated gulp sounds that result from an involuntary opening and closing of the glottis. This is attributed to a spasm of the diaphragm. | | | | | | | | | | | | | |
| **Skin and subcutaneous tissue disorders** | | | | | | | | | | | | | |
| Hand-foot syndrome | Minimal skin changes or dermatitis  (erythema, edema, or hyperkeratosis) without pain | | Skin changes (e.g., peeling, blisters, bleeding, fissures, edema, or hyperkeratosis) with pain; limiting instrumental ADL | | | | Severe skin changes (e.g., peeling, blisters, bleeding, edema, or hyperkeratosis) with pain; limiting self care ADL | - | | - | | | |
| Alopecia | Hair loss of < 50% of normal for that individual that is not obvious from a distance but only on close inspection. A different hair style may be required to cover the hair loss but it does not require a wig or hair piece to camouflage. | | Hair loss of > 50% normal for that individual that is readily apparent to others; a wig or hair piece is necessarily associated with psychosocial impact. | | | | — | — | | — | | | |
| Definition: A disorder characterized by a decrease in density of hair compared to normal for a given individual at a given age and body location. | | | | | | | | | | | | | |
| Dry skin | Covering <10% BSA and no associated erythema or pruritus | | Covering 10–30% BSA and associated with erythema or pruritus; limiting instrumental ADL | | | Covering > 30% BSA and associated with pruritus; limiting self care ADL | | — | | — | | | |
| Definition: A disorder characterized by flaky and dull skin; the pores are generally fine, the texture is a papery thin texture. | | | | | | | | | | | | | |
| **Vascular disorders** | | | | | | | | | | | | | |
| Hypertension | Prehypertension (Systolic BP 120–139 mm Hg, diastolic BP 80–89 mm Hg) | | Stage I Hypertension (Systolic BP 140–159 mm Hg, diastolic BP 90–99 mm Hg); medical intervention indicated; recurrent or persistent (≥ 24 h); symptomatic increase by > 20 mm Hg (diastolic) or to > 140/90 mmHg; monotherapy indicated initiated; Pediatric: Recurrent or persistent (≥ 24 h), BP > ULN; monotherapy indicated | | | Stage II Hypertension ( Systolic BP ≥ 160 mm Hg or diastolic BP ≥ 100 mm Hg); medical intervention indicated; more than one drug indicated; Pediatric: same as for adults | | Life-threatening consequences (e.g., malignant hypertension, transient or permanent neurologic deficit, hypertensive crisis); urgent intervention indicated; Pediatric: same as for adults | Death | | | | |
| Definition: A disorder characterized by a pathological increase in blood pressure. Repeated measurement showed the result as over 140/90 mmHg. | | | | | | | | | | | | | |
| Hypotension | Asymptomatic; intervention not indicated | | Non-urgent medical intervention indicated | | | Medical intervention indicated; hospitalization indicated | | Life-threatening consequences; urgent intervention indicated. | Death | | | | |
| Definition: A disorder characterized by a blood pressure that is below the normal. | | | | | | | | | | | | | |
| **Investigations** | | | | | | | | | | | | | |
| Elevated ALT | > ULN–3.0 × ULN | | | Asymptomatic: > 3.0–5.0 × ULN; > 3.0 × ULN with the worsening of the following symptoms: fatigue, nausea, vomiting, right upper quadrant pain or pressure, fever, rash, and eosinophilia | | > 5.0–20.0 × ULN; persistent for more than 2 weeks, > 5.0 × ULN | | > 20.0 × ULN | | | | — | |
| Definition: A finding based on laboratory test results that indicate an increase in the level of alanine aminotransferase (ALT or SGPT) in the blood specimen. | | | | | | | | | | | | | |
| Alkaline phosphatase increased | > ULN–2.5 × ULN | | | > 2.5–5.0 × ULN | | > 5.0–20.0 × ULN | | > 20.0 × ULN | | | | — | |
| Definition: A finding based on laboratory test results that indicate an increase in the level of alkaline phosphatase in a blood specimen. | | | | | | | | | | | | | |
| AST increased | > ULN–3.0 × ULN | | | Asymptomatic: > 3.0–5.0 × ULN; > 3.0 × ULN with the worsening of the following symptoms: fatigue, nausea, vomiting, right upper quadrant pain or pressure, fever, rash, and eosinophilia | | > 5.0–20.0 × ULN; persistent for more than 2 weeks, > 5.0 × ULN | | > 20.0 × ULN | | | | — | |
| Definition: A finding based on laboratory test results that indicate an increase in the level of aspartate aminotransferase (AST or SGOT) in the blood specimen. | | | | | | | | | | | | | |
| CPK increased | >ULN–2.5 × ULN | | >2.5 × ULN–5 × ULN | | | | >5 × ULN–10 × ULN | > 10 × ULN | | — | | | |
| Definition: A finding based on laboratory test results that indicate an increase in levels of creatine phosphokinase in a blood specimen. | | | | | | | | | | | | | |
| Creatinine increased | > 1–1.5 × baseline; > ULN–1.5 × ULN | | > 1.5–3.0 × baseline; > 1.5–3.0 × ULN | | | | > 3.0 × baseline; > 3.0–6.0 × ULN | > 6.0 × ULN | | — | | | |
| Definition: A finding based on laboratory test results that indicate increased levels of creatinine in a biological specimen. | | | | | | | | | | | | | |
| Ejection fraction decreased | — | | Resting ejection fraction (EF) 50–40%; 10–19% drop from baseline | | | | Resting ejection fraction (EF) 39–20%; > 20 % drop from baseline | Resting ejection fraction (EF) < 20% | | — | | | |
| Definition: The percentage computed when the amount of blood ejected during a ventricular contraction of the heart is compared to the amount that was present prior to the contraction. | | | | | | | | | | | | | |
| ECG QT interval prolonged | QTc 450–480 ms | | QTc 481–500 ms | | | | QTc ≥ 501 ms on at least two ECGs | QTc ≥ 501 ms; > 60 ms change from baseline and torsade de pointes or signs/symptoms of serious arrhythmia | | — | | | |
| Definition: A finding of a cardiac dysrhythmia characterized by an abnormally long QT interval. | | | | | | | | | | | | | |
| Lymphocyte count decreased | < LLN - 800/mm^3^; < LLN × 0.8–10e9 /L | | < 800–500/mm^3^; < 0.8–0.5 × 10e9 /L | | | | < 500–200/mm^3^; < 0.5–0.2 × 10e9 /L | < 200/mm^3^; < 0.2 × 10e9 /L | | — | | | |
| Definition: A decrease in number of lymphocytes in a blood specimen. | | | | | | | | | | | | | |
| Lymphocyte count increased | — | | > 4000/mm^3^–20,000/mm^3^ | | | | > 20,000/mm^3^ | — | | — | | | |
| Definition: An increase in the number of lymphocytes in the blood, effusions and bone marrow. | | | | | | | | | | | | | |
| Neutrophil count decreased | < LLN–1500/mm^3^; < LLN–1.5 × 10e9 /L | | < 1500–1000/mm^3^; < 1.5–1.0 × 10e9 /L | | | | < 1000–500/mm^3^; < 1.0–0.5 × 10e9 /L | < 500/mm^3^; < 0.5 × 10e9 /L | | — | | | |
| Definition: A finding based on laboratory test results that indicate a decrease in number of neutrophils in a blood specimen. | | | | | | | | | | | | | |
| Platelet count decreased | < LLN–75,000/mm^3^; < LLN–75.0 × 10e9 /L | | < 75,000–50,000/mm^3^; < 75.0–50.0 × 10e9 /L | | | | < 50,000–25,000/mm^3^; < 50.0–25.0 × 10e9 /L | < 25,000/mm^3^; < 25.0 × 10 e9/L | | — | | | |
| Definition: A finding based on laboratory test results that indicate a decrease in number of platelets in a blood specimen. | | | | | | | | | | | | | |
| Vital capacity abnormal | 90–75% of the predicted value | | < 75–50% of the predicted value, limiting instrumental activities of daily life | | | | < 50% of the predicted value; limiting self care activities of daily life | — | | — | | | |
| Definition: A finding based on pulmonary function test results that indicate an abnormal vital capacity (amount of exhaled after a maximum inhalation). | | | | | | | | | | | | | |
| Weight loss | 5% to < 10% from baseline, intervention not indicated | | 10% to < 20% from baseline, nutritional support indicated | | | | ≥ 20% from baseline; tube feeding or TPN indicated | — | | — | | | |
| Definition: A decrease in overall body weight; for pediatrics, less than the baseline growth curve. | | | | | | | | | | | | | |
| White blood cell decreased | < LLN–3000/mm^3^; < LLN–3.0 × 10e9 /L | | < 3000–2000/mm^3^; <3.0–2.0 × 10e9 /L | | | | < 2000–1000/mm^3^; < 2.0–1.0 × 10e9 /L | < 1000/mm^3^; < 1.0 × 10e9 /L | | — | | | |
| Definition: A finding based on laboratory test results that indicate a decrease in the number of white blood cells in a blood specimen. | | | | | | | | | | | | | |

Appendix II: ECOG Performance Status

| Scoring | Performance Status |
| --- | --- |
| **0** | Fully active, able to carry on all pre-disease performance without restriction |
| **1** | Restricted in physically strenuous activity but ambulatory and able to carry out light or office work |
| **2** | Ambulatory and capable of all self-care but unable to carry out any work activities; up and about less than 50% of waking hours |
| **3** | Capable of only limited self-care; confined to bed or chair more than 50% of waking hours |
| **4** | Completely disabled; cannot carry on any self-care; totally confined to bed or chair |
| **5** | Death |

Appendix III: Response Evaluation Criteria in Solid Tumors Version 1.1

(Response Evaluation Criteria in Solid Tumors RECIST Version 1.1)

1 MEASURABILITY OF TUMOR AT BASELINE

1.1 Definitions

At baseline, tumor lesions/lymph nodes will be categorized as measurable or non-measurable as follows:

1.1.1 Measurable lesions

Tumor lesions: Must be accurately measured in at least one dimension (longest diameter in the plane of measurement is to be recorded) with a minimum size of:

- 10 mm by CT scan (CT scan slice thickness no greater than 5 mm)
- 10 mm caliper measurement by clinical exam (lesions which cannot be accurately measured with calipers should be recorded as non-measurable).
- 20 mm by chest X-ray.
- Malignant lymph nodes: To be considered pathologically enlarged and measurable, a lymph node must be ≥15 mm in short axis when assessed by CT scan (CT scan slice thickness recommended to be no greater than 5 mm). At baseline and in follow-up, only the short axis will be measured and followed.

1.1.2 Non-measurable

All other lesions, including small lesions (longest diameter < 10 mm or pathological lymph nodes with ≥ 10 to < 15 mm short axis) as well as truly non-measurable lesions. Lesions considered truly non-measurable include: leptomeningeal disease, ascites, pleural or pericardial effusion, inflammatory breast disease, lymphangitic involvement of skin or lung, abdominal masses/abdominal organomegaly identified by physical exam that is not measurable by reproducible imaging techniques.

1.1.3 Special considerations regarding lesion measurability

Bone lesions, cystic lesions, and lesions previously treated with local therapy require particular comment:

Bone lesions:

- Bone scan, PET scan or plain films are not suitable for measuring bone lesions, but these techniques can be used to confirm the presence or disappearance of bone lesions;
- If lytic bone lesions or mixed lytic-blastic lesions, with identifiable soft tissue components, can be evaluated by cross sectional imaging techniques such as CT or MRI, and the soft tissue component meets the definition of measurability described above, then the lesions can be considered as measurable lesions.;
- Blastic bone lesions are non-measurable.

Cystic lesions:

- Lesions that meet the criteria for radiographically defined simple cysts should not be considered as malignant lesions (neither measurable nor non-measurable) since they are, by definition, simple cysts.
- 'Cystic lesions' thought to represent cystic metastases can be considered as measurable lesions if they meet the definition of measurability described above. However, if non-cystic lesions are present in the same patient, these are preferred for selection as target lesions.

Lesions with prior local treatment:

- Tumor lesions situated in a previously irradiated area, or in an area subjected to other loco-regional therapy, are usually not considered measurable unless there has demonstrated progression in the lesion. Study protocols should detail the conditions under which such lesions would be considered measurable.

1.2 Specifications by Methods of Measurements

1.2.1 Measurement of lesions

All measurements should be recorded in metric notation, using calipers if clinically assessed. All baseline evaluations should be performed as close as possible to the treatment start and never more than 28 days (4 weeks) before the beginning of the treatment.

1.2.2 Method of assessment

The same method and technique of assessment should be used to characterize each identified and reported lesion at baseline and during follow-up. Imaging evaluation should always be done rather than clinical examination unless the lesion(s) being followed cannot be imaged but can be assessed by clinical exam.

Clinical lesions: Clinical lesions will only be considered measurable when they are superficial and ≥ 10 mm in diameter as assessed using calipers (e.g. skin nodules). For the case of skin lesions, documentation by color photography including a ruler to estimate the size of the lesion is suggested. As noted above, when lesions can be evaluated by both clinical exam and imaging, imaging evaluation should be chosen whenever possible since it is more objective and may also be reviewed at the end of the study.

Chest X-ray: Chest CT is preferred over chest X-ray, particularly when progression is an important endpoint, since CT is more sensitive than X-ray, particularly in identifying new lesions. However, lesions on chest X-ray may be considered measurable if they are clearly defined and surrounded by aerated lung.

CT, MRI: CT is currently best available and reproducible method to measure lesions selected for response assessment. This guideline has defined measurability of lesions on CT scan based on the assumption that CT slice thickness is ≤ 5 mm. When CT slice thickness is greater than 5 mm, the minimum size for a measurable lesion should be twice of the slice thickness. MRI is also acceptable in certain situations (e.g. for body scans).

Ultrasound: Ultrasound should not be used as a method of measurement in assessment of lesion size. Ultrasound examination is not reproducible for independent review at a later date and, because they are operator dependent, it cannot be guaranteed that the same technique and measurements will still be suitable from one assessment to the next. If new lesions are identified by ultrasound in the course of the trial, confirmation by CT or MRI is advised. If there is concern about radiation exposure at CT, MRI may be used instead of CT in selected instances.

Endoscopy, laparoscopy: The utilization of these techniques for objective tumor evaluation is not advised. However, they can be used to confirm complete response when biopsies are obtained or to determine relapse in trials where recurrence following complete response or surgical resection is an endpoint.

Tumor markers: Tumor markers alone cannot be used to assess objective tumor response. If markers are initially above the upper normal limit, however, they must normalize for a patient to be considered in complete response. Because tumor markers are disease specific, instructions for their measurement should be incorporated into protocols on such a basis. Specific guidelines for both CA-125 response (in recurrent ovarian cancer) and PSA response (in recurrent prostate cancer) have been published. In addition, the Gynecologic Cancer Intergroup has developed CA-125 progression criteria which are to be added into objective tumor assessment criteria for first-line trials in ovarian cancer.

Cytology/histology: These techniques can be used to differentiate PR and CR in specific cases required by protocol (e.g. residual benign tumors can always remain in lesions of tumor types such as germ cells). When effusions are possible to be a potential adverse effect of treatment (e.g., with certain taxane compounds or angiogenesis inhibitors) and the measurable tumor has met criteria for response or stable disease, the cytological confirmation of the neoplastic origin of any effusion that appears or worsens during treatment can be considered, in order to differentiate response (or stable disease) and disease progression.

2 TUMOR RESPONSE EVALUATION

2.1 Assessment of Overall Tumor and Measurable Lesions

To assess objective response or future progression, it is necessary to estimate the overall tumor burden at baseline and use this as a comparator for subsequent measurements. Only patients with measurable lesions at baseline can be included in protocols where objective tumor response is the primary endpoint. Measurable lesion is defined as the presence of at least one measurable lesion. In trials where the primary endpoint is tumor progression (either time to progression or proportion with progression at a fixed date), the protocol must specify if entry is restricted to those with measurable lesions or whether patients having non-measurable lesions only are also eligible.

2.2 Baseline Documentation of Target and Non-Target Lesions

When more than one measurable lesion is present at baseline, all lesions up to a maximum of five lesions total (and a maximum of two lesions per organ) representative of all involved organs should be identified as target lesions and will be recorded and measured at baseline (this means in instances where patients have only one or two organ sites involved a maximum of two and four lesions respectively will be recorded).

Target lesions should be selected on the basis of their size (lesions with the longest diameter), be representative of all involved organs, but in addition should be those that lend themselves to reproducible repeated measurements. It may be the case that, on occasion, the largest lesion does not lend itself to reproducible measurement, in which circumstance the next largest lesion which can be measured reproducibly should be selected.

Lymph nodes merit special mention since they are normal anatomical structures which may be visible by imaging even if not involved by tumor. Pathological nodes defined as measurable and may be identified as target lesions must meet the criterion of a short axis of ≥ 15 mm by CT scan. Only the short axis of these nodes will be measured at the baseline. The short axis of the node is normally used by radiologists to determine if a node is involved by solid tumor. Nodal size is normally reported as two dimensions in the plane in which the image is obtained (for CT scan this is almost always the axial plane; for MRI the plane of acquisition may be axial, sagittal or coronal). The minimum of these measurements is the short axis. For example, an abdominal node which is reported as being 20 mm × 30 mm has a short axis of 20 mm and can be qualified as a malignant, measurable node. In this example, 20 mm should be recorded as the measurement value of node. All other pathological nodes (those with short axis ≥ 10 mm but < 15 mm) should be considered non-target lesions. Nodes that have a short axis < 10 mm are considered non-pathological and should not be recorded or followed.

A sum of the diameters (longest for non-nodal lesions, short axis for nodal lesions) for all target lesions will be calculated and reported as the baseline sum diameters. If lymph nodes are to be included in the sum, then as noted above, only the short axis is added into the sum. The baseline sum diameters will be used as reference for the baseline.

All other lesions (or sites of disease) including pathological lymph nodes should be identified as non-target lesions and should also be recorded at baseline. Measurements are not required and these lesions should be followed as 'present', 'absent', or in rare cases 'unequivocal progression'. In addition, it is possible to record multiple target lesions involving the same organ as a single item on the case record form (e.g. 'multiple enlarged pelvic lymph nodes' or 'multiple liver metastases').

2.3 Response Criteria

2.3.1 Evaluation of target lesions

Complete response (CR): Complete disappearance of the target lesion, any pathological lymph nodes (whether target or non-target) must have reduction in short axis to < 10 mm.

Partial response (PR): At least a 30% decrease relative to baseline in the sum of diameters of target lesions.

Progressive disease (PD): At least a 20% increase in the sum of diameters of target lesions, taking as reference the smallest sum on study (this includes the baseline sum if that is the smallest on study). In addition, the sum must also demonstrate an absolute increase of at least 5 mm (the appearance of one or more new lesions is also considered progression).

Stable Disease (SD): Neither sufficient shrinkage to qualify for PR nor sufficient increase to qualify for PD, taking as reference the smallest sum diameters while on study.

2.3.2 Notifications on the assessment of target lesions

Lymph nodes: Lymph nodes identified as target lesions should always have the actual short axis measurement recorded (measured in the same anatomical plane as the baseline examination), even if the nodes regress to below 10 mm on study. This means that when lymph nodes are included as target lesions, the sum of lesions may not be zero even if complete response criteria are met, since a normal lymph node is defined as having a short axis of < 10 mm. Target lymph nodal lesions should be recorded in a specific section of the case report form or other data collection methods: each node must achieve a short axis of < 10 mm for CR; For PR, SD and PD, the actual short axis measurement of the nodes is to be included in the sum of diameters of target lesions.

Target lesions that become too small to be measured. While on study, all lesions (nodal and non-nodal) recorded at baseline should have their actual measurements recorded at each subsequent evaluation, even they are very small (e.g. 2 mm). However, sometimes lesions or lymph nodes which are recorded as target lesions at baseline become so faint on CT scan that the radiologist may find it hard to define the exact value and may report them as being "too small to be measured". In such a case, it is important that a value be recorded on the case report form. If it is the opinion of the radiologist that the lesion has likely disappeared, the measurement should be recorded as 0 mm. If the lesion is believed to be present and is faintly seen but too small to be measured, a default value of 5 mm should be assigned. (Note: It is less likely that this rule will be used for lymph nodes since they usually have a definable size when normal and are frequently surrounded by fat such as in the retroperitoneum; however, if a lymph node is believed to be present and is faintly seen but too small to be measured, a default value of 5 mm should be assigned as well). This default value is derived from the 5 mm CT slice thickness (but should not be changed with varying CT slice thickness). The measurement of these lesions is potentially non-reproducible, therefore providing this default value will reduce the risk of measurement error. To reiterate, however, if the radiologist is able to provide an actual value of the lesion size, that should be recorded, even if it is below 5 mm.

Lesions that split or coalesce on treatment: When non-nodal lesions split into fragment, the longest diameters of the fragmented portions should be added together to calculate the target lesion sum. Similarly, as coalesced lesions, a plane between them may be maintained that would aid in obtaining maximal diameter measurements of each lesion. If the lesions have truly coalesced such that they are no longer separable, the vector of the longest diameter in this instance should be the maximal longest diameter for the coalesced lesion.

2.3.3 Evaluation of non-target lesions

This section provides the definitions of the criteria used to determine the tumor response for the group of non-target lesions. While some non-target lesions may actually be measurable, they need no measurements and instead should be assessed only qualitatively at the time points specified in the protocol.

Complete Response (CR): Disappearance of all non-target lesions and normalization of tumor marker level. All lymph nodes must be non-pathological in size (with < 10 mm short axis).

Non-CR/Non-PD: Persistence of one or more non-target lesion(s) and/or maintenance of tumor marker level above the normal limits.

Progressive Disease (PD): Unequivocal progression of existing non-target lesions. Note: the appearance of one or more new lesions is also considered as progression.

2.3.4 Special notes on assessment of progression of non-target lesions

The concept of progression of non-target lesions requires additional explanation as follows: When the patient has measurable non-target lesions, there must be an overall level of substantial worsening in non-target disease such that, even in presence of SD or PR in target disease, the overall tumor burden has increased sufficiently to merit discontinuation of therapy to achieve "unequivocal progression" on the basis of the non-target lesions. A modest increase in the size of one or more non-target lesions is usually insufficient to meet the unequivocal progression criteria. The designation of overall progression solely on the basis of change in non-target disease in the face of SD or PR of target disease will therefore be extremely rare.

The case of the patient having only non-measurable lesions arises in some phase III trials when there is not a criterion of study entry to have measurable lesions. The same general concepts apply here as noted above, however, in this instance there is no measurable disease assessment to factor into the interpretation of an increase in non-measurable disease burden. Because worsening in non-target disease cannot be easily quantified (by definition: if all lesions are truly non-measurable), a useful test can be applied to assess unequivocal progression if the increase in overall disease burden based on the change in non-measurable disease is comparable in magnitude to the increase that would be required to declare PD for measurable disease. E.g., an increase in tumor burden representing an additional 73% increase in 'volume' (which is equivalent to a 20% increase in diameter in a measurable lesion). Examples include an increase in an ascites from 'trace' to 'large', an increase in lymphangitic disease from localized to widespread, or may be described in protocols as "sufficient to change therapy". Examples include an increase in a pleural effusion from trace to large, an increase in lymphangitic disease from localized to widespread, or may be described in protocols as "necessary to change the therapy". If unequivocal progression is seen, the patient should be considered to have had overall PD at that point. While it would be ideal to have objective criteria to apply to non-measurable lesions, the very nature of that disease makes it impossible to do so, and therefore the increase must be substantial.

2.3.5 New lesions

The appearance of new malignant lesions denotes disease progression; therefore, some comments on detection of new lesions are important. There are no specific criteria for the imaging identification of new radiographic lesions; however, the finding of a new lesion should be unequivocal. e.g. disease progression should not attribute to differences in scanning technique, change in imaging modality or findings thought to represent something other than tumor (for example, some ‘new’ bone lesions may be simply healing or relapse of pre-existing lesions). This is particularly important when the patient's baseline lesions show partial or complete response. For example, necrosis of a liver lesion may be reported on a CT scan report as a new cystic lesion, but it actually is not.

A lesion identified on a follow-up study in an anatomical location that was not scanned at baseline is considered to be a new lesion and will indicate disease progression. An example of this is the patient who has visceral lesion at baseline and while on study has a CT or MRI brain examination which reveals metastases. The patient’s brain metastases are considered to be evidence of PD even if he/she did not have brain imaging at baseline.

If a new lesion is equivocal, for example, because of its small size, continued therapy and follow-up evaluation will be needed to clarify if it represents a truly new lesion. If repeat scans confirm there is definitely a new lesion, then progression should be declared since the date of the initial scan.

While FDG-PET response assessments need additional study, it is sometimes reasonable to incorporate the use of FDG-PET scanning to complement CT scanning in assessment of progression (particularly possible ‘new’ disease). New lesions on the basis of FDG-PET imaging can be identified according to the following procedures:

Negative FDG-PET at baseline, with a positive FDG-PET at follow-up being a sign of PD.

No FDG-PET at baseline and a positive FDG-PET at follow-up:

If the positive FDG-PET at follow-up corresponds to a new site of disease confirmed by CT, this is PD.

If the positive FDG-PET at follow-up is not confirmed as a new site of disease on CT, additional follow-up CT scans are needed to determine if there is truly progression occurring at that site (if so, the date of PD will be the date of the initial abnormal FDG-PET scan).

If the positive FDG-PET at follow-up corresponds to an existing site of lesion on CT that is not progressing on the basis of the anatomic images, this is not PD.

2.4 Evaluation of Best Overall Response

The best overall response is the best response recorded from the start of the study treatment until the end of treatment, taking any requirement for confirmation into account. On occasion a response may appear after the end of therapy, so protocols should be clear if post-treatment assessments are to be considered in determination of best overall response. Protocols must specify how any new therapy introduced before progression will affect best response assessment. The patient’s best response assessment will depend on the findings of both target and non-target lesions and will also take the appearance of new lesions into consideration. Furthermore, it also depends on the nature of the study, the protocol requirements, and confirmatory measurement. Specifically, in non-randomized trials where response is the primary endpoint, confirmation of PR or CR is necessary to determine which one the ‘best overall response’.

2.4.1 Time point response

It is assumed that a response assessment occurs at each protocol specified time point. Table 1 on the next page provides a summary of the overall response status calculation at each time point for patients who have measurable disease at baseline.

When patients have non-measurable (therefore non-target) disease only, Table 2 is to be referenced.

2.4.2 Missing assessments and not evaluable designation

When no imaging/measurement is done at all at a particular time point, the patient is not evaluable (NE) at that time point. If only a subset of lesion measurements are made at an assessment, the case is usually considered NE at that time point, unless a convincing argument can be made that the contribution of the individual missing lesion(s) would not change the assigned time point response assessment. This would be most likely to happen in the case of PD. For example, if a patient had a baseline sum of 50 mm with three measured lesions, but at follow-up only two lesions were assessed with a sum of 80 mm, the patient would have achieved PD status, regardless of the contribution of the missing lesion.

2.4.3 Best overall response: all time points

The best overall response is determined once all the data for the patient are known.

Best response determination in trials where confirmation of complete or partial response is not required: Best response in these trials is defined as the best response across all time points (for example, a patient who has SD at first assessment, PR at second assessment, and PD on last assessment has a best overall response of PR). When SD is believed to be best response, it must meet the minimum time from baseline as specified in the protocol. If the minimum time is not met when SD is otherwise the best time point response, the patient’s best response depends on the subsequent assessments. For example, a patient who has SD at first assessment, PD at second and does not meet minimum duration for SD will have a best response of PD. The same patient lost to follow-up after the first SD assessment would be considered to be not evaluable.

Best response determination in trials where confirmation of complete or partial response is required: Complete or partial responses may be claimed only if the criteria for each are met at a subsequent time point as specified in the protocol (generally 4 weeks later). In such a circumstance, the best overall response can be interpreted as in Table 3.

2.4.4 Special notes on response assessment

When nodal disease is included in the sum of target lesions and the nodes decrease to "normal" size (< 10 mm), they may still have a measurement reported on scans. This measurement should be recorded even though the nodes are normal in order not to overstate progression should it be based on increase in size of the nodes. As noted aforesaid, this means that patients with CR may not have a total sum of 'zero' on the case report form (CRF).

In trials where confirmation of response is required, repeated "NE" time point assessments may complicate best response determination. The analysis plan for the trial must specify how missing data/assessments will be addressed in determination of response. For example, in most trials it is reasonable to consider a patient with time point responses of PR-NE-PR as a confirmed response.

When patients have a global deterioration of health status requiring discontinuation of treatment, but there is no objective evidence of disease progression at that time, it should be reported as 'symptomatic deterioration'. Every effort should be made to evaluate objective progression even after discontinuation of treatment. Symptomatic deterioration is not a descriptor of an objective response: It is a reason for stopping study therapy. The objective response status of such patients is to be determined by evaluation of target and non-target lesions as shown in Tables 1–3.

Conditions defined as early progression, early death, and NE are study specific and should be clearly described in each protocol (depending on treatment duration and treatment periodicity).

In some circumstances, it may be difficult to distinguish residual lesion from normal tissues. When the evaluation of complete response depends upon such a definition, it is recommended that the residual lesion be investigated (fine needle aspirate/biopsy) before assigning a status of complete response. FDG-PET may be used to upgrade a response to a CR in a manner similar to a biopsy in cases where a residual radiographic abnormality is thought to represent fibrosis or scarring. The use of FDG-PET in this circumstance should be prospectively described in the protocol and supported by disease specific medical literature for the indication. However, it must be acknowledged that both approaches may lead to false positive CR due to limitations of FDG-PET and biopsy resolution/sensitivity.

**Table 1. Time point response: patients with target (+/– non-target) lesions.**

| **Target lesions** | **Non-target lesions** | **New lesions** | **Overall response** |
| --- | --- | --- | --- |
| CR | CR | Non- | CR |
| CR | Non-CR/Non-PD | Non- | PR |
| CR | Not evaluated | Non- | PR |
| PR | Non-PD or not all evaluated | Non- | PR |
| SD | Non-PD or not all evaluated | Non- | SD |
| Not all evaluated | Non-PD | Non- | NE |
| PD | Any | Yes or No | PD |
| Any | PD | Yes or No | PD |
| Any | Any | Yes | PD |
| CR = complete response, PR = partial response, SD = stable disease, PD = progressive disease, and NE = not evaluable. | | | |

**Table 2. Time point response: patients with non-target lesions only**

| **Non-target lesions** | **New lesions** | **Overall response** |
| --- | --- | --- |
| CR | Non- | CR |
| Non-CR or Non-PD | Non- | Non-CR or Non-PD |
| Not all evaluated | Non- | Not evaluated |
| Unequivocal PD | Yes or No | PD |
| Any | Yes | PD |

Note: "Non-CR/non-PD" is preferred over SD for non-target disease. Since SD is increasingly used as an endpoint for assessment of efficacy in some trials, so to assign this category when no lesions can be measured is not advised.

For equivocal findings of progression (e.g., very small and uncertain new lesions; cystic changes or necrosis in existing lesions), treatment may continue until the next scheduled assessment. If progression is confirmed at the next scheduled assessment, the date of progression should be the earlier date when progression was suspected.

**Table 3. Best overall response when confirmation of CR and PR required**

| **Overall response at first time point** | **Overall response subsequent time point** | **Best overall response** |
| --- | --- | --- |
| CR | CR | CR |
| CR | PR | SD, PD or PR^a^ |
| CR | SD | SD provided minimum criteria for SD duration met, otherwise, PD |
| CR | PD | SD provided minimum criteria for SD duration met, otherwise, PD |
| CR | NE | SD provided minimum criteria for SD duration met, otherwise, NE |
| PR | CR | PR |
| PR | PR | PR |
| PR | SD | SD |
| PR | PD | SD provided minimum criteria for SD duration met, otherwise, PD |
| PR | NE | SD provided minimum criteria for SD duration met, otherwise, NE |
| NE | NE | NE |

Note: CR = complete response, PR = partial response, SD = stable disease, PD = progressive disease, NE = not evaluable. ^a^: If a CR is truly met at first time point, then any disease seen at a subsequent time point, even disease meeting PR criteria relative to baseline, makes the disease PD after that point (since disease must have reappeared after CR). Best response would depend on whether minimum duration for SD was met. However, sometimes 'CR' may be firstly claimed while subsequent scans suggest small lesions were likely still present, and thus in fact the patient had PR, not CR at the first time point. Under these circumstances, the original CR should be changed to PR and the best response is PR.

2.5 Frequency of Tumor Re-Evaluation

Frequency of tumor re-evaluation while on treatment should be protocol specific and adapted to the type and schedule of treatment. However, in the context of phase II studies where the beneficial effect of therapy is not known, follow-up every 6–8 weeks (timed to coincide with the end of a cycle) is reasonable. Smaller or greater time intervals than these could be justified in specific regimens or circumstances. The protocol should specify which organ sites are to be evaluated at baseline (usually those most likely to be involved with metastatic disease for the tumor type under study) and how often evaluations are repeated. Normally, all target and non-target lesions should be evaluated at each assessment. In selected circumstances, certain non-target organs may be evaluated less frequently, e.g. bone scans may need to be repeated only when complete response is identified in target disease or when progression in bone is suspected.

After the end of the treatment, the need for repetitive tumor evaluations depends on whether the response rate or the time to an event (progression/death) is considered as the trial endpoint. If time to an event (e.g. time to progression, disease-free survival, progression-free survival) is the main endpoint of the study, then routine scheduled re-evaluation of protocol specified sites of disease is warranted. In randomized comparative trials in particular, the scheduled assessments should be performed as identified on a calendar schedule (for example: every 6–8 weeks on treatment or every 3–4 months after treatment) and should not be affected by other factors, such as delays in therapy, drug dosing intervals or any other events that might lead to imbalance in a treatment arm in the timing of disease assessment.

2.6 Confirmation of Efficacy Measurement/Duration of Response

2.6.1 Confirmation

In non-randomized trials where response is the primary endpoint, confirmation of PR and CR is required to ensure efficacy identified are not the result of measurement error. This will also permit appropriate interpretation of results in the context of historical data where efficacy has traditionally required confirmation in such trials. However, in all other circumstances, e.g., in randomized trials (phase II or III) or studies where stable disease or progression are the primary endpoints, confirmation of efficacy is not required since it will not add value to the interpretation of trial results. However, elimination of the requirement for efficacy confirmation may increase the importance of central review to protect against bias, particularly in studies which are not blinded.

In the case of SD, measurements must have met the SD criteria defined in the study protocol at least once after study entry at a minimum interval (in general not less than 6–8 weeks).

2.6.2 Duration of overall response

The duration of overall response is measured from the time measurement criteria are first met for CR/PR (whichever is first recorded) until the first date that recurrent or progressive disease is objectively recorded (taking as reference for progressive disease the smallest measurements recorded on study). The duration of overall complete response is measured from the time measurement criteria are first met for CR until the first date that recurrent disease is objectively documented.

2.6.3 Duration of stable disease

Stable disease is measured from the start of the treatment (in randomized trials, from the date of randomization) until the progression, taking as reference the smallest sum on study (if the baseline sum is the smallest, this is the reference for calculation of PD). The clinical relevance of the duration of stable disease varies in different studies and diseases. If the proportion of patients achieving stable disease for a minimum period of time is an endpoint in a particular trial, the protocol should specify the minimal time interval required between two measurements for determination of stable disease.

Note: The duration of response and stable disease as well as the progression-free survival are influenced by the frequency of follow-up after baseline evaluation. It is not in the scope of this guideline to define a standard follow-up frequency. The follow-up frequency should take many parameters into account, including disease types and stages, treatment periodicity and standard practice, etc. However, these limitations of the precision of the measured endpoint should be taken into account if comparisons between trials are to be made.

2.7 PFS/TTP

2.7.1 Phase II clinical trials

This guideline focuses primarily on the use of objective response endpoints for Phase II clinical trials. In some circumstances, 'response rate' may not be the optimal method to assess the potential anticancer activity of new agents/regimens. In such cases, PFS or the PPF at landmark time points might be considered appropriate alternatives to provide an initial signal of biologic effect of new agents. It is clear, however, that in an uncontrolled trial, these measures are subject to criticism since an apparently promising observation may be related to biological factors such as patient selection and not the impact of drug intervention. Thus, Phase II clinical trials utilizing these endpoints are best designed with a randomized control. Exceptions may exist where the behavior patterns of certain cancers are so consistent (and usually consistently poor), that a non-randomized trial is justifiable. However, in these cases, it will be essential to document with care the basis for estimating the expected PFS or PPF in the absence of a positive control.

Appendix V: Deviation Table of Blood Collection Time Points

In this study, blood samples should be collected within the permitted time frame.

Permissible time deviation during blood collection: When the time interval between blood collection points is less than or equal to 1 h, the time deviation should be within ±5%. When the time interval between blood collection points is larger than 1 h, the time deviation should be within ±2.5%. See Table 1 for specific requirements for blood sampling time.

Any behavior not conforming to the permitted time frame for blood collection should be immediately recorded in the relevant original data table. During multiple blood collections, the indwelling catheter will be treated with 3–5 mL physiological saline solution and retained at the forearm vein. Therefore, blood containing physiological saline must be first removed during each blood sampling. Disposable sterile syringes and needles are used for blood sampling.

**Table 1. Blood sampling time window for single dosing**

| **Sampling time point (h)** | **Sampling time window (min)** |
| --- | --- |
| 0 | Within 30 min before dosing |
| 0.5 | 1.5 |
| 1 | 1.5 |
| 2 | 3 |
| 3 | 3 |
| 4 | 3 |
| 5 | 3 |
| 6 | 3 |
| 8 | 3 |
| 12 | 6 |
| 24 | 18 |
| 32 | 12 |
| 48 | 24 |
| 72 | 36 |

**Table 2. Blood sampling time window for repeated dosing**

| **Sampling time point (h)** | **Sampling time window (min)** |
| --- | --- |
| 0 | Within 30 min before dosing |
| 0.5 | 1.5 |
| 1 | 1.5 |
| 2 | 3 |
| 3 | 3 |
| 4 | 3 |
| 5 | 3 |
| 6 | 3 |
| 8 | 3 |
| 12 | 6 |
| 24 | 18 |

Appendix IV: Principles of Replacement Doses

During continuous administration, subjects should take the missed dose only if it is more than 20 hours from the next dose, and skip the missed dose if it is less than 20 hours from the next dose. The subject should take the next dose as scheduled.

In case of vomiting after taking the investigational product, the subject should not take an extra dose, and take the next dose as scheduled. If the vomiting persists, the subject should notify the investigator in a timely manner for treatment. Antiemetic prophylaxis is not used during the study. Antiemetics may be used if vomiting occurs and premedication may be used subsequently.

Missed doses due to toxicities should be skipped.
